# MRSD: A quantitative approach for assessing suitability of RNA-seq in the investigation of mis-splicing in Mendelian disease

**DOI:** 10.1016/j.ajhg.2021.12.014

**Published:** 2022-01-21

**Authors:** Charlie F. Rowlands, Algy Taylor, Gillian Rice, Nicola Whiffin, Hildegard Nikki Hall, William G. Newman, Graeme C.M. Black, kConFab Investigators, Raymond T. O’Keefe, Simon Hubbard, Andrew G.L. Douglas, Diana Baralle, Tracy A. Briggs, Jamie M. Ellingford

**Affiliations:** 1 Division of Evolution, Infection and Genomic Sciences, School of Biological Sciences, Faculty of Biology, Medicine and Health, University of Manchester, Manchester M13 9PT, UK; 2 Manchester Centre for Genomic Medicine, St Mary’s Hospital, Manchester University NHS Foundation Trust, Manchester Academic Health Science Centre, Manchester M13 9WL, UK; 3 Wellcome Centre for Human Genetics, University of Oxford, Oxford OX3 7BN, UK; 4 MRC Human Genetics Unit, Institute of Genetics and Cancer, University of Edinburgh, Edinburgh EH4 2XU, UK; 5 Sir Peter MacCallum Department of Oncology, The University of Melbourne, Parkville, VIC 3010, Australia; 6 kConFab, Research Department, Peter MacCallum Cancer Centre, Melbourne, VIC 3000, Australia; 7 Wessex Clinical Genetics Service, Princess Anne Hospital, University Hospital Southampton NHS Foundation Trust, Coxford Rd, Southampton SO16 5YA, UK; 8 Faculty of Medicine, University of Southampton, Duthie Building, Southampton General Hospital, Tremona Road, Southampton SO16 6YD, UK

**Keywords:** RNA-seq, RNA, gene expression, splicing, Mendelian disease, diagnostics, transcriptomics

## Abstract

Variable levels of gene expression between tissues complicates the use of RNA sequencing of patient biosamples to delineate the impact of genomic variants. Here, we describe a gene- and tissue-specific metric to inform the feasibility of RNA sequencing. This overcomes limitations of using expression values alone as a metric to predict RNA-sequencing utility. We have derived a metric, minimum required sequencing depth (MRSD), that estimates the depth of sequencing required from RNA sequencing to achieve user-specified sequencing coverage of a gene, transcript, or group of genes. We applied MRSD across four human biosamples: whole blood, lymphoblastoid cell lines (LCLs), skeletal muscle, and cultured fibroblasts. MRSD has high precision (90.1%–98.2%) and overcomes transcript region-specific sequencing biases. Applying MRSD scoring to established disease gene panels shows that fibroblasts, of these four biosamples, are the optimum source of RNA for 63.1% of gene panels. Using this approach, up to 67.8% of the variants of uncertain significance in ClinVar that are predicted to impact splicing could be assayed by RNA sequencing in at least one of the biosamples. We demonstrate the utility and benefits of MRSD as a metric to inform functional assessment of splicing aberrations, in particular in the context of Mendelian genetic disorders to improve diagnostic yield.

## Introduction

Pinpointing disease-causing genomic variation informs diagnosis, treatment, and management for a wide range of rare disorders. Pathogenic variants, both protein-coding and intronic, that lie outside canonical splice sites may nonetheless act to disrupt pre-mRNA splicing through a diverse series of mechanisms (Figure S1).^1^, ^2^, ^3^ Effective identification of pathogenic splice-impacting variants remains challenging and is limited by the omission of intronic regions in targeted sequencing approaches,^4^^,^^5^ discordance between *in silico* variant prioritization tools,^6^ and the lack of availability of the appropriate tissue from which to survey RNA for splicing disruption.^7^^,^^8^

Targeted analyses such as RT-PCR enable detection of splicing aberrations^3^ but are designed to test for the presence of specific disruptions. As such they may not identify the complete spectrum of splicing disruption caused by a single genomic variant. By contrast, RNA sequencing (RNA-seq) offers a potential route to identify aberrant splicing events without prior knowledge of the underlying genomic variants driving their impact.^3^^,^^9^, ^10^, ^11^, ^12^, ^13^ Further, there is growing evidence that RNA-seq can substantially improve diagnostic yield across a variety of disease subtypes^3^^,^^10^^,^^13^, ^14^, ^15^ through identification of variants impacting splicing or leading to impairment of transcript expression or stability.^16^

However, there remain several hurdles to the effective and routine integration of RNA-seq into diagnostic pipelines. For example, surveying a whole transcriptome identifies a large number of splicing events—in the order of hundreds of thousands. Despite a recent increase in the number of tools designed to scrutinize RNA-seq data for splicing outliers,^9^^,^^13^^,^^17^^,^^18^ there is little consensus regarding the best approach to filter true positive and pathogenic events from neutral or artifactual findings. Furthermore, diagnostic analysis using RNA-seq is only effective when sufficient levels of sequence coverage of a relevant gene transcript are present in the sampled tissue.

In this study, we develop an informatics approach to assess the suitability of RNA-seq derived from different tissues to identify pathogenic splicing aberrations in specific genes of interest (Figure S2). We name our framework the minimum required sequencing depth (MRSD), which can be utilized in a flexible and customized manner (Figure S2). MRSD scores (see web resources for access) can be utilized to select the most appropriate biosample to detect specific splicing aberrations and to guide required depth of sequencing.

## Material and methods

### Minimum required sequencing depth (MRSD) score

The MRSD model considers the level of sequencing coverage for splice junctions in tissue-specific reference sets (see Reference set generation from control RNA-seq data) and calculates the minimum required sequencing depth, in millions of uniquely mapping 75 bp reads, that would be required for the desired proportion of splice junctions in a given transcript to be covered by a desired number of sequencing reads. The model is dynamic and can be adjusted by the user to account for customized levels of desired sequencing coverage per splicing junction, the proportion of splicing junctions covered, and the “MRSD parameter” (_*m*_) which represents the proportion of control samples for which the returned MRSD holds true (suggested usage of 0.95 or 0.99).

MRSD is defined for an individual transcript in a given sample as:

$$MRSD_{m}=r/\left(\frac{R_{p}}{d}\right)$$

where *r* is the desired level of read coverage across desired proportion *p* of splice junctions, *R* is the set of read counts supporting each of the splice junctions in the transcript of interest, ordered from lowest to highest, and *R*_*p*_ is the read count at the position in *R* at which proportion *p* of read counts values in *R* are greater than or equal to it. *d* represents the total number of sequencing reads, in millions of reads, in the RNA-seq sample (by default, the number of uniquely mapping sequencing reads), and (*m*) represents the MRSD parameter. Where there is zero-read coverage of the critical number of splice junctions (i.e., where *R*_*p*_ = 0), no MRSD can be generated and surveying of the transcript is deemed “unfeasible” in the given tissue. Further elaboration and an illustrative example are given in supplemental material and methods S1.

### Hierarchical approach to transcript selection and investigation of impact of transcript selection on MRSD predictions

MRSD can be calculated for any transcript sets of interest. For the analyses described in this study, we generated a single transcript model for each gene in the GENCODE v19 human genome annotation (supplemental material and methods S2). We utilized a hierarchical approach for transcript selection, whereby we prioritized transcripts in the MANE v.0.7 curated transcript list, providing that all splicing junctions for a given transcript were supported in the GENCODE v.19 annotation. Genes without MANE transcripts were assigned composite transcripts, consisting of the union of all junctions found in transcripts for the given gene in NCBI RefSeq. For genes lacking both a corresponding MANE and RefSeq transcript, the union of all junctions present in all GENCODE v.19-listed transcripts for that gene were used as the transcript model.

To investigate the suitability of our hierarchical transcript selection approach and the stability of MRSD scores across transcripts, we also generated MRSD scores for all transcripts listed in the GENCODE v.19 annotation, using default MRSD parameters. MRSD scores for transcripts selected through the hierarchical approach were stratified according to whether they were classified as unfeasible or feasible and compared against the transcript-level MRSD predictions for all transcripts available in GENCODE for the given gene.

### Ethics approval and consent to participate

External datasets utilized in this study were accessed under dbGaP project accessions phs000655.v3.p1.c1 and phs000424.v8.p2. Informed written consent was obtained for all in-house analyses, with ethical and study approval from South Central-Hampshire A (ref: 17/SC/0026), South Central-Oxford B (ref:11/SC/0269), South Manchester (ref:11/H10003/3), and Scotland A (refs: 06/MRE00/76 and 16/SS/0201) Research Ethics Committees.

### Reference set generation from control RNA-seq data

FASTQs were downloaded from the Database of Genotypes and Phenotypes (dbGaP) under the project accessions phs000424.v8.p2 and phs000655.v3.p1.c1 for GTEx control individuals and neuromuscular disease-affected individuals, respectively. GTEx controls were selected for LCLs (n = 91), skeletal muscle (n = 184), whole blood (n = 150), and cultured fibroblasts (n = 150) according to tissue-specific criteria (supplemental material and methods S3) to ensure use of only high-quality samples in generating control splicing datasets. A collated map of splice junction coverage was generated for our defined transcripts (see Hierarchical approach to transcript selection) from these control datasets using established methods.^13^ These samples and their associated splice junction usage were designated as reference sets*.*

### In-house RNA-seq generation

We evaluated the accuracy of MRSD using independently derived RNA-seq samples from the reference sets which generated the model. The positive predictive value (PPV) was defined as the proportion of transcripts where the obtained sequencing depth for splicing junctions exceeded or equaled the MRSD prediction. Conversely, the negative predictive value (NPV) was defined as the proportion of transcripts where appropriate sequencing coverage was not obtained according to the MRSD parameters applied.

The RNA-seq datasets utilized in these analyses were accessed from previously published datasets^13^ (dbGaP project accession phs000655.v3.p1.c1), through international consortia,^19^ or from individuals in whom written informed consent was obtained and ethical approval for the study granted by Scotland A (refs: 06/MRE00/76 and 16/SS/0201), South Central-Hampshire A (ref: 17/SC/0026), South Central-Oxford B (ref:11/SC/0269), or South Manchester (ref: 11/H10003/3) Research Ethics Committee.

For in-house peripheral blood samples, RNA was extracted from PAXgene Blood RNA Kits and underwent poly-A enrichment library preparation using the TruSeq Stranded mRNA assay (Illumina) followed by 76 bp paired end sequencing using an Illumina HiSeq 4000 sequencing platform. For in-house LCL samples, RNA was extracted from pelleted LCLs thawed directly into TRIzol reagent (Invitrogen, 15596-026) using chloroform and treated with TURBO DNase (Invitrogen, AM1907), following the manufacturers’ instructions. RNA was prepared using the NEBNEXT Ultra II Directional RNA Library Prep kit (NEB #7760) with the Poly-A mRNA magnetic isolation module (NEB #E7490), according to manufacturer’s instructions, and 75 bp paired end sequencing was performed using the Illumina NextSeq 550 sequencing platform. Ribosomal RNA-depleted datasets were generated using RNA extracted via the PAXgene Blood RNA system, and 150 bp paired end sequencing performed via Novogene (Hong Kong) using the NEBNext Globin and rRNA Depletion and NEBNext Ultra Directional RNA Library Prep Kits on a HiSeq 2000 instrument (Illumina). RNA samples from 20 LCLs were obtained from the kConFab consortium. Poly(A)-selected RNA was generated using the TruSeq Stranded mRNA Library Prep Kit (Illumina), and 150 bp paired end reads created using the NextSeq 500 instrument (Illumina).

### Splice event identification

All FASTQs were aligned and processed as previously described.^13^ Briefly, this analysis consisted of two-pass alignment using STAR^20^ (v.2.4.2), marking of suspected PCR duplicates, and processing of the resulting alignments to generate tissue-by-tissue lists of read support counts for splice junctions present within the samples in the cohort. Metrics for each splicing event were collected (Box 1), and splicing junctions were filtered to retain only those events that were unique to single samples (singletons) or that were present in multiple samples (non-singletons) but with an increased usage in the sample of interest, i.e., a higher normalized read count (NRC) than any control in the reference set. The resulting list of splice events was ranked according to NRC fold change, with singletons with high read counts considered the most significant events.

Box 1 **Metrics collated during splice event analysis**

**Read count:** Number of split reads supporting the existence of a given splice junction
**Normalized read count (NRC):** Ratio of the number of reads supporting a given junction to the numbers of reads supporting adjoining canonical junction with the highest supporting read count
**NRC fold change:** fold difference in NRC for a given event between an individual and the control individual with the next-highest NRC for that event
**Number of samples:** the number of individuals, across both case and controls, in which an event is present
**Rank:** position of a given event in a list of significant events, when ordered by decreasing read count (for singleton events) or fold change (for non-singleton events)

### Factors influencing the likelihood of aberrant splicing identification

To calculate how the level of background splicing aberrations was altered by sample size, each individual in three of the four reference sets was processed using the above pipeline^13^ and compared against 2,000 bootstraps of 30, 60, and 90 control subjects each from their respective control tissue dataset with replacement. Events were then filtered to retain only those events for which the NRC was higher in the given individual than in any controls. Median counts for singleton and non-singleton events were collated for each control group size.

To understand the impact of splicing junction coverage on the ability to retain events of interest, we selected 31 splicing events identified in neuromuscular patient RNA-seq data that were either unique to or had increased NRC in comparison to the tissue-specific reference set. For these individuals, we removed random subsets of reads in 10% intervals from each of the genes containing these events. The resulting datasets mimicked variable expression of a single gene in these samples and were subsequently analyzed using the splice analysis pipeline.^13^

### Genomics England PanelApp data collection

Tabulated versions of 295 gene panels were downloaded from the Genomics England PanelApp repository on June 28, 2021. Each panel was filtered to retain only multiexon genes assigned a “green” classification, representing the highest level of confidence of a real genotype-phenotype association. This yielded 3,322 unique genes for downstream analysis.

### Curation of ClinVar variants of uncertain significance

A tabulated version of the comprehensive ClinVar variant listing^21^ for January 2021 was downloaded and filtered to retain only those variants that were annotated as either “uncertain significance” or “conflicting interpretations of pathogenicity.” SpliceAI scores^22^ (v.1.2.1) were generated for these variants and those with a score of 0.5 or greater retained for downstream analysis.

## Results

### Minimum required sequencing depth (MRSD) scores differ across biosamples

We curated a list of 3,322 multi-exon disease-related genes and defined a single transcript for each gene using our hierarchical approach (see material and methods). MRSD scores were generated for these transcripts using GTEx samples for four clinically relevant tissues to create tissue-specific reference sets (Figure S2): whole blood (n = 150), LCLs (n = 91), skeletal muscle (n = 184), and cultured fibroblasts (n = 150). MRSD scores for these reference sets are available (see web resources).

**Figure 1:**
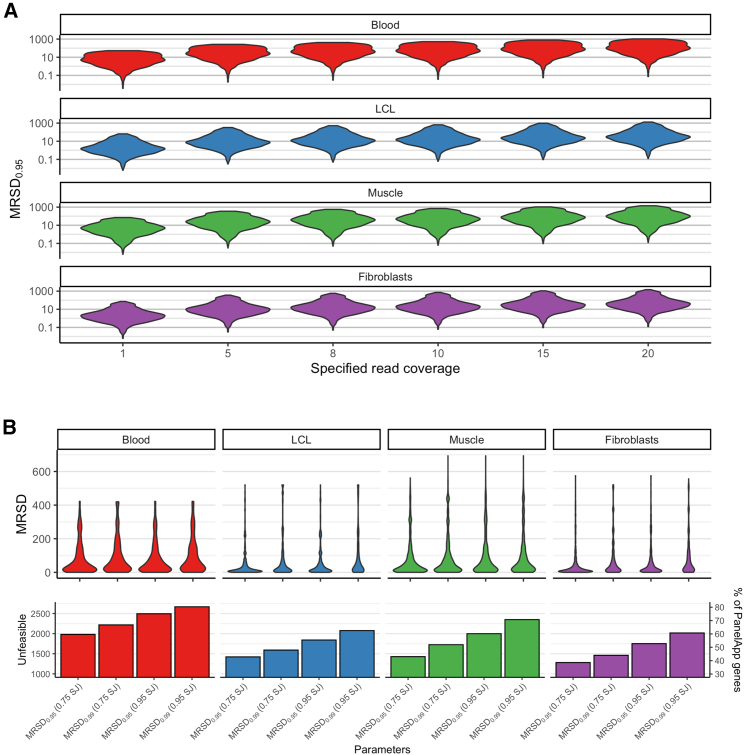
Minimum required sequencing depth (MRSD) predictions vary with changes in model parameters and across tissues (A) When all other parameters are constant (default parameters used here), increasing the desired level of read coverage of a gene results in a proportional increase in MRSD. (B) Top: In most cases, for a given level of splice junction (SJ) coverage, increasing the desired MRSD parameter (the proportion of RNA-seq runs for which the MRSD prediction is expected to be sufficient) results in an increase in median MRSD score. Bottom: The number of genes predicted to be unfeasible for analysis increases gradually as parameter stringency increases. At the highest level of stringency, the specified coverage was predicted unfeasible for between 62.5% (2,076/3,322, in LCLs) and 80.3% (2,668/3,322, in blood) of PanelApp genes.

Three parameters can be altered for the MRSD model (desired read coverage, percentage of splice junctions, and the MRSD parameter). We observed that the MRSD score differed dependent on the values chosen for these parameters (Figure 1). For example, when specifying a desired read coverage level of eight reads per splicing junction, we observed that increases in the desired proportion of covered splice junctions from 75% to 95% was associated with an increase in median MRSD of between 0.27% (in skeletal muscle, MRSD_0.99_) and 55.95% (in LCLs, MRSD_0.95_; Figure 1B, top). For all but one parameter combination, moving from MRSD_0.95_ to MRSD_0.99_ resulted in an increase in median MRSD of between 26.19% and 155.40% (Figure 1; supplemental results).

Overall, our analyses suggested that, of the four investigated biosamples, fibroblasts enable investigation of the most comprehensive set of genes for aberrant splicing. Although LCLs displayed the lowest median MRSDs across all parameter combinations (range = 12.86–33.77, Figure 1B, top), the difference in median MRSDs compared to fibroblasts was small (range = 14.44–35.06) and a greater number of genes were predicted “unfeasible” for analysis (see material and methods) in LCLs than in fibroblasts (42.8%–62.5% versus 38.6%–60.7% of PanelApp genes, respectively). Whole blood exhibited the highest number of unfeasible genes across the different parameter combinations (59.7%–80.3%).

### Accuracy of minimum required sequencing depth (MRSD) calculations

**Figure 2:**
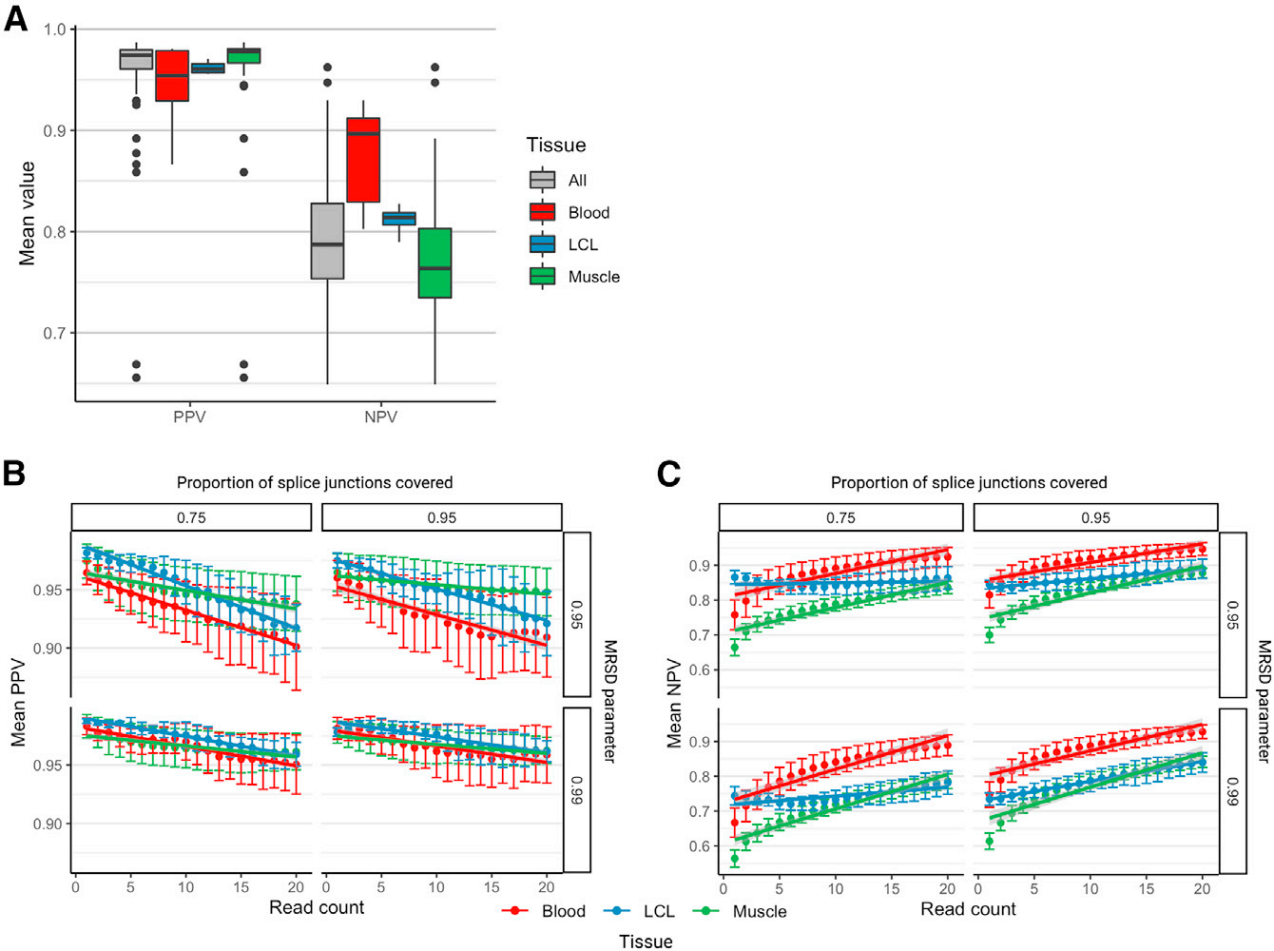
Performance metrics of the MRSD model The ability of MRSD to accurately predict levels of PanelApp disease gene coverage based on sequencing depth was tested on unseen RNA-seq datasets from blood (n = 12), LCLs (n = 4), and muscle (n = 52). (A) The mean positive predictive values (PPVs) and negative predictive values (NPVs) averaged across all parameter combinations for each RNA-seq dataset show that the median PPV is slightly lower, and the median NPV slightly higher, for whole blood than for LCLs and skeletal muscle. (B and C) Breakdown of (B) PPVs and (C) NPVs for the MRSD model by parameters shows that specifying an increasing desired read coverage results in a gradual decrease in PPV and increase in NPV across all tissues and parameter combinations. Dependent on parameter stringency and limiting analysis to a maximum specification of 20-read coverage, PPV predictions range from 90.1% to 98.2%, while NPV ranges from 56.4% to 94.7%. Error bars show 95% confidence interval.

In order to assess the performance of the MRSD model across a variety of parameter combinations, we obtained independent RNA-seq datasets for 68 samples for three of the four investigated tissues (blood, n = 12; LCLs, n = 4; muscle, n = 52), with a wide range of sequencing depths (Figure S3). All data utilized in this analysis were generated through 75 bp paired end sequencing. We observed 96% PPV and 79% NPV, on average, for the 68 samples (Figure 2A). We observed a general trend that the PPV and NPV of MRSD decreased and increased, respectively, at higher levels of required coverage (Figures 2B and 2C). Across all parameter combinations, PPVs ranged from 90.1% to 98.2%, while NPVs ranged from 56.4% to 94.7%, suggesting MRSD is a conservative model that primarily returns positive results with high certainty.

### Investigation of inter-transcript MRSD variability

We generated MRSD scores for all possible transcripts available in the GENCODE v.19 annotation (n = 20,188 genes with >1 transcript) and observed an overall median relative variability (coefficient of variation, CV_MRSD_) of 0.37–0.49 across the surveyed genes, depending on the tissue (Figure S4A). Where differences in MRSD predictions were observed, there was a median difference in MRSD of 1.06–3.65 M reads between our selected transcripts and the transcript with the lowest predicted MRSD for each gene (Figure S4B).

Further, in 95.10%–95.37%, of genes where automatically selected transcripts were classed as unfeasible, and in 89.05%–90.37% of multi-transcript genes classed as unfeasible, we observed that all transcripts in the GENCODE v19 dataset were also classified as unfeasible (Figure S5A). We observed an average minimum MRSD score of 108.59–157.78 M reads, dependent on tissue, for the small number of genes that displayed discordance in feasibility predictions between GENCODE v.19 and the automatically selected transcript (Figure S5B). These data illustrate a general trend of low variability in MRSD scores for genes with multiple possible transcripts, but importantly demonstrate that individual transcript selection may yield different MRSD scores in some contexts and thereby influence decisions on accessibility.

### Impact of read length on MRSD accuracy

To understand the impact of longer sequencing reads on MRSD accuracy, we evaluated the ability of the model to predict transcript coverage for independently derived 150 bp paired-end RNA-seq data (LCLs, n = 20). We observed higher median PPVs across samples for 150 bp datasets than with 75 bp datasets for half of the four parameter combinations tested (Figure S6). NPVs were slightly lower for 150 bp datasets for all combinations of parameters (Figure S6). While MRSD scores should ideally be applied to datasets generated using the same experimental approach, these data suggest that they are widely applicable to datasets generated through an alternative manner.

We also observed through a paired analysis of 150 bp and 75 bp datasets that 86.5% (1,559/1,802) of multi-exon disease genes that could be surveyed from LCLs either had lower MRSD scores from 150 bp read reference sets than from 75 bp read reference sets, or were only predicted to be feasible for surveillance from 150 bp reference sets (Figure S7; supplemental results). This further emphasizes the advantages of longer RNA-seq reads.

### Comparison of MRSD and TPM as a guide for appropriate surveillance

**Figure 3:**
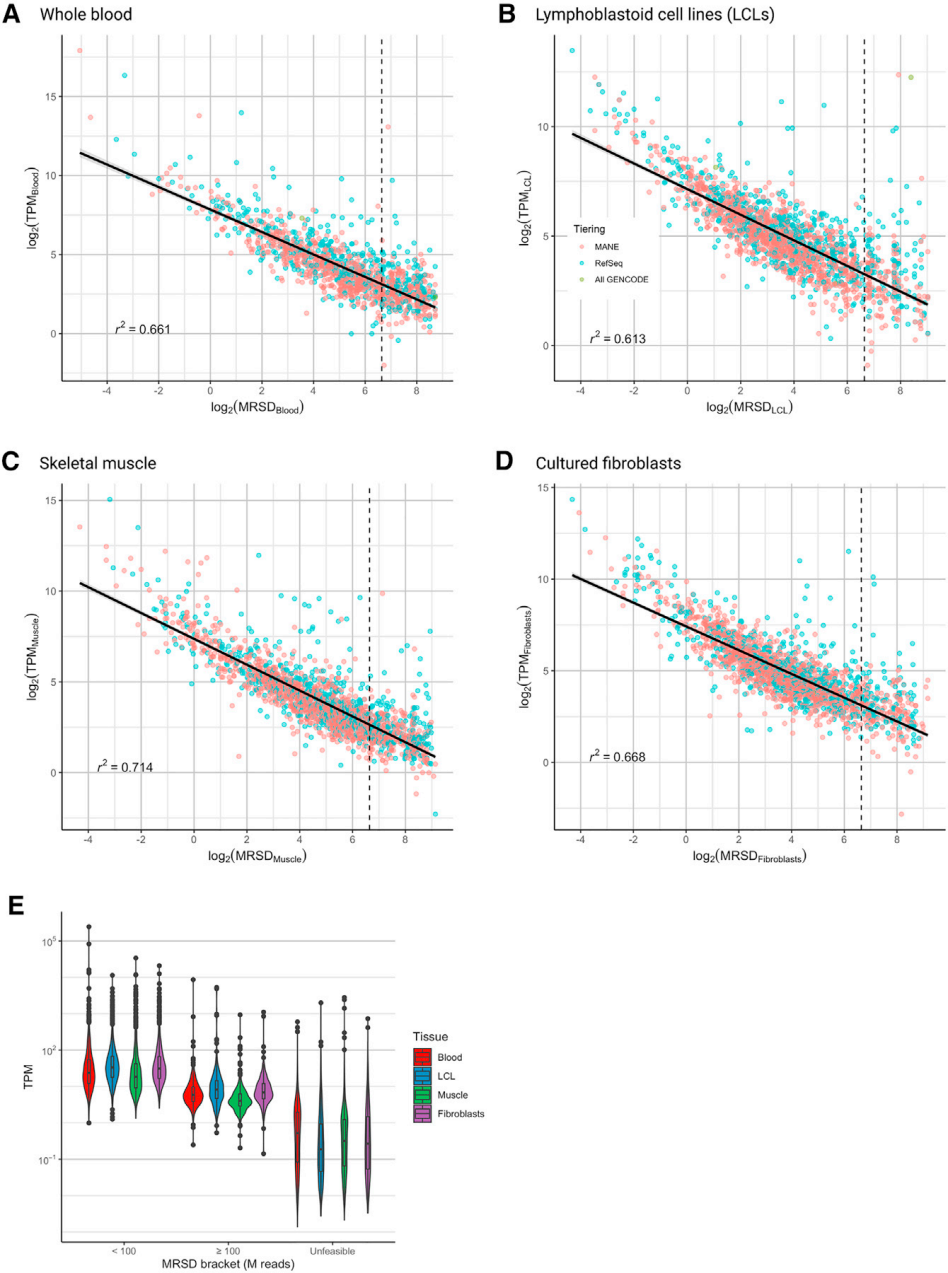
Comparison of MRSD and transcripts per million (TPM) predictions (A–D) MRSD and TPM predictions for 3322 multiexon genes present in the Genomics PanelApp repository are inversely correlated in (A) whole blood (*r*^2^ = 0.661), (B) LCLs (*r*^2^ = 0.613), (C) skeletal muscle (*r*^2^ = 0.714), and (D) cultured fibroblasts (*r*^2^ = 0.668). (E) Grouping PanelApp genes by MRSD range shows that there is substantial overlap in the TPMs of genes across different groups, suggesting relative expression level alone is not an adequate proxy for transcript coverage in some cases. Log transformation in (E) excludes 553 entries with TPMs of 0 in the unfeasible group. Default MRSD parameters (8-read coverage of 75% of splice junctions, MRSD_0.95_) used throughout.

We compared MRSD to the use of relative expression level (in transcripts per million, TPM) as a possible indicator of RNA-seq suitability for the detection of aberrant splicing events. We identified a negative correlation between the level of gene expression and its predicted MRSD across all four tissues (*r*^*2*^ = 0.613–0.714; Figures 3A–3D). This confirms that more highly expressed genes are associated with lower MRSD scores. However, we noted significant overlap between genes grouped into low-MRSD (<100 M reads) and high-MRSD (≥100 M reads) brackets (Figure 3D; supplemental results), suggesting that relative expression does not provide a wholly accurate representation of complete transcript coverage in RNA-seq data. Such inconsistencies may arise from bias in the regions of genes that are sequenced, for example, genes with high degrees of 3′ bias in RNA-seq datasets or significant alterations in isoform usage between tissues (Figure S8).

### Traits of pathogenic splicing variation vary widely between genes and events

We next aimed to determine the optimal MRSD parameters for detection of aberrant splicing through the investigation of 21 RNA-seq samples from patients harboring pathogenic mis-splicing events (Table S1; Figure S9). We observed high variability in indicative metrics associated with pathogenic aberrant splicing events using a previously published bioinformatics pipeline^13^ (Table 1). All pathogenic events identified through RNA-seq were supported by two or more reads and with normalized read counts (NRCs) ≥ 0.19. 90% of the known pathogenic events would be retained if filtering for events that were supported by 2 or more reads, and events that were singletons (evident only in a single sample) or non-singletons with an NRC > 0.25 (Table 1).

**Table 1:** Range of metrics observed for pathogenic splicing events

	**Tissue**		
**Metric**	**Whole blood (n = 3)**	**LCLs (n = 7)**	**Skeletal muscle (n = 11)**
Read count	2–40	4–38	2–462
NRC	0.48–1.25	0.19–1.52	0.34–3.19
NRC fold change	singletons	3.7–8.2 + singletons	19.6–442 + singletons
Number of samples	1	1–48	1–110
Rank	2-5	10–232	1–342
FRASER events identified	3/3	4/7	10/11
FRASER p values	7.97 × 10^−11^–0.0022	2.36 × 10^−5^–0.13182	4.27 × 10^−13^–0.0160
LeafCutterMD events identified	3/3	2/7	7/11
LeafCutterMD p values	6.19 × 10^−11^–0.00936	7.66 × 10^−6^–0.586	2.2 × 10^−15^–1.35 × 10^−3^
SPOT events identified	3/3	6/7	7/11
SPOT p values	0.000181–0.0426	1 × 10^−6^–0.13582	0.00469–0.0159

We also investigated the ability of three recent splice prediction tools to identify the 21 pathogenic mis-splicing events, specifically FRASER,^9^ SPOT,^17^ and LeafCutterMD.^18^ We observed variability in the events that were identified by these tools (Table 1). FRASER identified 81% (17/21) of pathogenic mis-splicing events, with 16 of these flagged as statistically significant splicing outliers (p < 0.05), including events supported by 3 or more sequencing reads.

### Factors influencing the likelihood of pathogenic splicing variation identification & MRSD predictions

**Figure 4:**
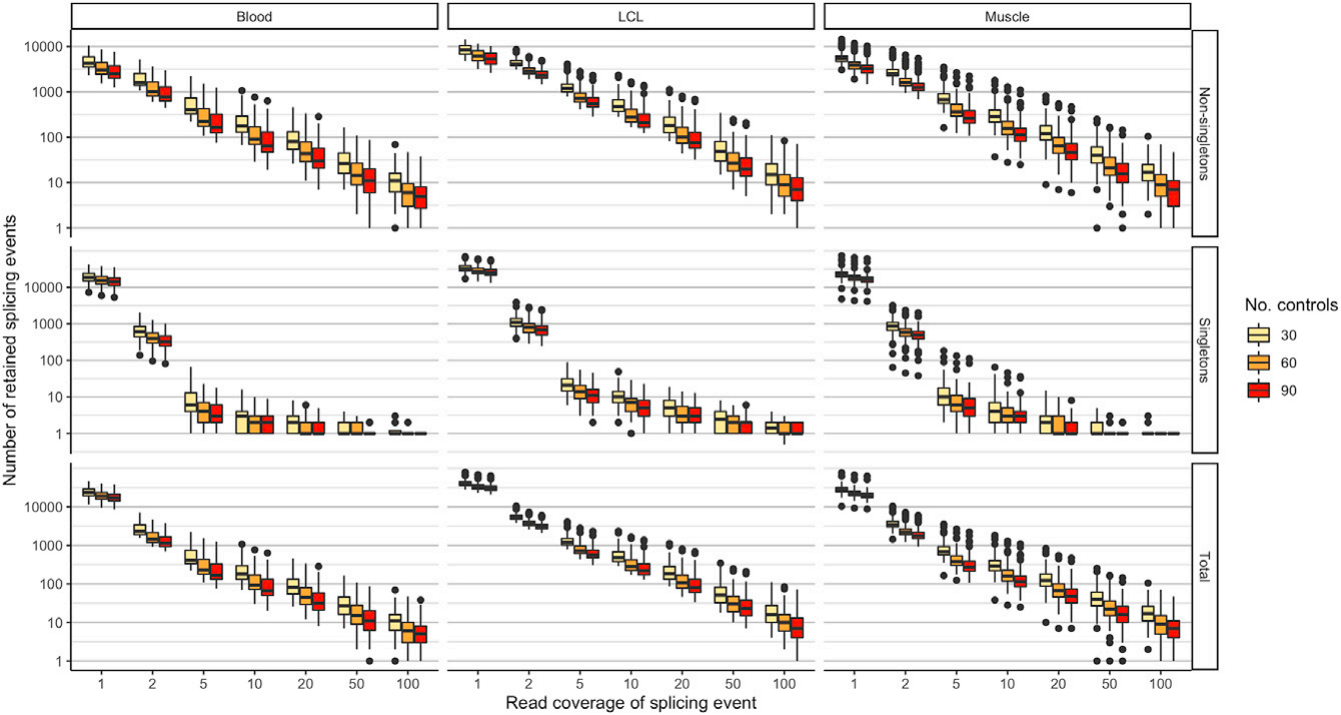
Expanding control datasets and enforcing read count thresholds improves filtering power when analyzing mis-splicing events There is a small decrease in the number of splicing events identified with increasing control size. Enforcing a read coverage threshold has a more significant effect on event counts, particularly for singleton events, where filtering out events supported by a single read removes up to 95% of singleton events. LCLs appear to exhibit the greatest number of splicing events regardless of read count filter, although this may be due to differences in sequencing depth between tissues. These data are generated from 2,000 bootstraps for control sizes of 30, 60, and 90 individuals. Outliers represent data points lying further than 1.5 times the interquartile range from the 25th and 75th percentile values.

**Figure 5:**
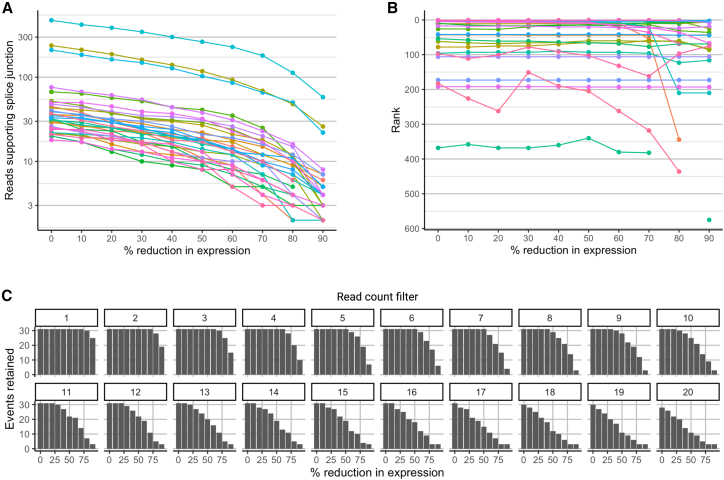
Variability in expression level influences the capacity to identify mis-splicing events Genes harboring a selection of 31 splicing events that were identified during analysis of 52 muscle-based RNA-seq datasets (and which would be identified as events of interest using a filter of normalized read count [NRC] > 0.19) were artificially downsampled to simulate variation in expression. (A) Reduction in expression leads to an intuitive and proportional reduction in the number of reads supporting each mis-splicing event. (B) The rank position of an event—where the event appears in a list of all splicing events in its respective sample, ordered by decreasing NRC fold change relative to controls, and placing singleton events above non-singletons—is generally consistent as expression of the gene decreases. Missing data points at the most reduced expression values are indicative of the splicing event not being identified by the applied bioinformatics pipeline. (C) Variation in expression impacts our ability to identify events of interest when filters of read count supporting the events are enforced. When the 31 events experience a 50% reduction in expression, for instance, the application of a minimum 15-read filter leads to the exclusion of 41.9% (13/31) of events.

We next investigated the impact of varying input metrics on the ability to successfully identify pathogenic splicing events. This includes number of samples within the reference set, degree of read support for splicing junctions, and relative expression of genes of interest (Figure S10). Overall, our analyses suggested that filtering for splicing junction supported by ≥2 reads reduces the number of identified events by up to 95% (Figure 4; supplemental results) and that mis-splicing events mostly retain their relative priority ranks at lower expression levels (Figure 5; supplemental results). Based on these investigations and our investigations for 21 known pathogenic splicing events (90% identified with ≥2 reads and NRC > 0.25, Table 1), we selected an 8 read minimum coverage value for downstream analyses.

### Implications for investigation of variants in known disease-causing genes

**Figure 6:**
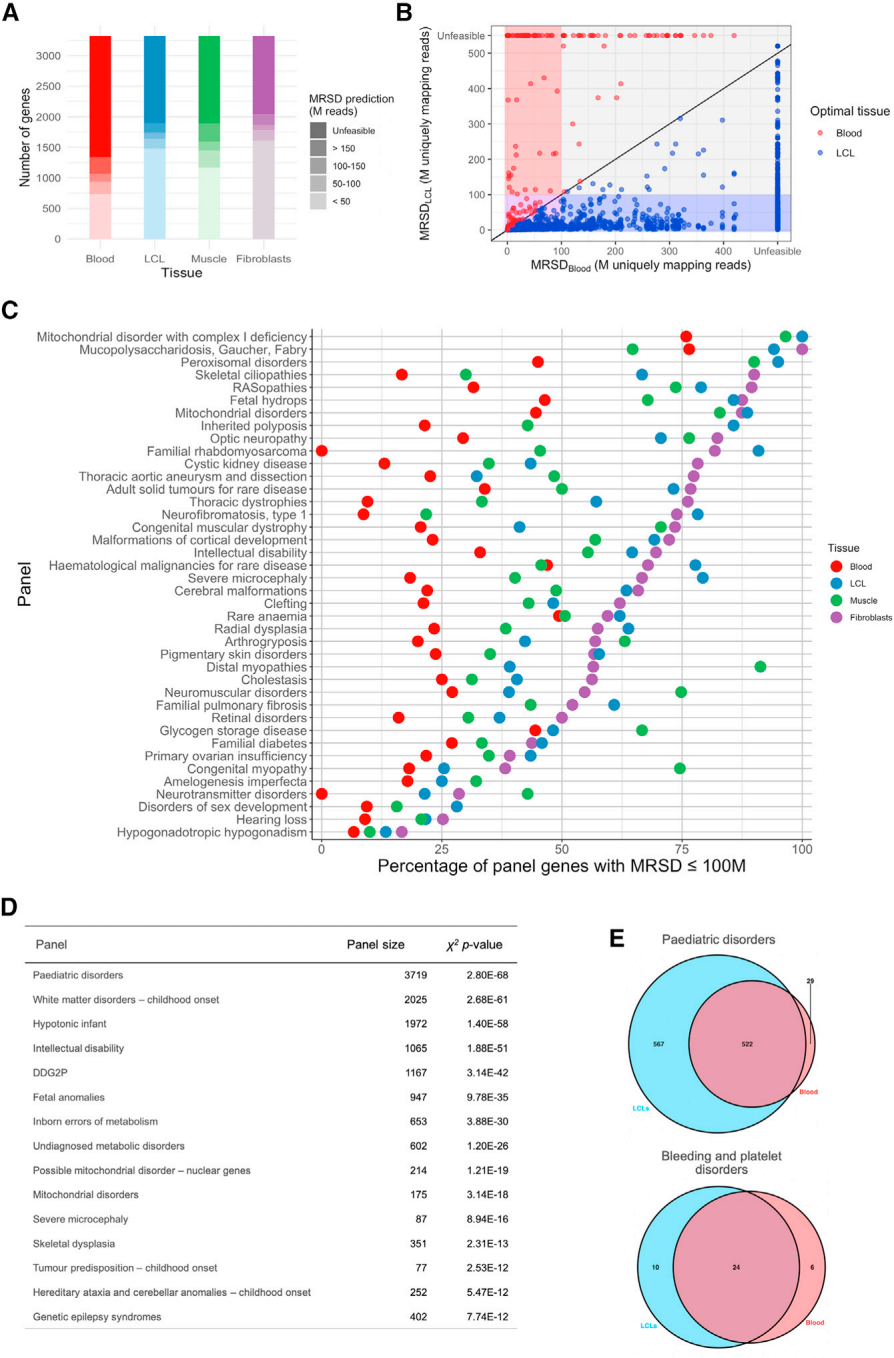
Application of MRSD scores to disease genes listed in the Genomics England PanelApp repository (A) Comparison of PanelApp panel gene MRSD predictions between tissues shows blood to exhibit markedly poorer coverage of disease genes than other tissues. (B) When comparing MRSD predictions for genes in blood and LCLs, 1,522 genes are considered “high-MRSD” (i.e., have an MRSD ≥ 100 M reads) in both tissues (gray). Genes which are exclusively low-MRSD (i.e., MRSD < 100 M) in blood are far fewer in number (with 66 genes, red box), while the remainder are low-MRSD in both (775 genes, purple box) or low-MRSD in LCLs only (749 genes, blue box). (C) Comparison of PanelApp panel gene MRSDs between tissues shows many panel genes have greater coverage in fibroblasts than blood and, to a lesser extent, LCLs and skeletal muscle over a variety of disease subtypes. 40 exemplar gene panels are shown here, see Figures S12 and S13 for all 295 PanelApp gene panels. (D) Top 10 panels with most significant difference between low- and high-MRSD gene counts between blood and LCLs (chi-square test). (E) Venn diagrams showing number of low-MRSD genes predicted in blood and LCLs for two exemplar disease gene panels.

We utilized MRSD scores for 3,322 multi-exon monogenic disease genes using standardized parameters (read coverage = 8; proportion of junctions = 75%; MRSD parameter = 95%). We acknowledge that these parameters may be too lenient for some use cases but expect trends to be similar across other applied MRSD parameter combinations (Figure 6). Using this approach, we observed that 64.2% (2,133/3,322) of PanelApp genes were predicted to be low-MRSD (<100 M reads required) in at least one of the four tissues (Figures 6A and S9). At the individual tissue level, 28.2% (936/3,322) of PanelApp genes in whole blood, 49.4% (1,641/3,322) in LCLs, 43.6% (1,447/3,322) in skeletal muscle, and 53.7% (1,784/3,322) in cultured fibroblasts were predicted to be low-MRSD (Figure 6A). Fibroblasts were observed to have the highest (or joint-highest) proportion of low-MRSD panel genes in 186/295 disease gene panels (63.1%, Figure 6C) compared to 126/295 panels for LCLs (42.7%), 70/295 panels (23.7%) for skeletal muscle, and 21/295 panels (7.1%) for whole blood (Figure S11).

MRSD predictions revealed many use cases for specific tissues: in the familial rhabdomyosarcoma panel, for example, none of the 11 genes were predicted to be low-MRSD in blood, while 10/11 were predicted low-MRSD in LCLs (Figure 6C), of which 9 were actually assigned an MRSD < 50 M reads. Results across all 295 panels are shown in Figures S12 and S13.

### Quantifying the resolving power of RNA-seq for variants of uncertain significance

**Figure 7:**
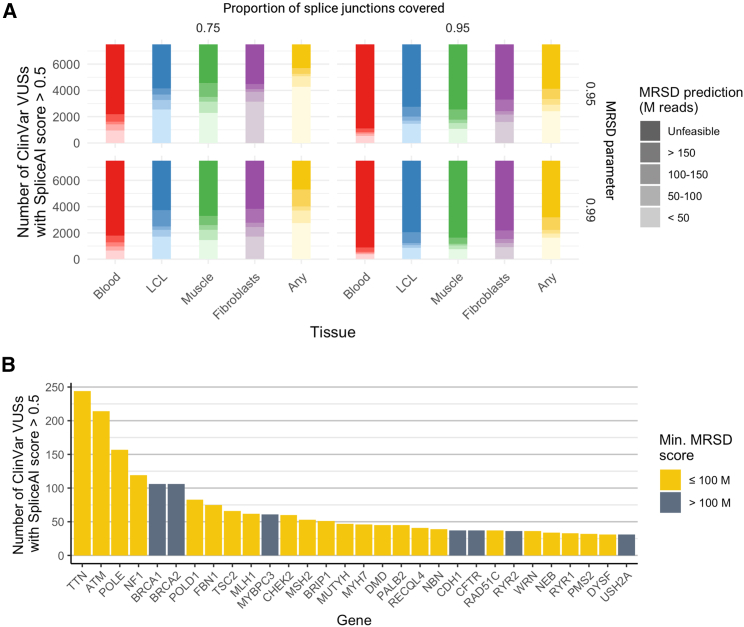
Quantifying the power for RNA-seq to resolve variants of uncertain significance (VUSs) MRSD scores were derived for genes harboring VUSs present in ClinVar if the variants were predicted by SpliceAI to impact splicing (score ≥ 0.5; Jaganathan et al.^22^). (A) Between 25.8% (1,940/7,507) and 67.8% (5,086/7,507) of variants predicted to impact splicing are expected to be adequately covered by 100 M uniquely mapping reads or fewer in at least one of the four tissues (whole blood, LCLs, skeletal muscle, and fibroblasts), dependent on model stringency. Variants were most likely to be found to be in low-MRSD genes (MRSD ≤ 100 M) in fibroblasts, irrespective of model parameters. (B) Among the 30 genes with the greatest number of predicted splice-impacting VUSs, 23 were predicted to be adequately covered (using default parameters) with 100 M uniquely mapping reads or fewer in at least one of the four tissues. An 8-read junction support parameter was used throughout.

To analyze the possible impact of RNA-seq integration on variant interpretation, we curated variants of uncertain significance (VUSs) from the ClinVar variant database^21^ that were predicted by SpliceAI^22^ to impact splicing (score ≥ 0.5; see material and methods). Of a total of 352,011 ClinVar variants, 185,119 (52.6%) were identified as VUS, and 7,507 (2.1%) were retained after filtering based on SpliceAI score. Cross-referencing the MRSDs of the transcripts harboring SpliceAI-prioritized variants across tissues revealed that, at a specified read coverage of 8 reads, between 25.8% and 67.8% of these variants may lie in genes that are low-MRSD in at least one of the four tissues (Figure 7A), dependent on the stringency of the model (Figure S14). Further, among the 30 genes in which the greatest number of predicted splice-impacting VUSs were identified, 76% (23/30) were predicted to be low-MRSD in at least one tissue (Figure 7B) at a desired read coverage of 8 reads. This is reduced to 73% (22/30) and 60% (18/30) of genes at desired read coverages of 10 and 20 reads, respectively.

## Discussion

Implementation of machine learning approaches has improved the ability to prioritize variants that impact splicing and cause rare disease.^23^ Despite these advances, corroboration of the effect of such variants remains a major obstacle. This is amplified by unexpected impacts that many variants may have on mRNA splicing.^6^

The MRSD-based approach that we describe here allows informed selection of biosample(s) for bulk RNA-seq, based on the required number of sequencing reads for appropriate surveillance of genes of interest. This enables effective patient-specific identification of genomic variants that are amenable for functional assessment of mis-splicing through RNA-seq. This can improve efficiency and accuracy of genomic diagnostic approaches. Although our model is conservative (Figure 2), we demonstrate through MRSD-guided re-inspection of VUSs in ClinVar that it may be possible to use RNA-seq to clarify the effect of >5,000 variants of uncertain significance (Figure 7A).

Other approaches to select genes amenable to functional analysis through RNA-seq include leveraging relative gene expression metrics^14^^,^^24^ or tools which assess the similarity of transcript isoforms between tissues, e.g., MAGIQ-CAT.^7^ We show that, while TPM values are well correlated with MRSD scores (Figures 3A–3C), uneven sequencing coverage across the length of the transcript may, in some cases, falsely identify specific genes or splice junctions as being amenable to RNA-seq-based analysis (Figure S8). 3′ sequencing bias, which is a known artifact of poly-A enriched mRNA sequencing,^25^, ^26^, ^27^ and alternative transcript usage across tissues may elevate the risk of inaccurately selecting genes that could be surveyed through RNA-seq when considering TPM alone. Additionally, the normalization against sequencing depth that occurs during the calculation of TPM obscures information about raw read count at the level of individual splicing junctions, which is important when analyzing the utility of RNA-seq for clinical diagnostics. MRSD scoring, conversely, leverages variation in sample read depth to provide quantitative predictions about optimal sequencing depths.

Other bioinformatics tools may complement the utility of MRSD; MAGIQ-CAT^7^ assesses the degree to which transcript isoforms in a sampled tissue accurately resemble those in the primary disease-affected tissue. However, MAGIQ-CAT primarily captures the degree of similarity between isoform structure and does not aim to provide a quantitative readout to guide biosample suitability. We envision that the use of both MAGIQ-CAT and MRSD could comprehensively capture information about the utility of RNA-seq, both in terms of similarity of isoform structure relative to the disease-affected tissue and in terms of the capability of observing disruptions to this structure at specific sequencing read depths. Future investigations of the stability of MRSD scores for tissue-specific and tissue-shared transcripts will be of interest.

There are limitations of the current MRSD model, which could be incorporated into future work. First, the MRSD model cannot directly be extended to predict the suitability of datasets to detect allele-specific expression biases and differential gene expression, which are known hallmarks of pathogenic mechanisms in known disease-causing genes.^10^^,^^11^^,^^14^^,^^28^ Although further investigations are required to quantify and prove this suitability, it is likely that genes with low MRSD scores (Figure 3D) are also amenable to investigations of differential gene expression and isoform imbalance.

Second, further extensions to the model could incorporate genomic background which influences gene expression profiles. For example, MRSD predictions may not accurately reflect the degree of sequencing coverage for certain transcripts in patients with disorders associated with widespread changes to the transcriptome, e.g., interferonopathies,^29^, ^30^, ^31^ chromatin structure disorders,^32^^,^^33^ and disruption of the spliceosome.^34^, ^35^, ^36^ Moreover, the current MRSD model does not explicitly account for the presence of expression quantitative trait loci (eQTLs) or splicing quantitative trait loci (sQTLs) which are known to influence gene expression profiles.^37^, ^38^, ^39^ We have demonstrated that modulation in expression levels may disrupt our ability to reliably highlight pathogenic splicing events (Figure 5C). As a greater number of paired transcriptome and genomic datasets become available, we expect that MRSD scores can be generated in a dynamic manner to account for the presence of eQTLs, sQTLs, other modifiers of gene expression profiles, and multiple testing issues that may arise from surveying multiple splice junctions and/or VUSs of interest for splicing aberrations through RNA-seq.

Third, our approach is built for a specific cohort of RNA-seq-based analyses; specifically, the analysis of a selection of tissues by bulk short-read poly-A enrichment RNA-seq processed using a specific bioinformatics analysis pipeline.^13^ This specific RNA-seq approach currently remains widespread;^13^, ^14^, ^15^ the behavior of MRSD scores for other experimental and/or bioinformatics approaches will be an interesting avenue for further research. However, our data suggest that the MRSD model may be readily applicable to RNA-seq generated using alternative methodologies, such as increased read length, with only minor variations in model performance (Figure S6). As other technologies, such as long-read,^40^, ^41^, ^42^ single-cell,^43^^,^^44^ and spatially resolved RNA-seq,^45^, ^46^, ^47^, ^48^ become more prevalent in a clinical setting, appropriate control datasets must be generated to develop corresponding MRSD models. Similarly, recent research has shown noticeable improvements to diagnostic yield for neuromuscular disorders by conducting RNA-seq on *in vitro* myofibrils generated by a fibroblast-to-myofibril transdifferentiation protocol.^49^ Such patient-derived cell line approaches represent a promising avenue to scrutinize transcripts not otherwise observable in proxy tissues.^35^^,^^50^ As these protocols gain wider use, generation of control RNA-seq data from healthy individuals using these approaches will be vital both to allow the generation of MRSD scores and to accurately assess pathogenicity of any identified mis-splicing events.

In summary, the MRSD model presented here offers a gene-specific readout to predict the most suitable biosample for interrogation of splicing disruption at the transcript level. This may uncover previously unintuitive choices of biosample, as discussed above in the case of familial rhabdomyosarcoma (Figure 6C). We expect that the use of MRSD will allow effective and appropriate integration of RNA-seq into diagnostic genomic services and ultimately improve variant interpretation and diagnostic yield.

## Consortia

The members of the kConFab Investigators are David Amor, Lesley Andrews, Yoland Antill, Rosemary Balleine, Jonathan Beesley, Ian Bennett, Michael Bogwitz, Leon Botes, Meagan Brennan, Melissa Brown, Michael Buckley, Jo Burke, Phyllis Butow, Liz Caldon, Ian Campbell, Deepa Chauhan, Manisha Chauhan, Georgia Chenevix-Trench, Alice Christian, Paul Cohen, Alison Colley, Ashley Crook, James Cui, Margaret Cummings, Sarah-Jane Dawson, Anna DeFazio, Martin Delatycki, Rebecca Dickson, Joanne Dixon, Ted Edkins, Stacey Edwards, Gelareh Farshid, Andrew Fellows, Georgina Fenton, Michael Field, James Flanagan, Peter Fong, Laura Forrest, Stephen Fox, Juliet French, Michael Friedlander, Clara Gaff, Mike Gattas, Peter George, Sian Greening, Marion Harris, Stewart Hart, Nick Hayward, John Hopper, Cass Hoskins, Clare Hunt, Paul James, Mark Jenkins, Alexa Kidd, Judy Kirk, Jessica Koehler, James Kollias, Sunil Lakhani, Mitchell Lawrence, Geoff Lindeman, Lara Lipton, Liz Lobb, Graham Mann, Deborah Marsh, Sue Anne McLachlan, Bettina Meiser, Roger Milne, Sophie Nightingale, Shona O'Connell, Sarah O'Sullivan, David Gallego Ortega, Nick Pachter, Briony Patterson, Amy Pearn, Kelly Phillips, Ellen Pieper, Edwina Rickard, Bridget Robinson, Mona Saleh, Elizabeth Salisbury, Christobel Saunders, Jodi Saunus, Rodney Scott, Clare Scott, Adrienne Sexton, Andrew Shelling, Peter Simpson, Melissa Southey, Amanda Spurdle, Jessica Taylor, Renea Taylor, Heather Thorne, Alison Trainer, Kathy Tucker, Jane Visvader, Logan Walker, Rachael Williams, Ingrid Winship, and Mary Ann Young.

## Data and code availability

The control datasets used to generate the MRSD model are available through the dbGaP repository (see web resources) under controlled access through the GTEx v8 data release (data used in this study was accessed through accession ID: phs000424.v8.p2). Muscle-derived RNA-seq datasets to test the MRSD model are available through dbGaP (accessed in this study through accession ID: phs000655.v3.p1.c1). Source code for MRSD calculation and pre-computed MRSD scores for all GENCODE v.19 genes across the four investigated tissues are available (see web resources). MRSD resources are made freely available without access control.

## Web resources

dbGaP, https://www.ncbi.nlm.nih.gov/gap/
MRSD web portal, https://mcgm-mrsd.github.io/

## Supplemental information

Document S1. Figures S1–S15, Tables S1 and S2, supplemental material and methods, and supplemental results

Document S2. Article plus supplemental information
