# Supplementary material for: MRSD: A quantitative approach for assessing suitability of RNA-seq in the investigation of mis-splicing in Mendelian disease
Source: Am J Hum Genet. 2022 Jan 21;109(2):210–22. doi: 10.1016/j.ajhg.2021.12.014 (PMC8874219; doi:10.1016/j.ajhg.2021.12.014)
Supplement: Document S1. Figures S1–S15, Tables S1 and S2, supplemental material and methods, and supplemental results [file mmc1.pdf]

The American Journal of Human Genetics, Volume *109*

Supplemental information

MRSD: A quantitative approach for assessing suitability of RNA-seq in the investigation of mis-splicing in Mendelian disease

Charlie F. Rowlands, Algy Taylor, Gillian Rice, Nicola Whiffin, Hildegard Nikki Hall, William G. Newman, Graeme C.M. Black, kConFab Investigators, Raymond T. O'Keefe, Simon Hubbard, Andrew G.L. Douglas, Diana Baralle, Tracy A. Briggs, and Jamie M. Ellingford

List of Contents

Figure S1
Categories of potentially pathogenic splicing events and their representation in analytical pipeline output

Figure S2
Workflow for MRSD score generation

Figure S3
Sequencing depths of RNA-seq samples used for evaluation of MRSD model accuracy

Figure S4
MRSD scores vary among the different transcripts of individual genes

Figure S5
Transcripts deemed as unfeasible through hierarchical selection are themselves likely to be unfeasible

Figure S6
Effect of varying sequencing read length on MRSD model performance

Figure S7
MRSD scores are generally lower when derived from RNA-seq runs of longer read length

Figure S8
Evidence for 3' sequencing bias confounding the use of TPM as a guiding RNA-seq metric

Figure S9
Exemplar events identified during pathogenic splice event analysis

Figure S10
Relative gene expression level does not reflect the raw read coverage of transcript splice junctions

Figure S11
Pairwise comparisons, by tissue, of MRSD scores for PanelApp disease gene

Figure S12
Proportion of low-MRSD genes per tissue for all PanelApp panels, ordered by panel size

- **a) Low-MRSD gene proportions for large panels (> 50 genes)**
- **b) Low-MRSD gene proportions for medium panels (21-50 genes)**
- **c) Low-MRSD gene proportions for small panels (11-20 genes)**
- **d) Low-MRSD gene proportions for very small panels (≤ 10 genes)**

Figure S13
Proportion of low-MRSD genes per tissue for all PanelApp panels, ordered alphabetically by panel name

- **a) Low-MRSD gene proportions for panels named A-E**
- **b) Low-MRSD gene proportions for panels named F-L**
- **c) Low-MRSD gene proportions for panels named M-R**
- **d) Low-MRSD gene proportions for panels named S-X**

Figure S14
Increasing specified read coverage reduces the number of ClinVar variants that can be analyzed

Figure S15
Increasing specified read count removes highly VUS-prone genes from the scope of analysis

Table S1
Summary of pathogenic splicing variants analyzed during this study

Table S2
Summary of datasets used in this study

Methods S1
Illustration of MRSD calculation methodology

Methods S2
Tiering methodology for selection of transcripts for MRSD generation

Methods S3
Tissue-specific criteria for filtering of high-quality GTEx control RNA-seq datasets

Methods S4
Sample IDs of GTEx samples used to generate control datasets

- **Skeletal muscle**
- **Whole blood**
- **EBV-transformed lymphocytes (LCLs)**
- **Cultured fibroblasts**

Supplementary Results

References

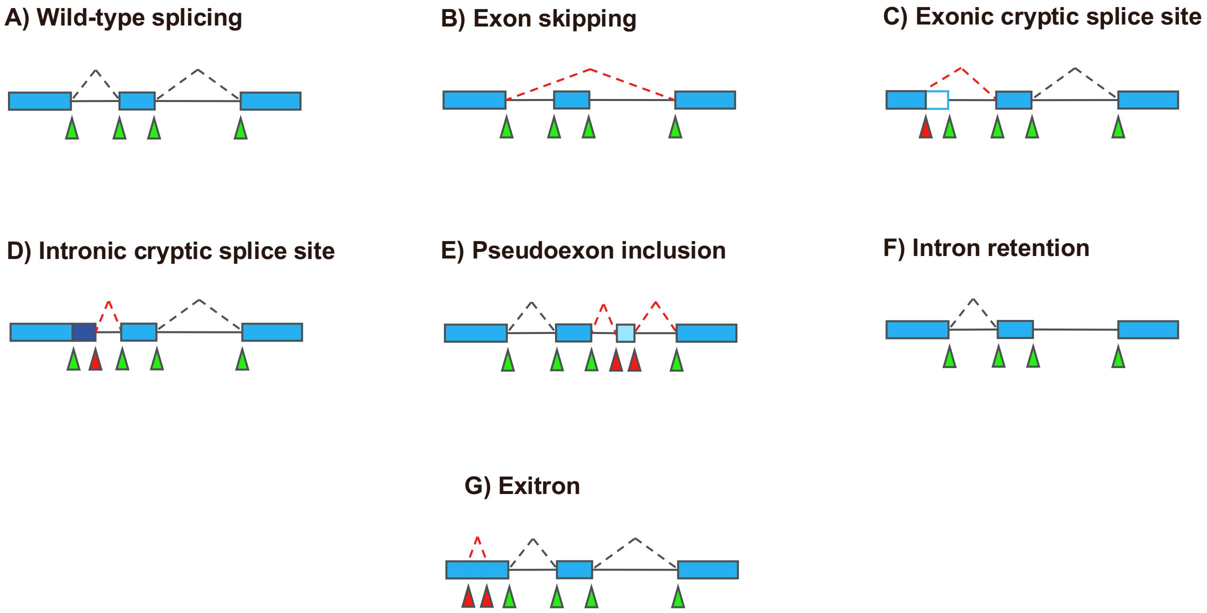

Figure S1. *Categories of potentially pathogenic splicing events and their representation in analytical pipeline output.* Disruption of (**A**) wild-type splicing may lead to (**B**) skipping of one or more exons, the creation of novel splice sites in (**C**) exonic or (**D**) intronic regions that may outcompete the canonical sites, or result in (**E**) the generation of an intronic pseudoexon. (**F**) Splicing may be abrogated completely, leading to total retention of the intron. (**G**) Within longer exons, creation of a novel splice site may lead to a so-called "exitron", whereby a central portion of the exon is absent from the final transcript. Green triangles indicate canonical splice sites; red triangles indicate non-canonical sites.

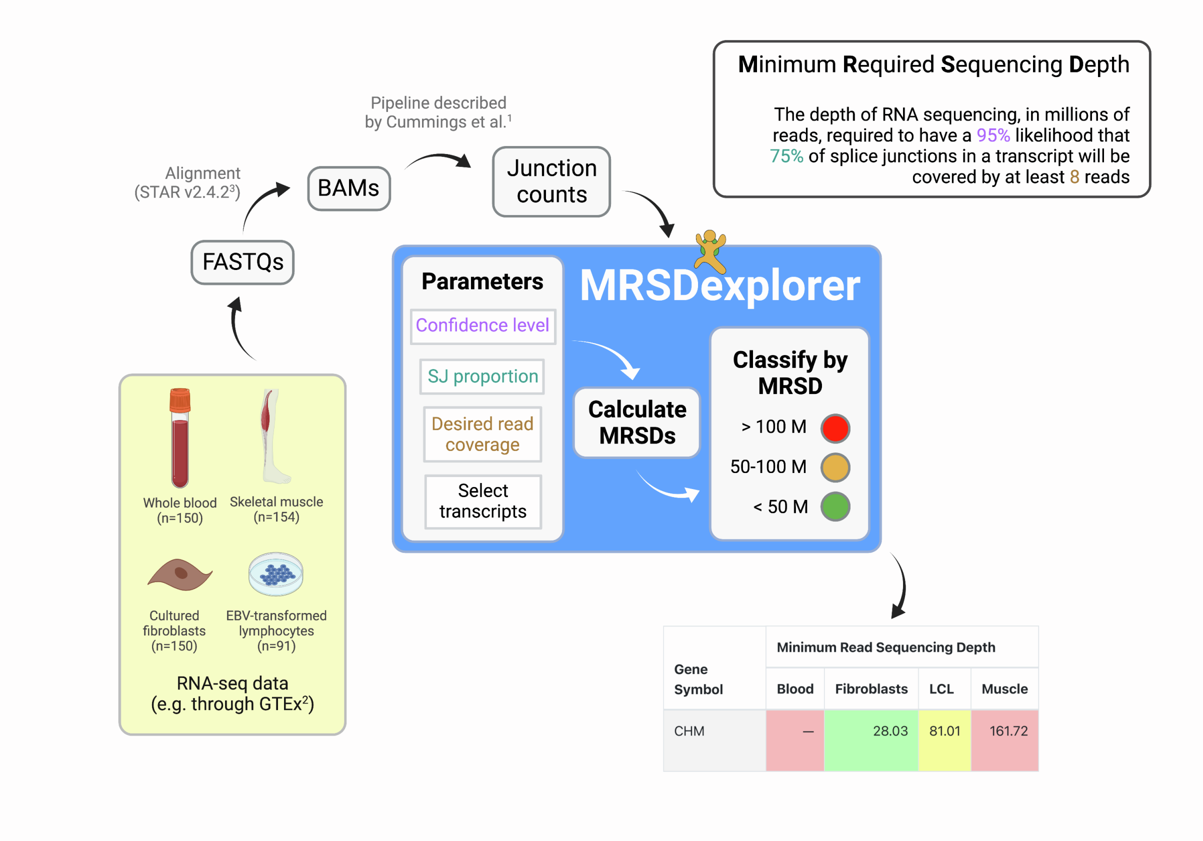

Figure S2. *Workflow for MRSD score generation.* Users can create their own MRSD scores using the code provided online at https://github.com/mcgm-mrsd/mrsd-explorer. Starting with a set of RNA-seq samples, reads are aligned and the split reads counted using an established pipeline. Then, using our bespoke Python scripts, users can generate their own predictive scores (using parameters of their choice) and classify transcripts according to the level of sequencing required to obtain the specified coverage. Alternatively, users are free to investigate pre-computed scores for all GENCODE v19 genes across four tissues (whole blood, skeletal muscle, cultured fibroblasts and lymphoblastoid cell lines, or LCLs) at our web portal: http://mcgm-mrsd.github.io/

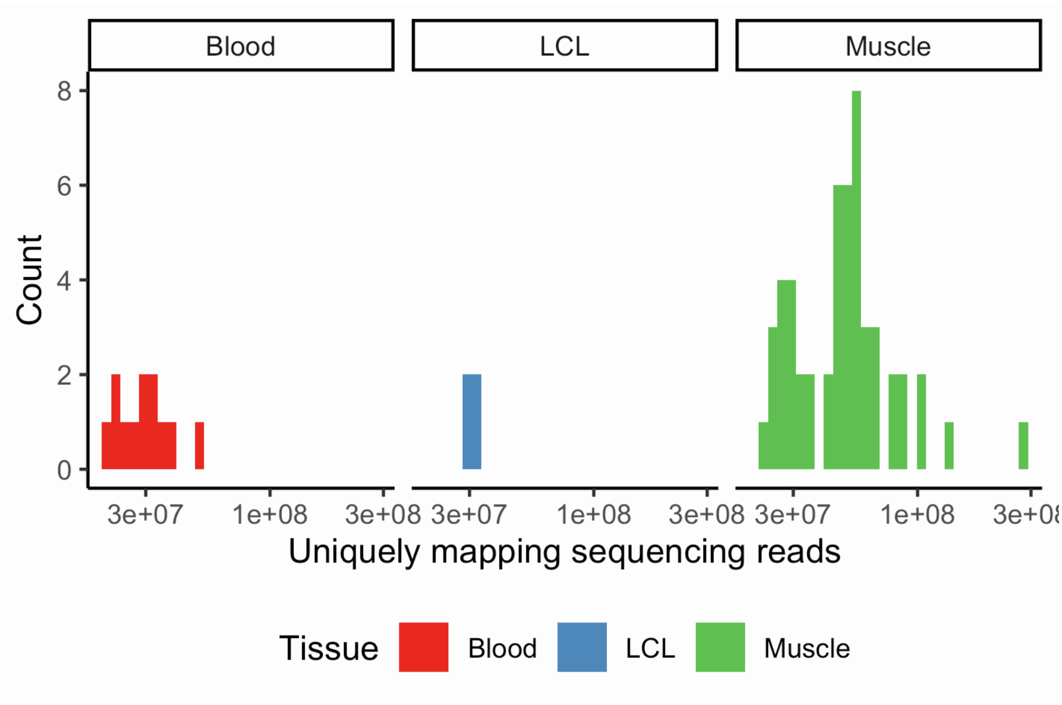

Figure S3. *Sequencing depths of RNA-seq samples used for evaluation of MRSD model accuracy.* Whole blood (*n* = 12), LCL (*n* = 4) and skeletal muscle (*n* = 52) RNA-seq samples were derived from in-house or previously published data (3) for validation of the MRSD model efficacy. Sequencing depths across the three tissues ranged from 20.6-281.5 M uniquely mapping reads.

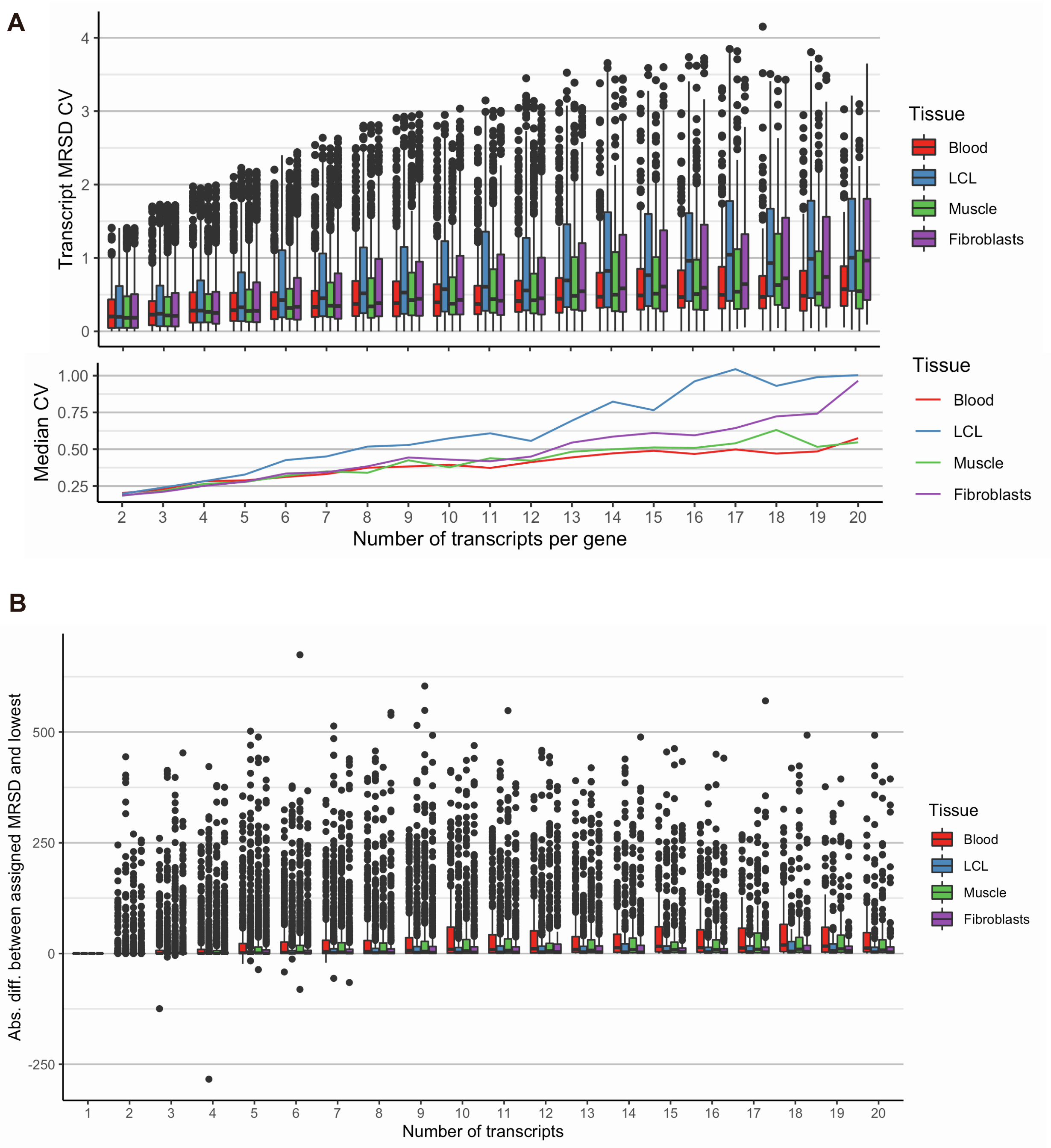

Figure S4. *Extent of variability in MRSD scores among the different transcripts of individual genes.* (**A**) Considering the MRSDs of genes with up to 20 MRSD-feasible GENCODE-annotated transcripts, we observed a median relative variability in MRSD (coefficient of variation, CV) across the four analysed tissues of 0.37-0.49. An increased number of transcripts per gene was associated with a small, gradual increase in CV. (**B**) Where our selected transcript generated an MRSD prediction, we observed only a small median difference in MRSD between this prediction and that of the lowest-MRSD transcript annotated for the same gene (median difference of 1.06-3.65 M reads).

A

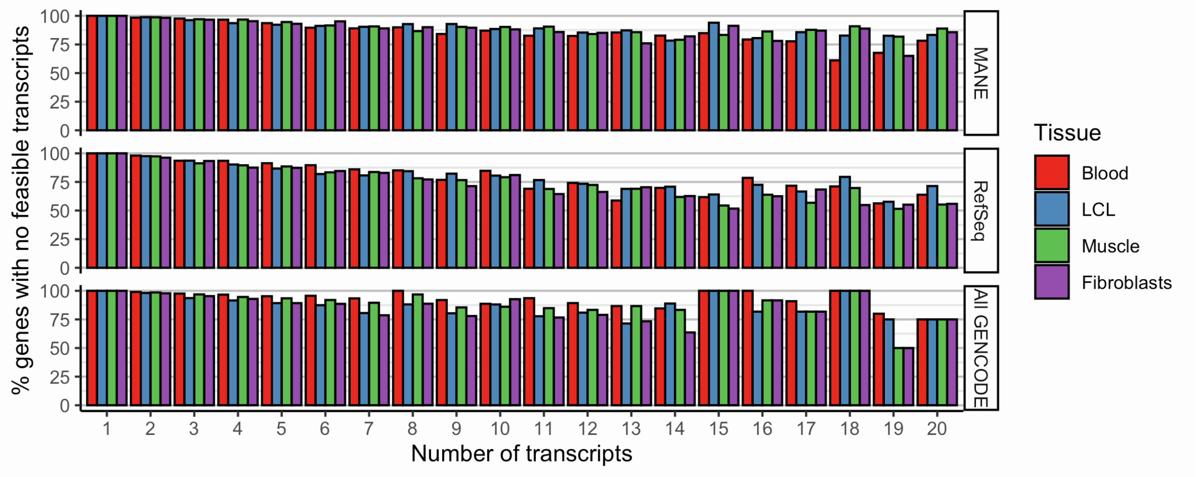

B

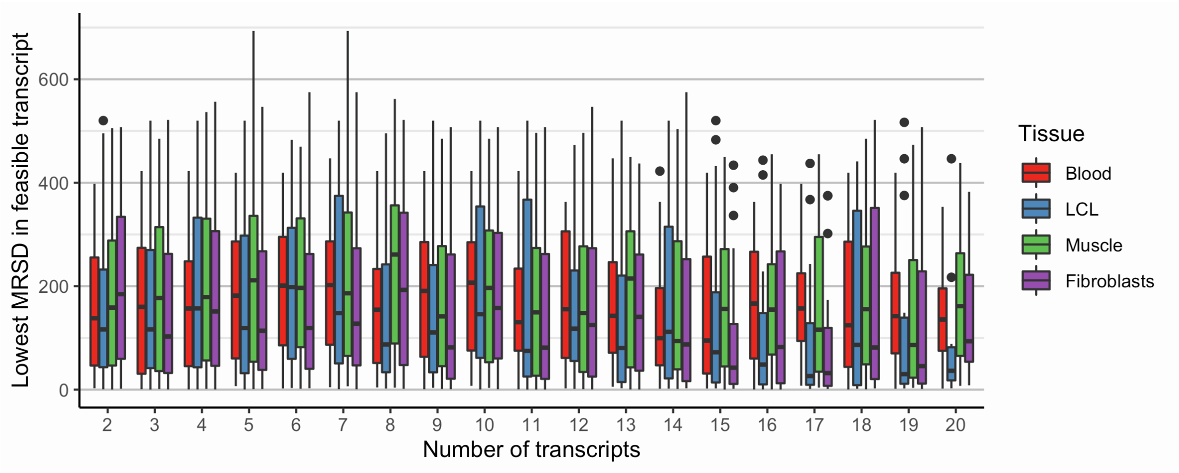

Figure S5. *Transcripts in genes deemed unfeasible through hierarchical selection are themselves likely to be unfeasible.* (**A**) Among genes for which our hierarchically selected transcript is deemed unfeasible through MRSD, 89.05-90.37% with multiple transcripts in GENCODE v19 are predicted to have no feasible transcripts. Of all the transcript tiers, unfeasible RefSeq composite transcripts are most likely to be assigned to genes with at least one feasible transcript. (**B**) In the remaining cases (in which an unfeasible gene is predicted to have at least one feasible transcript), the median MRSD for the lowest-MRSD transcript ranges from 108.59-157.78 M reads, depending on tissue choice.

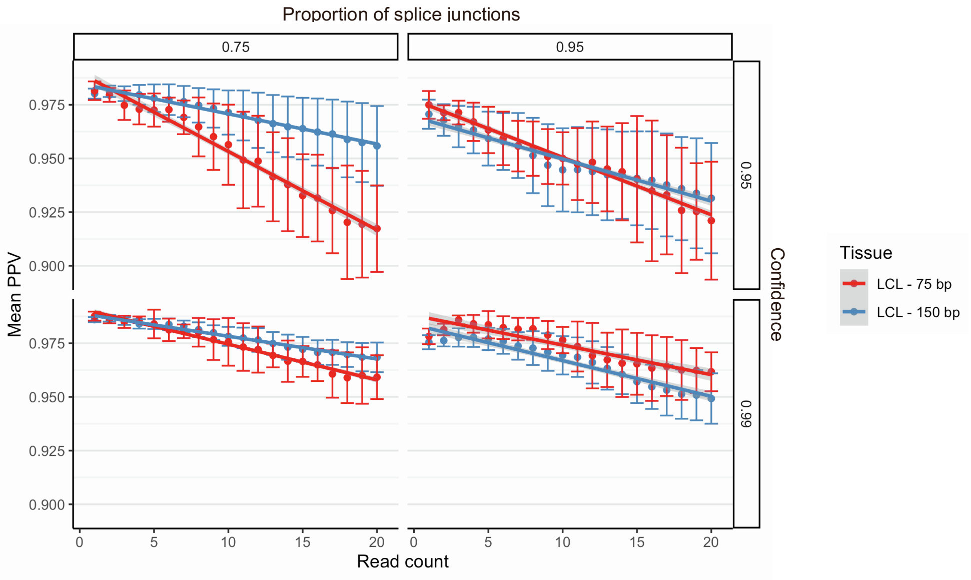

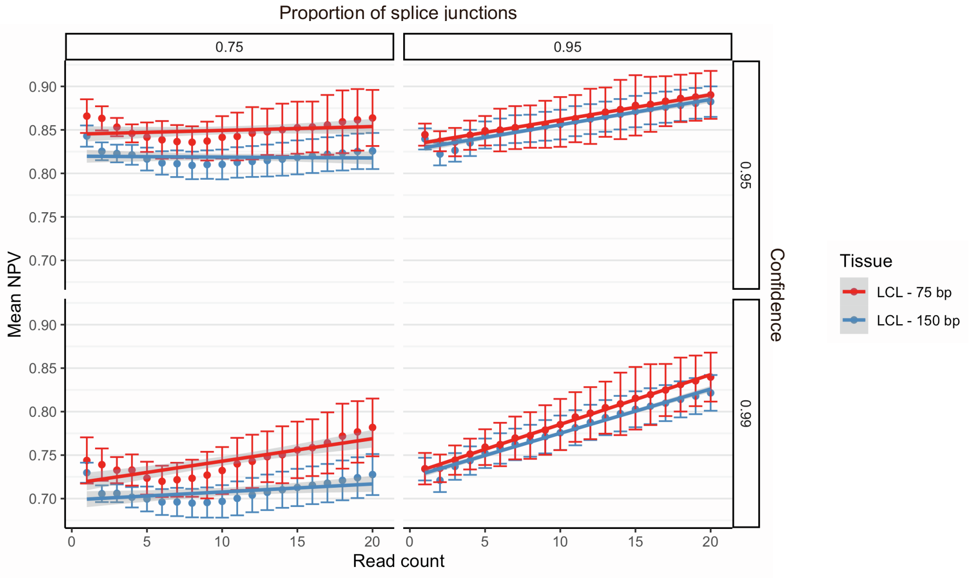

Figure S6. ***Effect of varying sequencing read length on MRSD model performance.*** Despite being derived from 75 bp paired end RNA-seq data, MRSD scores show similar performance when applied to 75 or 150 bp paired end read-based RNA-seq, both in terms of (top) PPV and (bottom) NPV. When specifying 75% splice junction coverage, MRSD PPV is generally higher when the model is applied to 150 bp read-based data. This likely reflects the fact that junctions predicted to be sufficiently covered by 75 bp reads will be more likely to be sufficiently covered by reads of greater length, and so positive predictions are more likely to hold true when applied to longer-read data. We also observe that NPV for 150 bp read datasets is lower than that for 75 bp across all 4 parameter combinations; conversely to PPV, this is possibly because transcripts not sufficiently covered by 75 bp reads are more likely to be sufficiently covered by 150 bp reads, thus making negative predictions less likely to hold true in longer-read data. In most cases, differences in model performance between 75 and 150 bp is low, suggesting MRSD may, in some cases, provide a suitable approximation of transcript coverage in RNA-seq datasets with read lengths different to those used to construct the model.

Figure S7

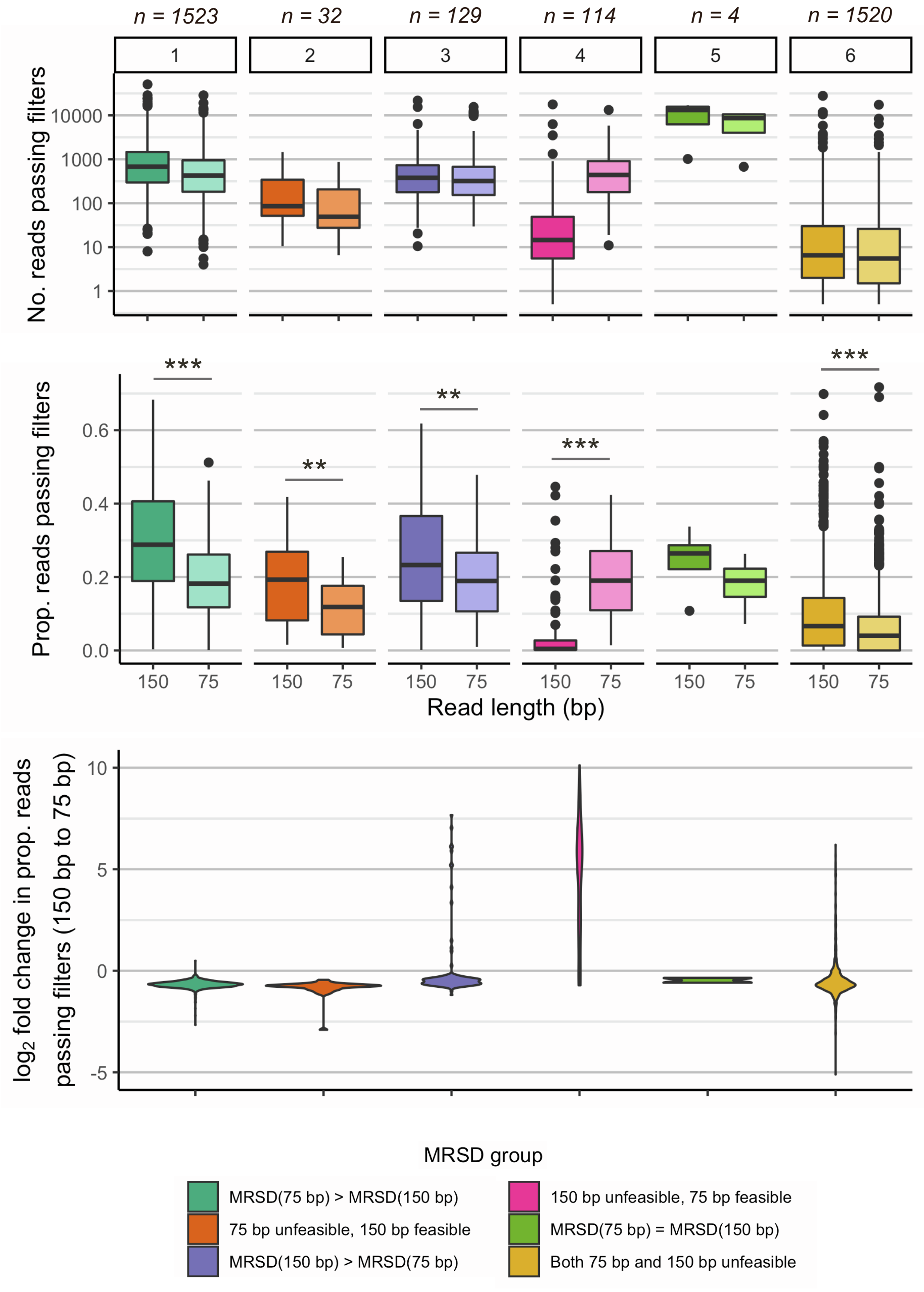
n = 1523
n = 32
n = 129
n = 114
n = 4
n = 1520
1
2
3
4
5
6
No. reads passing filters
10000
1000
100
10
1
Prop. reads passing filters
0.6
0.4
0.2
0.0
***
**
**
***
***
150
75
Read length (bp)
log2 fold change in prop. reads passing filters (150 bp to 75 bp)
10
5
0
-5
MRSD group
MRSD(75 bp) > MRSD(150 bp)
75 bp unfeasible, 150 bp feasible
MRSD(150 bp) > MRSD(75 bp)
150 bp unfeasible, 75 bp feasible
MRSD(75 bp) = MRSD(150 bp)
Both 75 bp and 150 bp unfeasible

Figure S7. *MRSD scores are generally lower when derived from RNA-seq runs of longer read length.* MRSD predictions generated from 20 LCL-based 150 bp RNA sequencing runs were compared against those generated following trimming of the same reads to a maximum of 75 bp. For 45.8% (1520/3322) of disease-associated genes, coverage was too poor to generate an MRSD score regardless of read length (group 6), while MRSDs could be generated but remained the same regardless of read length for just 4/3322 (0.12%) genes (group 5). Intuitively, of the 54.1% (1798/3322) of genes for which at least one dataset allowed MRSD generation, a higher MRSD was observed in the 75 bp dataset for 86.5% (1555/1798, groups 1 and 2). However, for the remaining 13.5% of genes (243/1798, groups 3 and 4), a lower MRSD score was generated using the 75 bp dataset than the 150 bp dataset. For many of these genes, it was determined that a shortening of the reads actually improved their quality to the extent that they were more likely to pass the enforced quality filters – namely, that a mapping event must be the primary alignment, that the read must map successfully (i.e. must have a mapping quality of 60) and that the read must be a split read. We observed that in group 4, comprising genes for which MRSD generation is unfeasible using the 150 bp dataset but feasible using the 75 bp dataset, there was a median 36.8-fold increase in the number of reads passing these read filters following trimming (bottom). Further work is needed to investigate alternative causes of this counter-intuitive pattern, and to determine whether the discarding of the longer reads represents an artefactual drawback to the read filtering process, or an effective way to filter reads for quality that is missed using shorter reads.

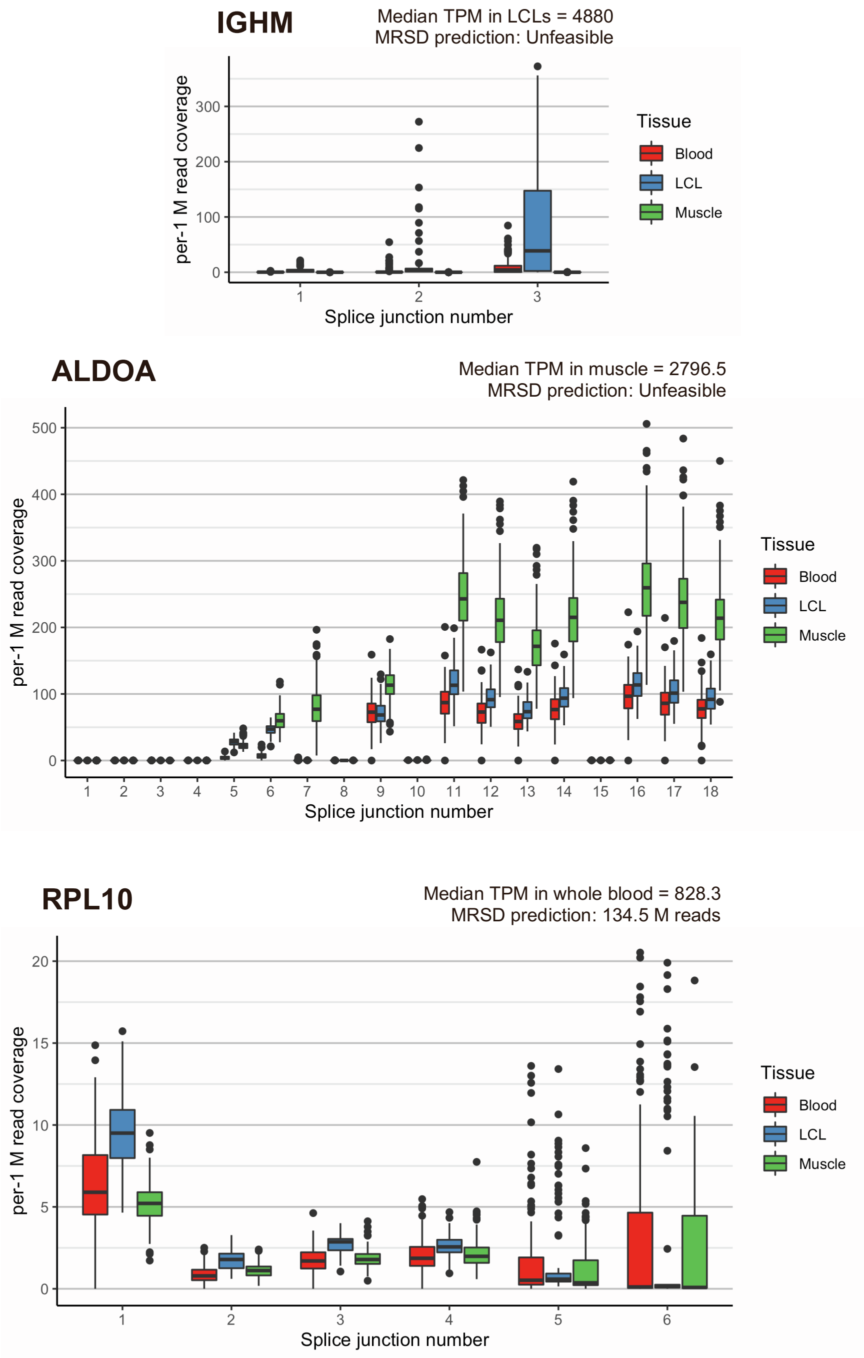

Figure S8. *Evidence for 3' sequence bias confounding the use of TPM as a guiding RNA-seq metric.* Analyzing the number of reads (per 1 M uniquely mapping input reads) mapping to individual splice junctions within three genes with substantial TPM-MRSD discrepancy demonstrates that highly expressed genes may exhibit biased coverage of splice junctions. For IGHM (top) and ALDOA (middle) in LCLs and muscle, respectively, a sufficient proportion of junctions towards the 3' end of the transcript have no read support in a sufficient number of patients, resulting in an MRSD prediction of "unfeasible", despite high coverage of other junctions within the same transcript. Coverage of the final two splice junctions in RPL10 (bottom) in LCL-based RNA-seq data is low but not non-zero in many patients, giving a feasible but high MRSD prediction. In some cases, this bias may result from artefacts of library preparation, or may possible reflect genuine isoform shifts in the given tissue. Higher splice junction numbers represent junctions closer to the 3' end of transcripts.

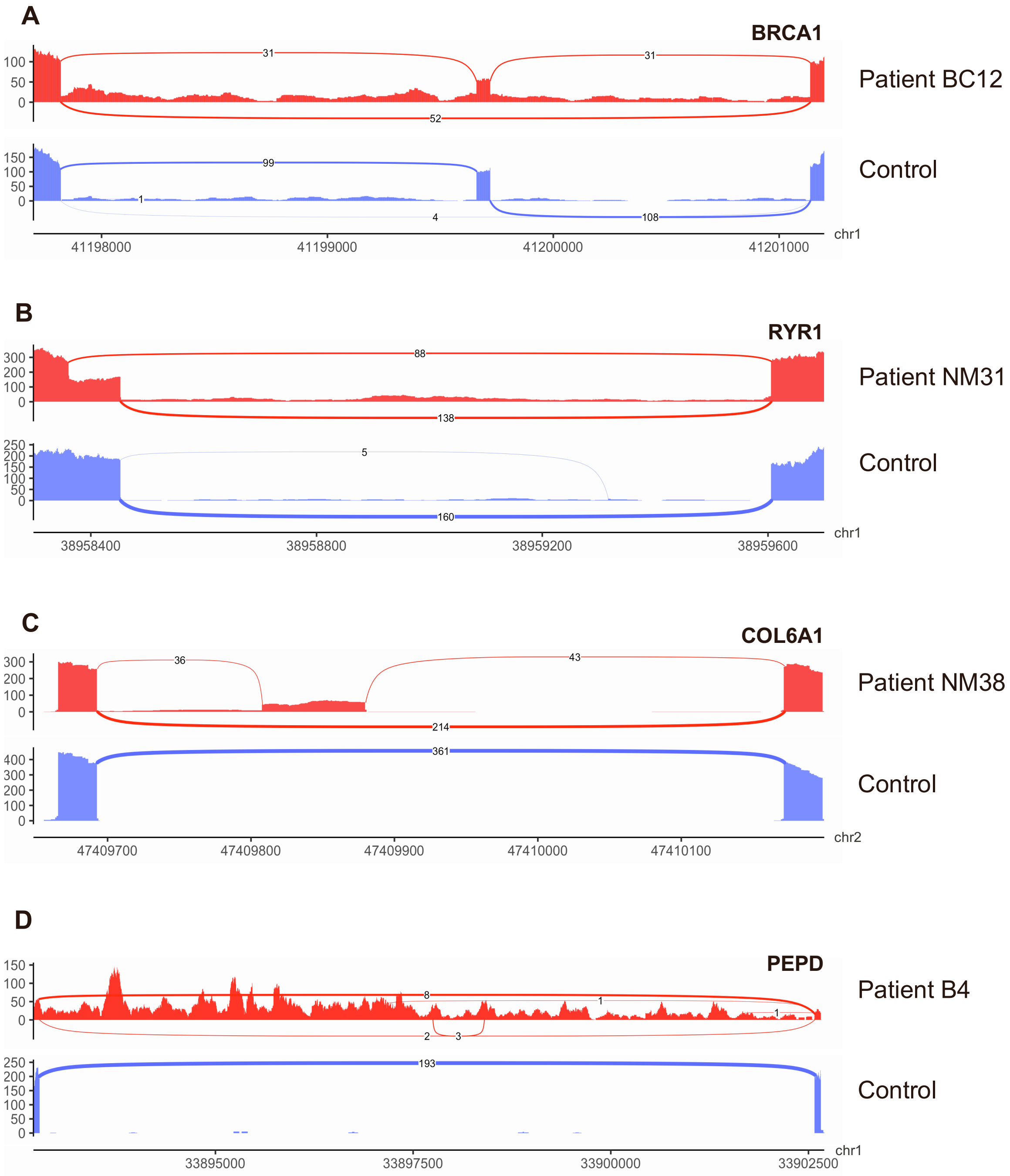

Figure S9. *Exemplar events identified during pathogenic splice event analysis.* Selected Sashimi plots for (**A**) exon skipping, (**B**) exonic splice gain, (**C**) pseudoexonization and (**D**) intron retention events identified as the cause of disease in our patient datasets. The presence of aberrant splice junctions with outlying event metrics allowed flagging of these as potentially pathogenic. For (**D**), the intron retention event was identified from the 2 reads supporting usage of an extremely weak alternative splice acceptor four bases downstream of the abrogated canonical acceptor; however, in the absence of any aberrant splicing events, intron retention events are more difficult to identify from RNA-seq data using current bioinformatics pipelines.

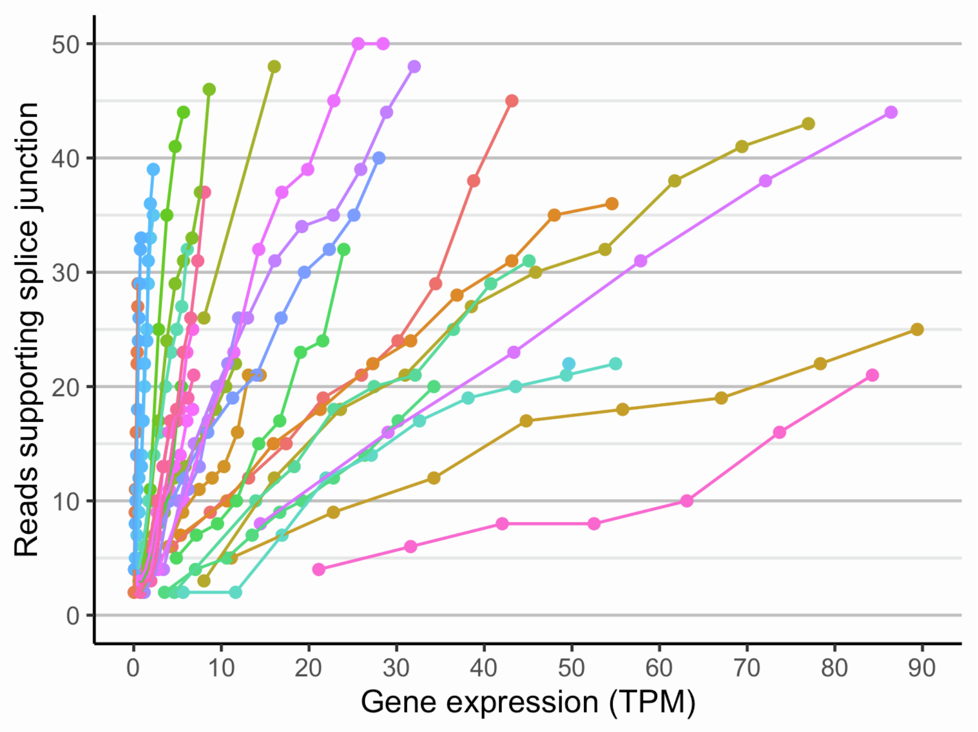

Figure S10. *Relative gene expression level does not reflect the raw read coverage of transcript splice junctions.* When simulating decreased gene expression by downsampling reads in genes containing novel splicing events identified in upstream analysis, it emerged that expression of a gene (in transcripts per million, TPM) does not directly correlate with the number of reads supporting splice junctions in that gene. Among the events supported by 8 reads, for example, gene expression ranged from 0.17-52 TPM. This may be accounted for by variation in the proportion of transcripts containing the event, variation in the coverage across the length of a transcript (as shown in Figure S4), or variation in the depth to which a sample has been sequenced. Thus, when specifying a metric threshold above which we expect splice aberration to be observable, relative expression level may not appropriately represent expected read support. Axes are limited for ease of visualization.

Figure S11. *Pairwise comparisons, by tissue, of predicted MRSD scores for PanelApp disease genes.*

A) MRSD predictions in muscle vs. blood

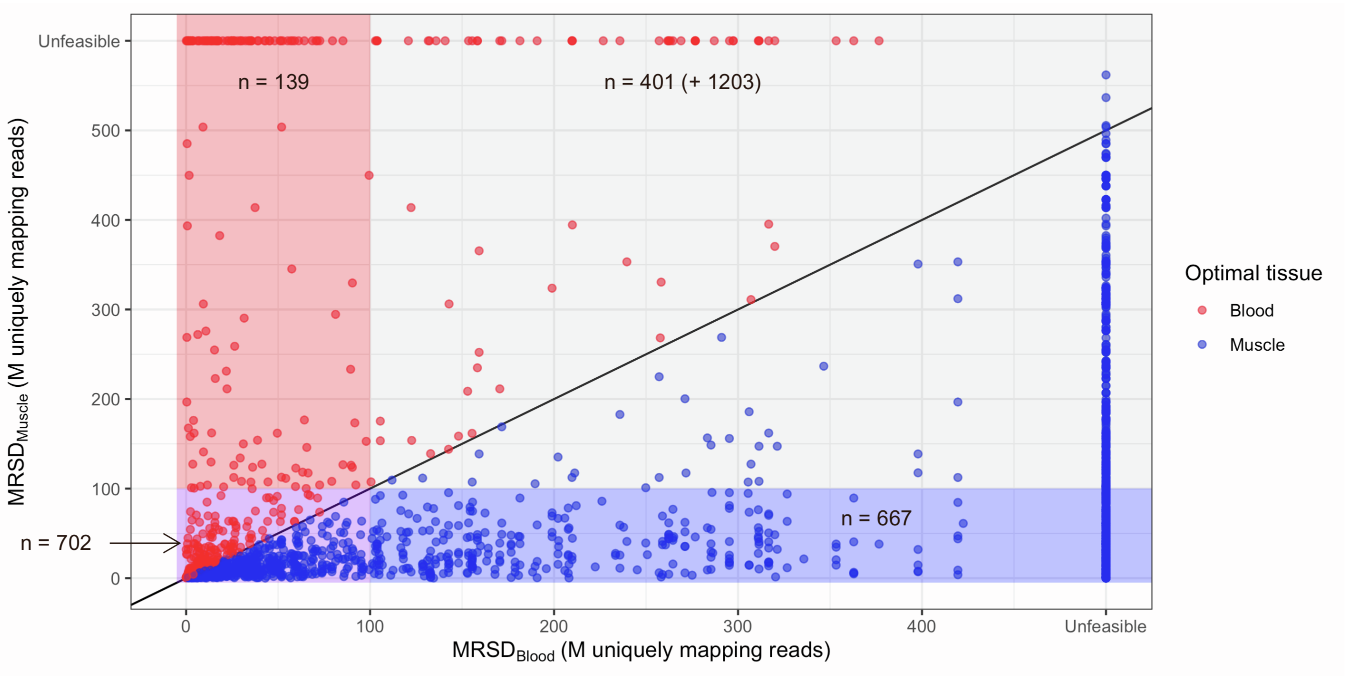

B) MRSD predictions in LCLs vs. muscle

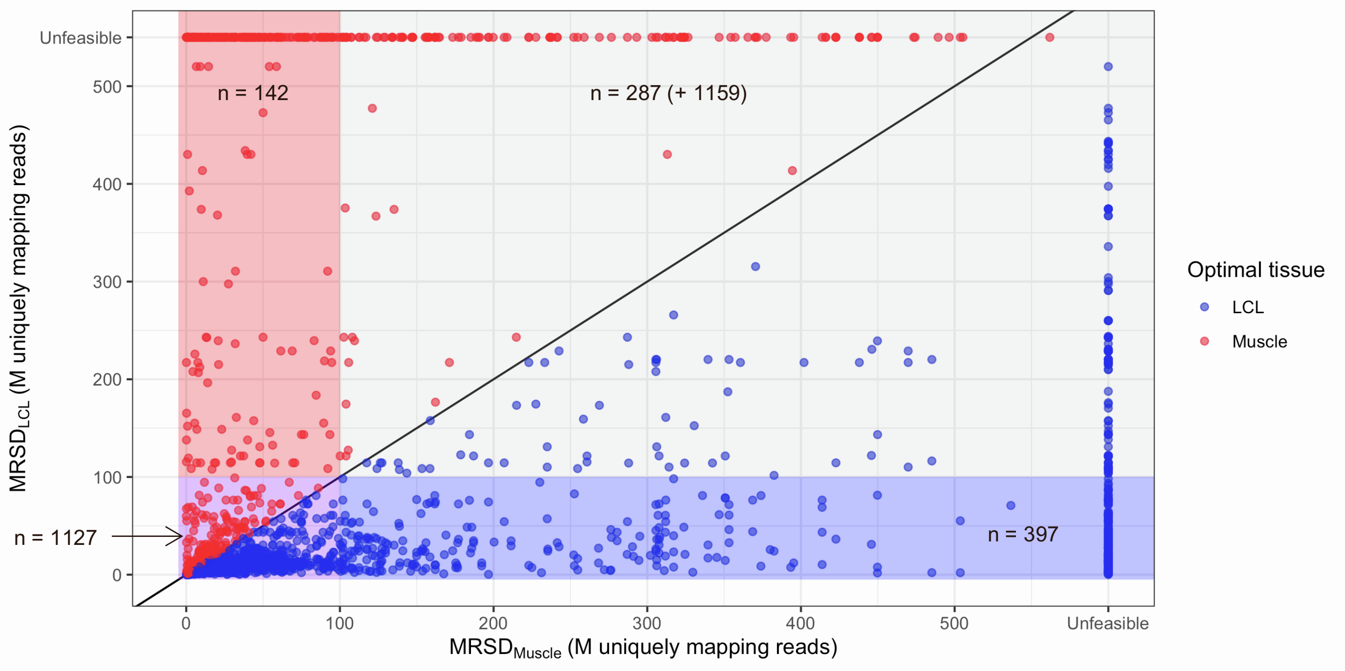

Figure S12. *Proportion of low-MRSD genes per tissue for all PanelApp panels, ordered by panel size.*

A) Low-MRSD gene proportions for large panels (> 50 genes)

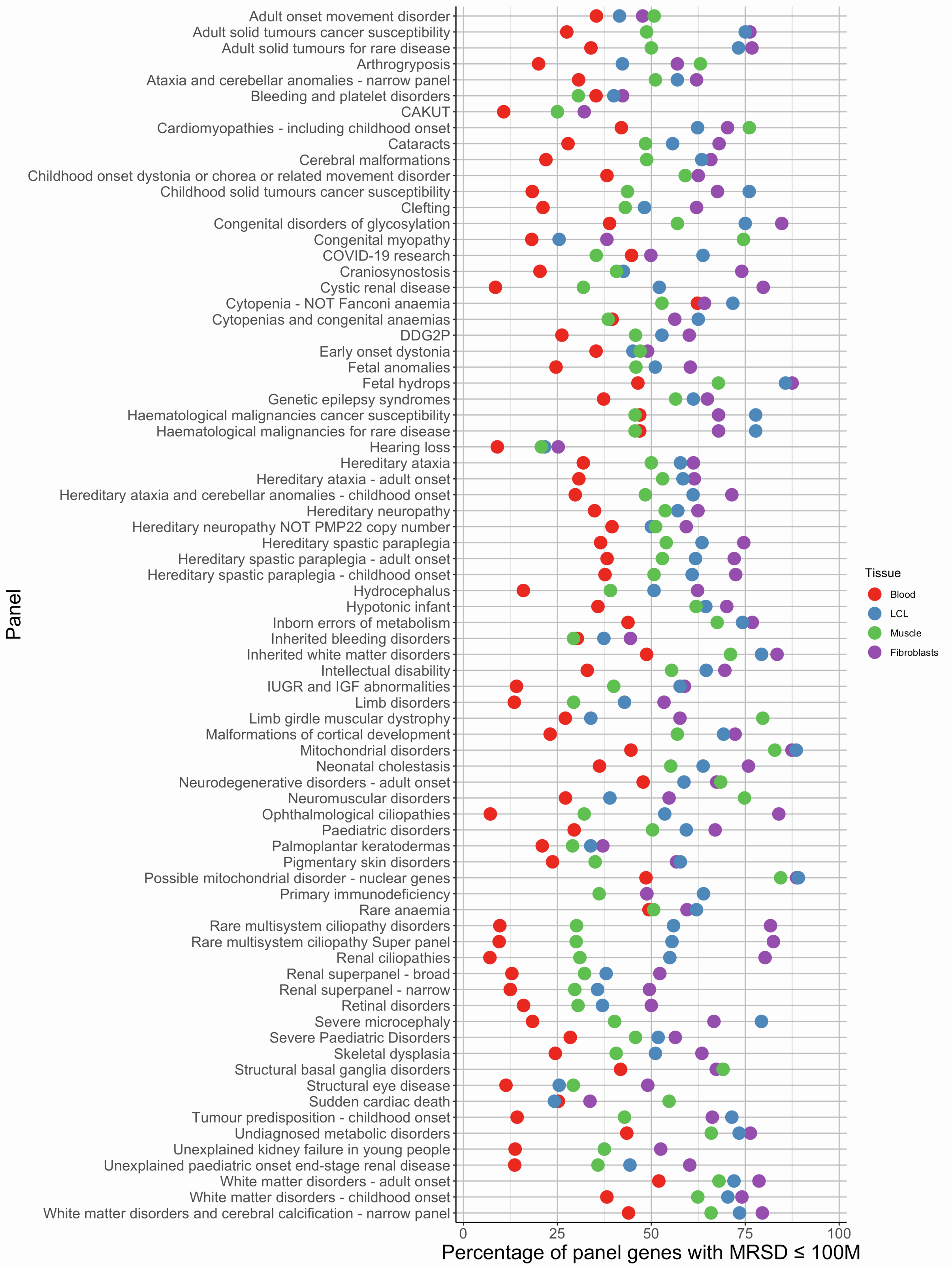

B) Low-MRSD gene proportions for medium panels (21-50 genes)

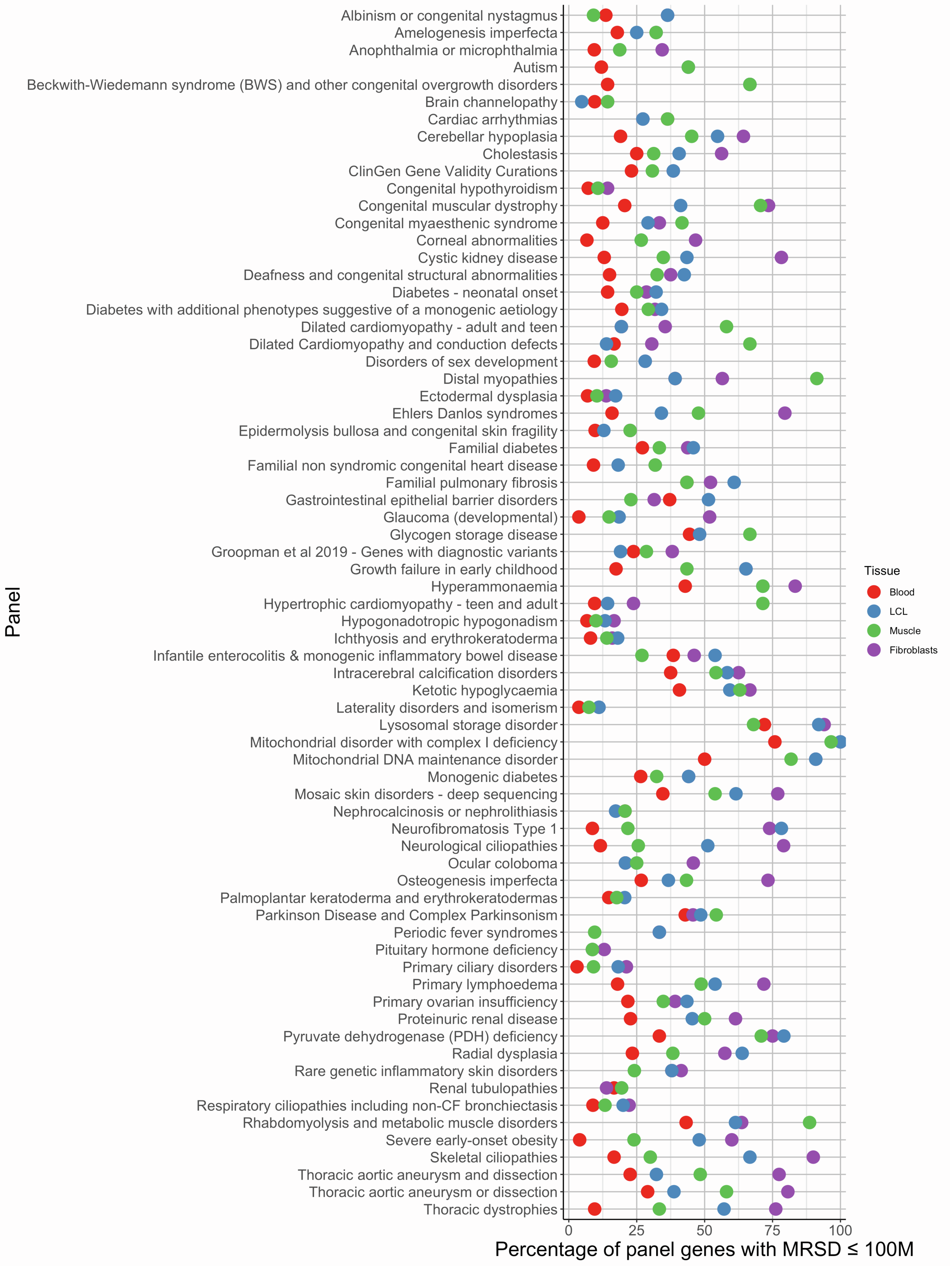

C) Low-MRSD proportions for small panels (11-20 genes)

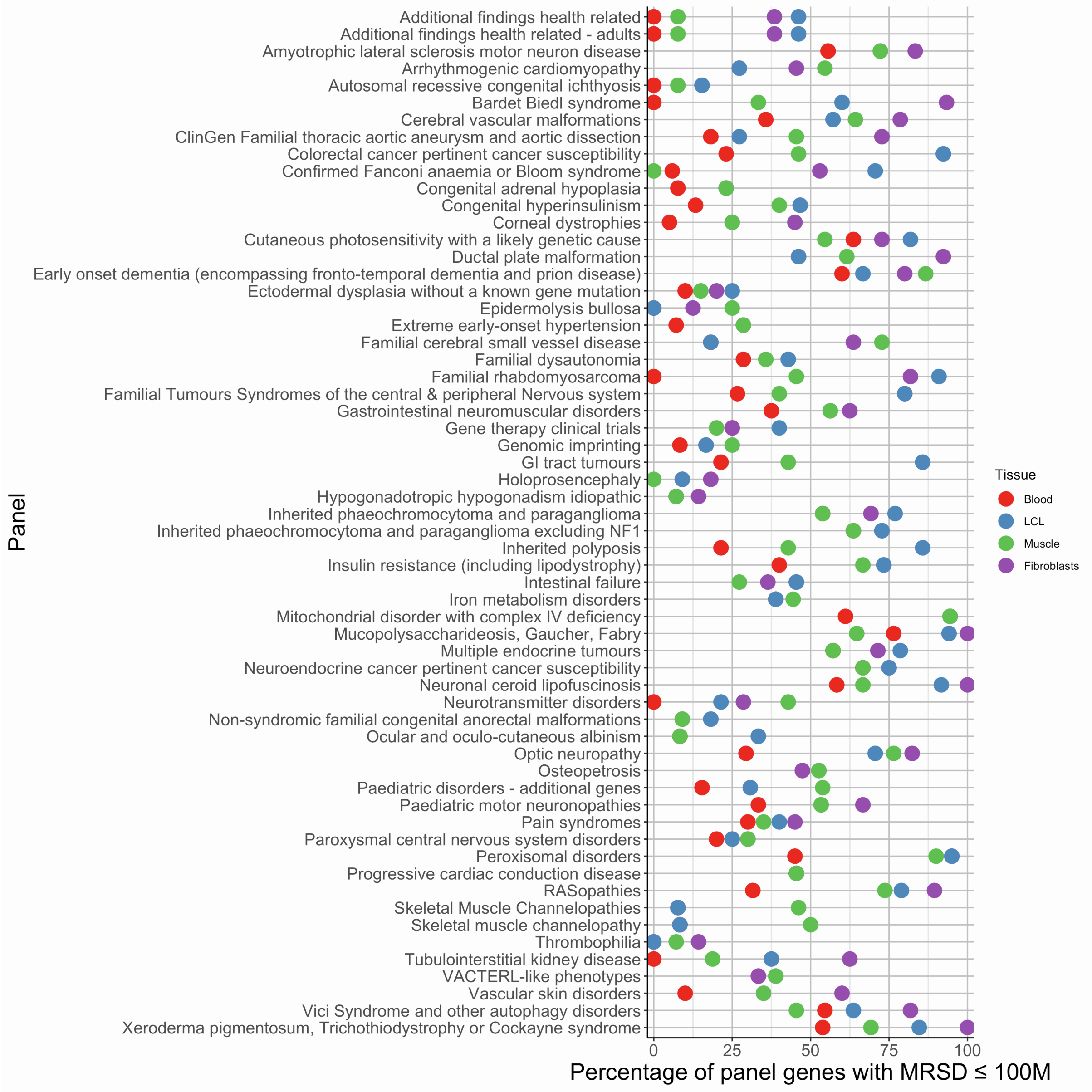

D) Low-MRSD gene proportions for very small panels (≤ 10 genes)

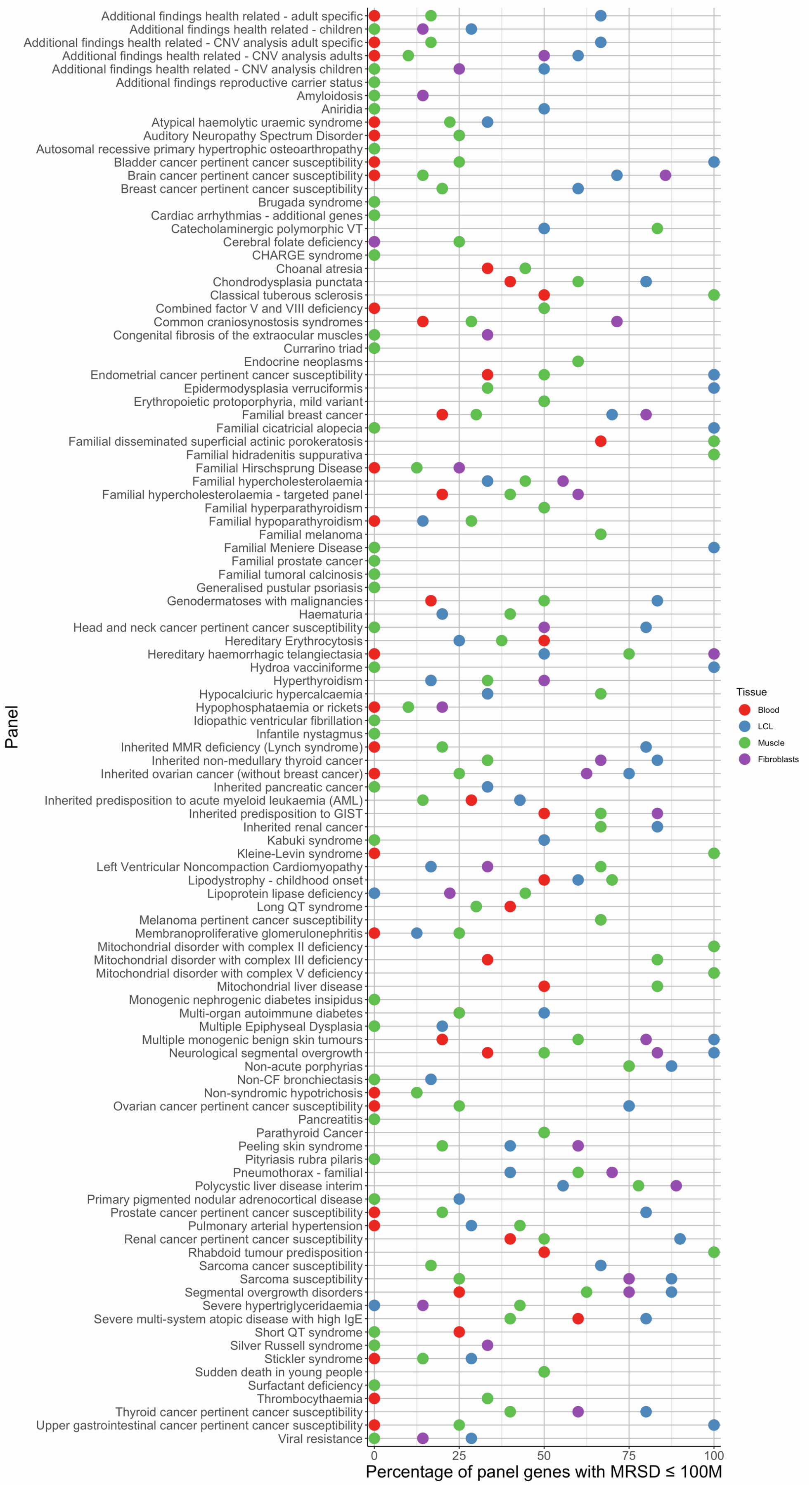

Figure S13. *Proportion of low-MRSD genes per tissue for all PanelApp panels, ordered alphabetically by panel name.*

A) Low-MRSD gene proportions for panels named A-E

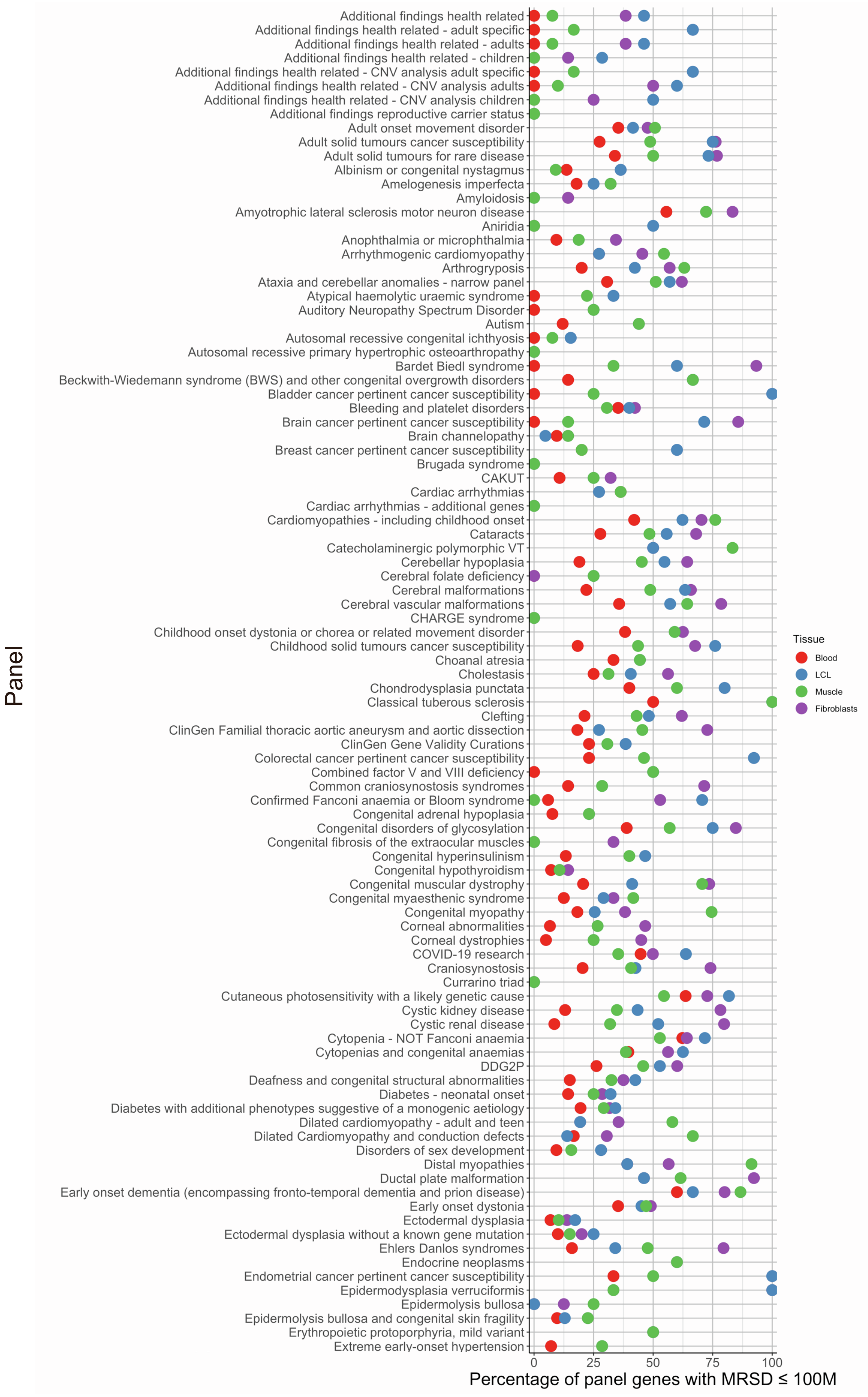

B) Low-MRSD proportions for panels named F-L

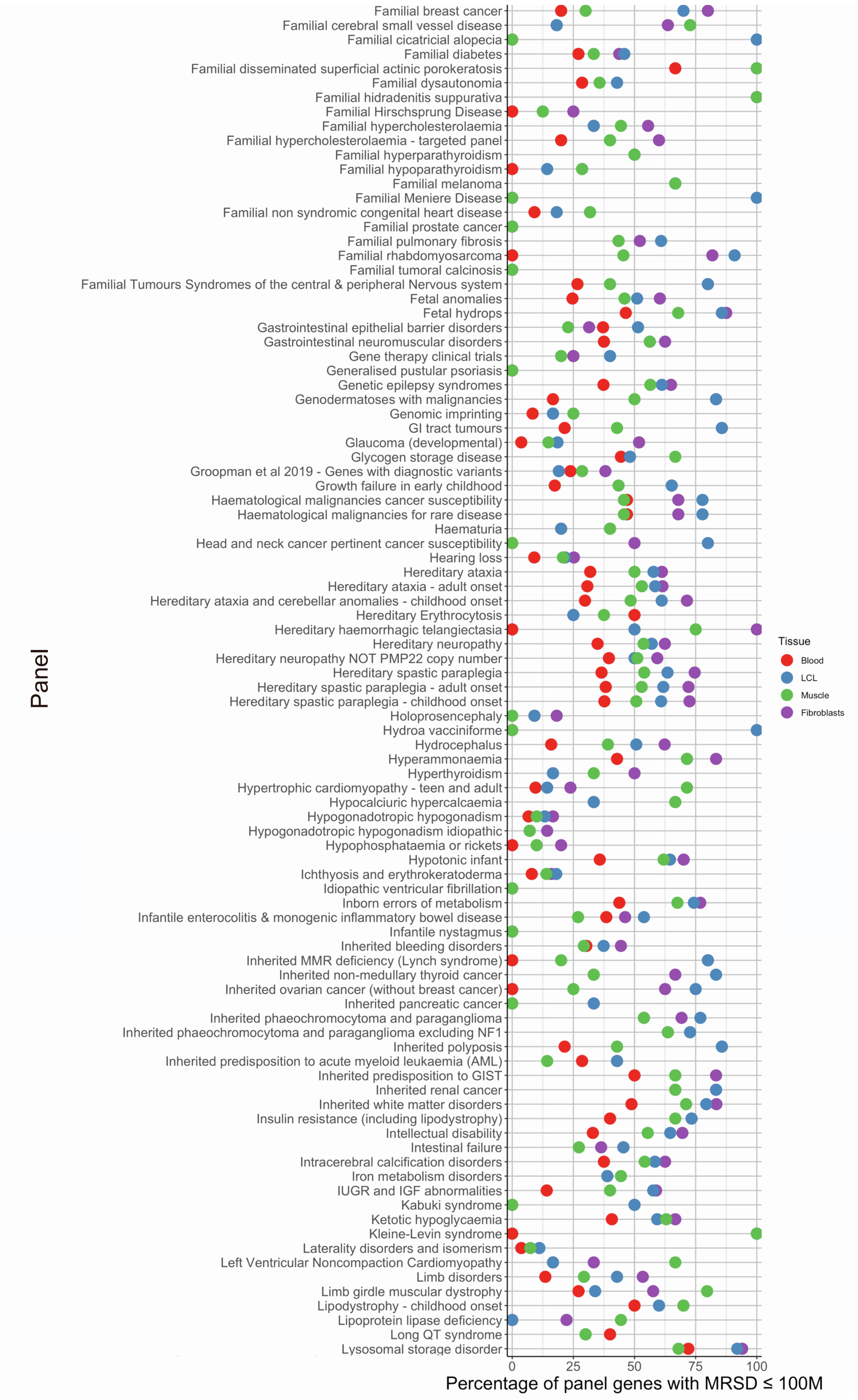

C) Low-MRSD gene proportions for panels named M-R

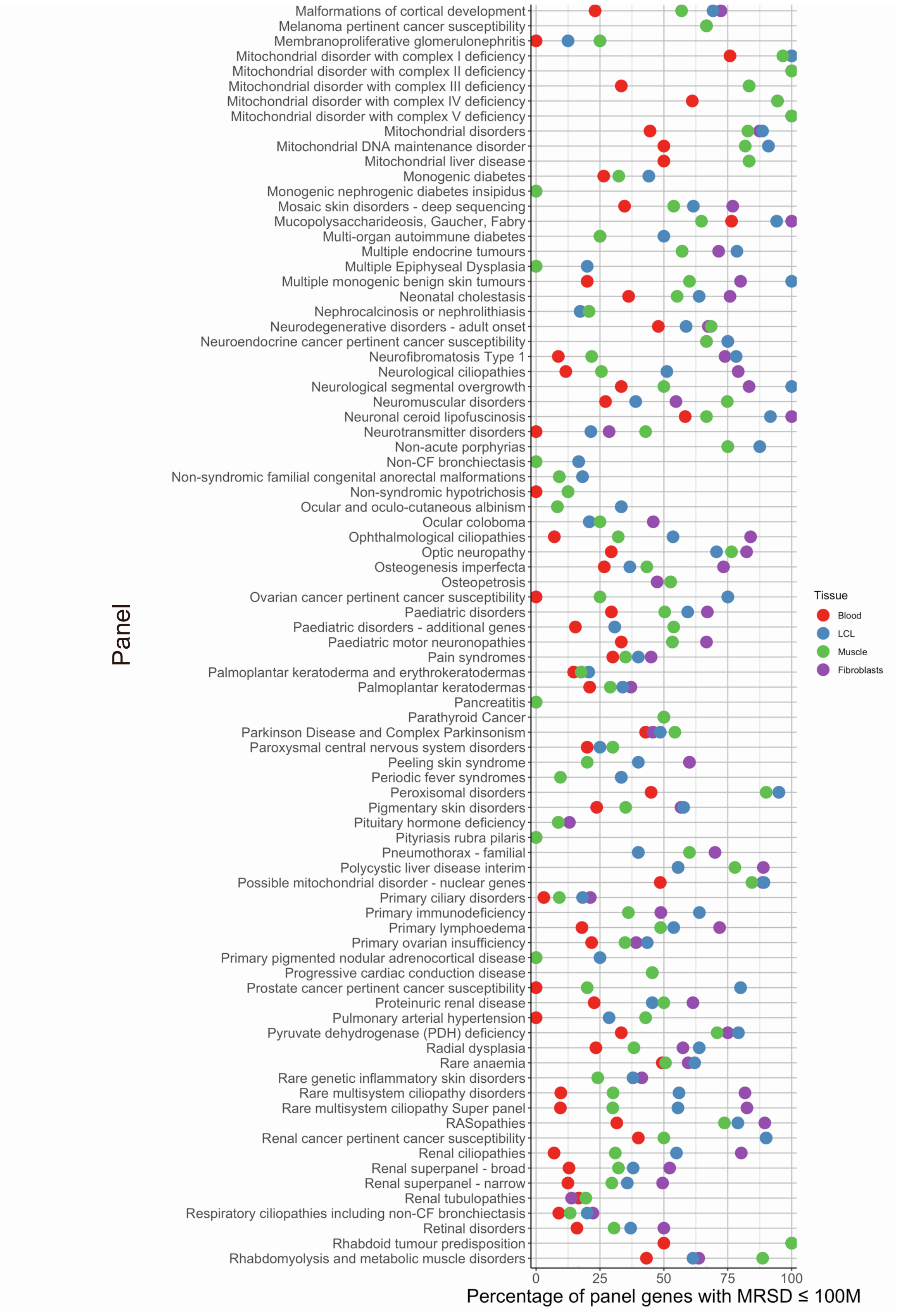

D) Low-MRSD gene proportions for panels named S-X

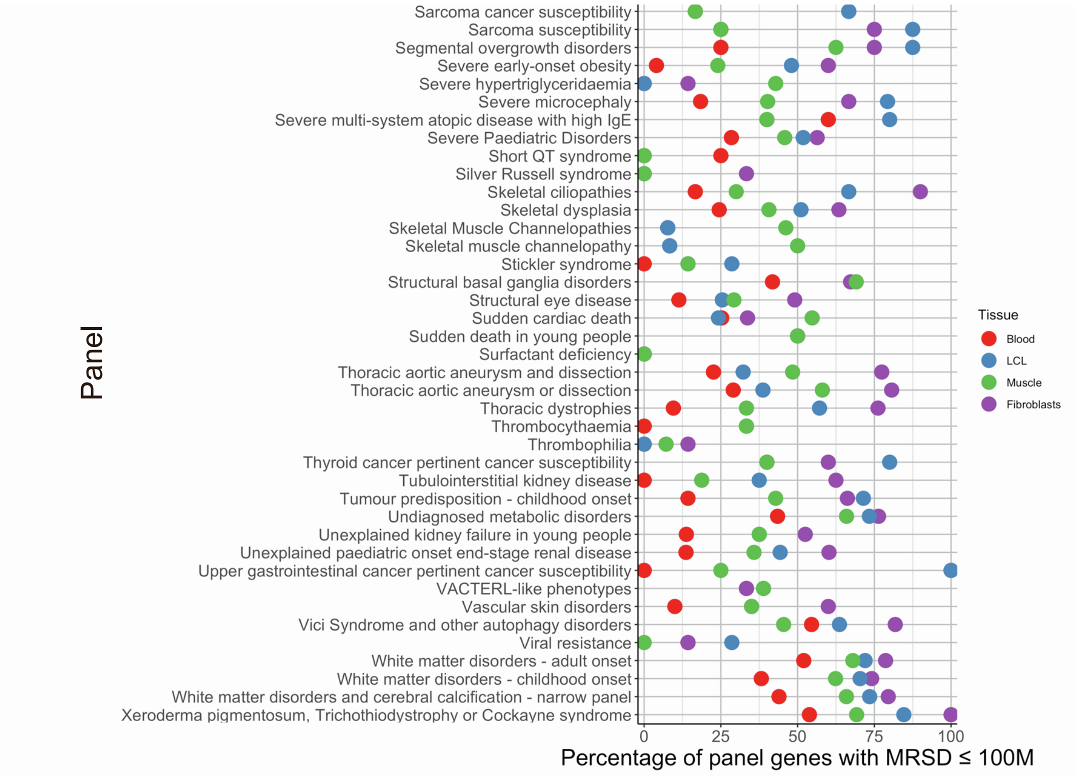

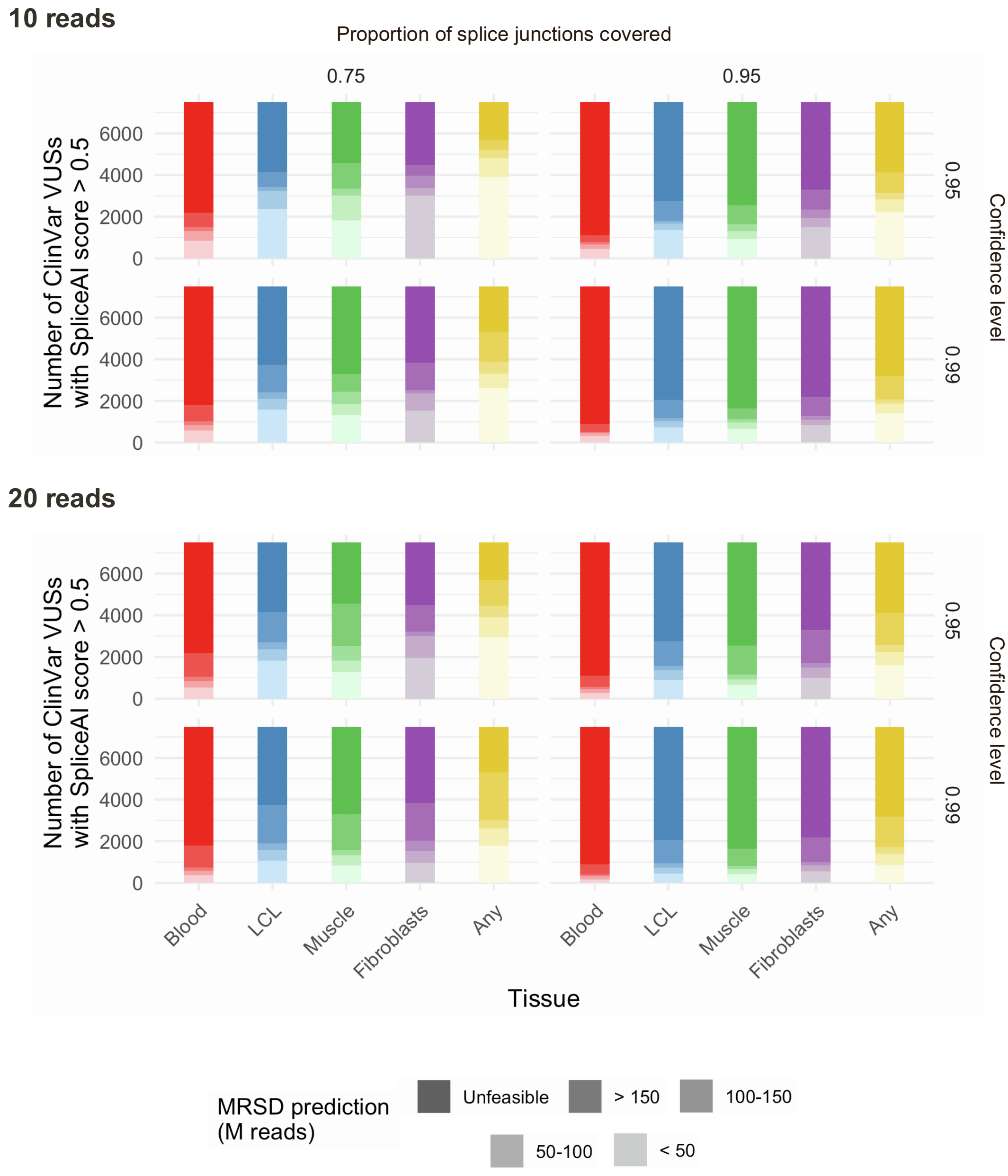

Figure S14. *Increasing specified read coverage reduces the number of ClinVar variants that can be analyzed.* Similarly to Figure 7a (main text), we generated MRSD scores for genes harboring predicted splice-impacting ClinVar variants (SpliceAI score ≥ 0.5 (4)) using more stringent read coverage parameters (10 and 20 reads). We observed only a small reduction in the number of ClinVar variants in low-MRSD genes when specifying 10 reads (24.9-64.0% dependent on parameters). Specifying 20 read coverage, however, dramatically reduces the percentage of ClinVar variants in low-MRSD genes to 18.7-52.0%.

10 reads

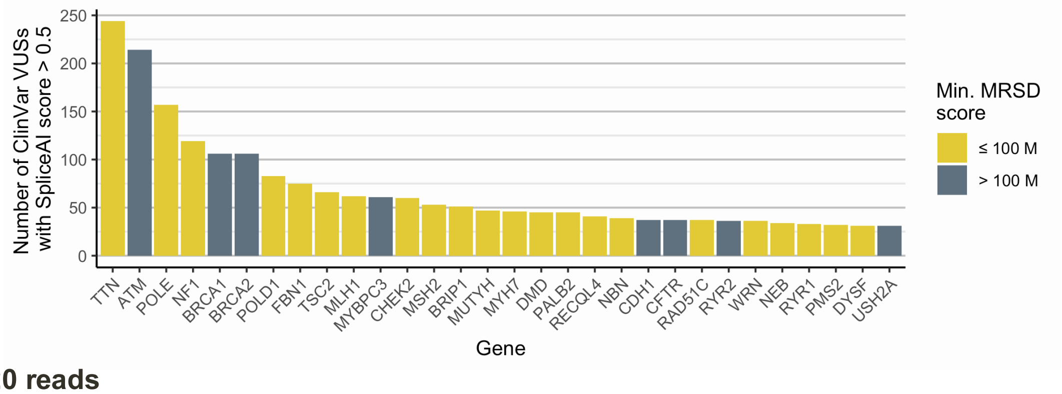

20 reads

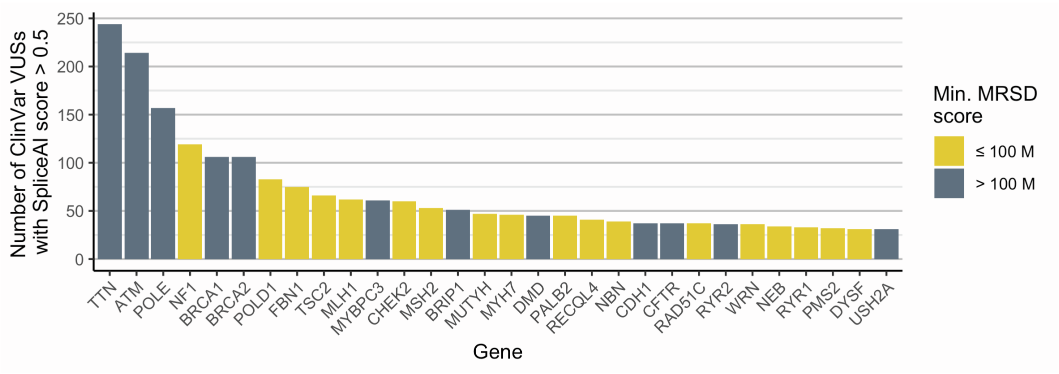

Figure S15. *Increasing specified read count removes highly VUS-prone genes from the scope of analysis.* Similarly to Figure 7b, we looked among the 30 genes harboring the most predicted splice-impacting ClinVar variants and considered how many were low-MRSD in at least one of the four investigated tissues when specifying increasing levels of read coverage. Only one extra gene, ATM, becomes ostensibly high-MRSD when specifying a 10-read coverage parameter when compared with the 8-read coverage data (Figure 7b). However, by specifying a 20-read level of coverage, a further four genes are removed from the scope of analysis, leaving 18/30 (60%) still considered low-MRSD.

Variant (HGVSg)	Gene	Source of RNA	Phenotype	TPM	MRSD (M reads)
chr2:152,355,017G>T	NEB	Skeletal muscle	Nemaline myopathy	857.9	9.83
chr2:152,389,953A>C					
chr2:152,544,805C>T					
chrX:31,790,694-31,798,498invdel	DMD		Duchenne muscular dystrophy	24.84	79.4
chrX:32,274,692G>A			Myalgia, myoglobinuria		
chr2:179,446,219ATACT>A	TTN		Fetal akinesia	349.5	47.63
chr2:179,642,185G>A			Multi/minicore congenital myopathy		
chr21:47,409,881C>T	COL6A1		Collagen VI-related dystrophy	56.02	16.25
chr21:47,409,881C>T					
chr19:38,958,362C>T	RYR1		Congenital fiber-type disproportion	425.5	3.45
chr1:46,655,129C>A	POMGNT1		α-Dystroglycanopathy	29.26	6.01
chr17:41,199,655C>G	BRCA1	LCL	Inherited breast cancer susceptibility	19.985	217.19
chr17:41,246,879T>C					
chr17:41,246,879T>C					
chr17:41,246,879T>C					
chr17:41,258,551C>A					
chr13:32,945,238G>A	BRCA2			10.16	Unfeasible
chr13:32,969,074A>T					
chr19:33,892,776C>T	PEPD	Whole blood	Prolidase deficiency	18.89	28.31
chr20:35,526,363C>G	SAMHD1		Aicardi-Goutières syndrome	48.53	24.68
chr23:153,997,595G>A	MED13L		MRFACD	5.89	262.34

Table S1. *Summary of pathogenic splicing events analyzed in this study.* All co-ordinates are given in relation to the GRCh37 genome build. TPM, transcripts per million; MRSD, minimum required sequencing depth.

Tissue	No. samples	Source	Sequencing type	Usage
Blood	151	GTEx	75-bp paired end poly-A enrichment, Illumina	Generation of MRSD model, bootstrapping analysis of event counts
LCL	91	GTEx	75-bp paired end poly-A enrichment, Illumina	Generation of MRSD model, bootstrapping analysis of event counts
Muscle	184	GTEx	75-bp paired end poly-A enrichment, Illumina	Generation of MRSD model, bootstrapping analysis of event counts
Blood	1	Inhouse	150-bp paired end globin depletion, Illumina	Collation of known pathogenic mis-splicing events
Blood	12	Inhouse	75-bp paired end poly-A enrichment, Illumina	Collation of known pathogenic mis-splicing events & MRSD model validation
LCL	20	Inhouse	150-bp paired end poly-A enrichment, Illumina	Collation of known pathogenic mis-splicing events
LCL	4	Inhouse	75-bp paired end poly-A enrichment, Illumina	MRSD model validation
Muscle	52	Previously published data (3)	75-bp paired end poly-A enrichment, Illumina	Collation of known pathogenic mis-splicing events, downsampling of pathogenic events & MRSD model validation

Table S2. *Summary of RNA-seq datasets utilized in this study*. RNA-seq datasets derived using different methodologies were used for various aspects of this study. All data used to generate the MRSD model was based on data from the GTEx consortium across all three analyzed tissues.

Methods S1.

Minimum required sequencing depth (MRSD) score (further elaboration).

MRSD is defined for an individual transcript in a given sample as:

$$MRSD_m = r/\left(\frac{R_p}{d}\right)$$

Where r is the desired level of read coverage across desired proportion p of splice junctions, R is the set of read counts supporting each of the splice junctions in the transcript of interest, ordered from lowest to highest, and R_p is the read count at the position in R at which proportion p of read counts values in R are greater than or equal to it. d represents the total number of sequencing reads, in millions of reads, in the RNA-seq sample (by default, the number of uniquely mapping sequencing reads), and (m) represents the MRSD parameter.

For instance, suppose a sample sequenced to a depth (d) of 40 M uniquely mapping sequencing reads generates coverage of 14, 16, 6 and 10 reads across the splice junctions of a five-exon transcript. Suppose we wish 75% of splice junctions to be covered by a minimum of 6 reads (i.e. p = 0.75 and r = 6). Here, R = {6, 10, 14, 16} and R_p = 10, as 3/4 (75%, i.e. p) of all values in R are greater than or equal to 10. Inserting these values into the formula shows that this transcript has an MRSD of $\frac{6}{10/40} = 24\,M$ uniquely mapping sequencing reads in this sample.

The set of MRSD scores for the given transcript are then collated across all control samples and ordered from lowest to highest. The score at the m-th percentile position in the collated list of sample-specific MRSDs is returned as the overall MRSD for that transcript, where m is termed the “MRSD parameter” and is customizable by the user (default = 0.95). The $MRSD_{0.99}$ of a transcript represents the sequencing depth that would be required for 99% of control samples to achieve the specified coverage for that transcript. The MRSD parameter therefore approximately represents the likelihood that a sequencing run at the returned depth will yield the desired coverage level.

Illustration of MRSD calculation methodology. MRSD scores utilize the level of read coverage supporting the existence of splice junctions in control RNA-seq datasets to predict the depth of sequencing required to achieve a specified level of splice junction coverage in a transcript of interest. For a given transcript in a given individual:

1. Read coverage values are collated across all splice junctions in the transcript model (with a single transcript assigned to each gene if investigating at the gene level, see Methods S2, below)
2. Each of these values is divided by the sequencing depth – by default defined as the number of uniquely mapping sequencing reads (in millions of reads) to produce a per-1 M read coverage value for each junction
3. The desired level of read coverage is divided by the per-1 M read coverage value of the splice junction with the *X*'th percentile lowest read coverage, which gives the depth of sequencing that would be required for X% of junctions to be covered with the desired number of reads or higher. This figure is the sample-specific MRSD.

The sample-specific MRSDs are collated across all control RNA-seq samples, and a global MRSD is then derived by taking the m-th percentile highest prediction from among these; m is termed the MRSD parameter, and represents the proportion of control RNA-seq samples for which sequencing at the returned MRSD would have sufficiently covered that gene. By

extension, it is also an approximate measure of the likelihood that a subsequent RNA-seq run at the returned depth will yield the specified coverage.

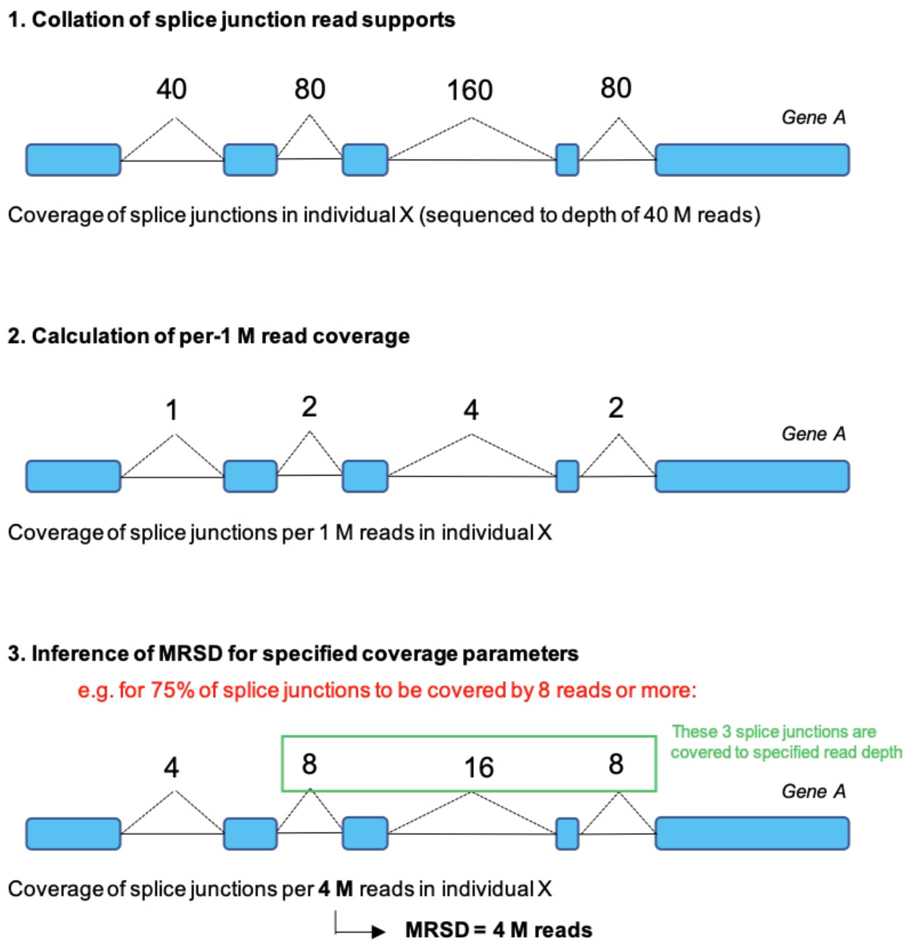

Methods S2. *Tiering methodology for selection of transcripts for MRSD generation.* To calculate MRSD values for all protein-coding genes, a single transcript model was established for each gene. Firstly, transcripts present in the MANE v0.7 curated transcript set were selected for genes where these existed, provided the co-ordinates of all splice junctions in that transcript (given in relation to the GRCh38 reference genome) mapped back to known junctions in build GRCh37. For genes where these conditions were not met, transcript models were formed from the union of all junctions present in all RefSeq transcripts listed for that gene on Ensembl BioMart. Finally, for any genes lacking a corresponding RefSeq transcript(s), a transcript model was derived consisting of the union of all junctions present in all transcripts assigned to that gene in the GENCODE v19 annotation.

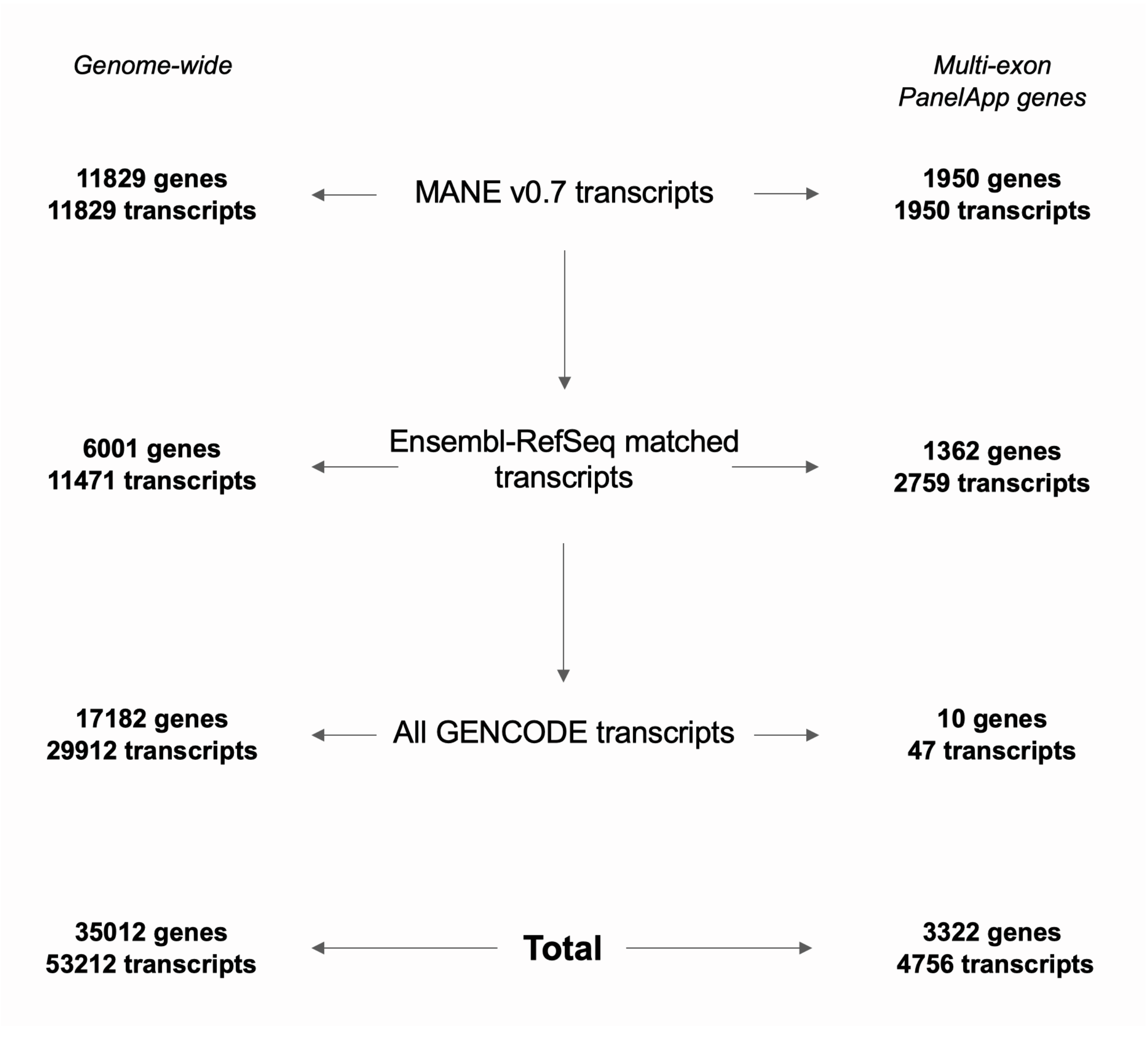

Methods S3. *Tissue-specific criteria for filtering of high-quality GTEx control RNA-seq datasets.* Filtering of GTEx controls was conducted to select the highest quality samples based on the below tissue-specific parameters. Parameters were selected and adjusted on a tissue-by-tissue basis to exclude metric outliers and samples that may confound analysis of pathogenic splicing events (e.g. excluding cancer patients from LCL control cohorts, in which inherited breast cancer was studied). The corresponding column names in the GTEx v8 sample attribute (pht002743.v8) and subject phenotype (pht002742.v8) files are italicized.

Skeletal muscle (as listed in [1])

- RNA integrity number/RIN (*SMRIN*): between 6-9
- Sample ischemic time (*SMTSISCH*): <720 (i.e. <12 hours)
- Hardy scale (*DTHHRDY*): 0, 1 or 2, corresponding to sudden deaths
- Age (*AGE*): <50
    - Unless BMI <30

Whole blood

- Samples included in GTEx analysis freeze, corresponding to higher quality samples (*SMAFRZE*): not flagged EXCLUDE due to technical issues
- RIN (*SMRIN*): between 6-9
- Sample ischemic time (*SMTSISCH*): <0
- Hardy scale (*DTHHRDY*): 0, 1 or 2

EBV-transformed lymphocytes (LCLs)

- *SMAFRZE*: not flagged EXCLUDE due to technical issues
- RIN (*SMRIN*): > 9
- *MHCANCER5*, *MHCANCERC* and *MHCANCERNM* all 0 to eliminate all non-metastatic cancers and all cancers in the past 5 years or current
- *DTHHRDY*: 0, 1 or 2
- No reported history (*MHGENCMT*) of:
    - Breast cancer
    - Ovarian cancer
    - Pancreatic cancer
    - Prostate cancer
    - Colorectal cancer
    - No patients filtered out through this criterion

Cultured fibroblasts

- As for EBV-transformed lymphocytes, except with the addition of the following:
    - RIN (*SMRIN*) > 9.7
    - Uniquely mapping reads (*MPPDUN*): > 60 M

Methods S4. *Sample IDs of GTEx samples used to generate control datasets.*

Skeletal muscle *(as listed in [1])*

GTEX-111CU-2026
GTEX-111YS-2326
GTEX-1122O-2426
GTEX-113JC-2726
GTEX-117YX-2526
GTEX-11DXX-2726
GTEX-11DXZ-2426
GTEX-11EM3-2126
GTEX-11EMC-2626
GTEX-11EQ9-2126
GTEX-11I78-2426
GTEX-11LCK-1226
GTEX-11NSD-2026
GTEX-11P81-2526
GTEX-11P82-1826
GTEX-11VI4-1926
GTEX-11WQC-2626
GTEX-11WQK-0726
GTEX-11XUK-2226
GTEX-11ZTT-2626
GTEX-11ZVC-2726
GTEX-1211K-2126
GTEX-12BJ1-2526
GTEX-12C56-1926
GTEX-12WSJ-1726
GTEX-12WSN-2526
GTEX-12ZZX-0326
GTEX-12ZZY-0626
GTEX-13111-2226
GTEX-1314G-1726
GTEX-131XF-2326
GTEX-131XG-2326
GTEX-132AR-1026
GTEX-132NY-0726
GTEX-1339X-2426
GTEX-133LE-2026
GTEX-1399Q-2426
GTEX-1399R-2526
GTEX-1399S-2726
GTEX-1399U-2526
GTEX-139D8-0726
GTEX-139UW-2626
GTEX-139YR-2526
GTEX-13CF3-1826
GTEX-13D11-2526
GTEX-13FH7-2126
GTEX-13FHO-0726
GTEX-13FTW-2326
GTEX-13FTY-0226
GTEX-13FXS-0326
GTEX-OIZH-1626
GTEX-OOBJ-1626
GTEX-P4PP-1626
GTEX-P4PQ-1626
GTEX-P78B-1626
GTEX-POMQ-1926-SM-3NB1Y
GTEX-POYW-0526-SM-2XCEY
GTEX-PSDG-0426
GTEX-PWCY-2026
GTEX-Q2AH-1826-SM-2S1Q2
GTEX-Q734-2026-SM-3GADA
GTEX-QCQG-2126-SM-2S1P8
GTEX-QDVN-2426-SM-2S1Q4
GTEX-QV44-2026-SM-2S1RD
GTEX-R53T-1826-SM-3GIJX
GTEX-R55D-0626-SM-3GAD5
GTEX-S32W-2326-SM-2XCAW
GTEX-S33H-2226
GTEX-S7SF-2026-SM-3K2AS
GTEX-SNMC-1426-SM-2XCFM
GTEX-SUCS-1626-SM-32PLS
GTEX-T5JC-0626-SM-3NMA6
GTEX-T5JW-1826-SM-3GAE1
GTEX-TKQ2-0826-SM-33HB6
GTEX-TML8-1826-SM-32QOR
GTEX-TMMY-0426-SM-33HBB
GTEX-U3ZG-0326-SM-47JXN
GTEX-U3ZH-1926-SM-4DXTR
GTEX-U3ZM-1226-SM-3DB9G
GTEX-U4B1-1626-SM-3DB8N
GTEX-UJHI-1726-SM-3DB9B
GTEX-UJMC-1826-SM-3GADT
GTEX-VUSG-2626-SM-4KKZI
GTEX-WHPG-2226-SM-3NMBO
GTEX-WHSB-1826-SM-3TW8M
GTEX-WOFM-1326-SM-3MJFR
GTEX-WRHK-1626-SM-3MJFH
GTEX-WRHU-0826-SM-3MJFN
GTEX-WXYG-2526-SM-3NB3F
GTEX-WY7C-2526-SM-3NB2N
GTEX-WZTO-0826-SM-3NM8Q
GTEX-X4XY-0626-SM-4E3IN
GTEX-X638-0326-SM-47JY1
GTEX-X88G-0326-SM-47JZ4
GTEX-XBEC-0626
GTEX-XBED-2626-SM-4E3J5
GTEX-XBEW-1026
GTEX-XOTO-0526-SM-4B662
GTEX-XPT6-2026-SM-4B64V
GTEX-XQ8I-0626-SM-4BOPT

GTEX-13JUV-2326
GTEX-13N11-2726
GTEX-13N2G-2326
GTEX-13NZ9-0626
GTEX-13NZB-2626
GTEX-13O61-2326
GTEX-13OVG-2126
GTEX-13OVH-0626
GTEX-13OVI-1726
GTEX-13OW6-0626
GTEX-13PL7-0626
GTEX-13PVR-2526
GTEX-13QBU-2426
GTEX-13QJ3-0726
GTEX-13S7M-0326
GTEX-13S86-2326
GTEX-13U4I-1826
GTEX-13VXT-0326
GTEX-13W3W-2626
GTEX-13W46-0726
GTEX-13YAN-0526
GTEX-144GL-0326
GTEX-144GM-2026
GTEX-144GN-2426
GTEX-145LT-1626
GTEX-145LV-2326
GTEX-145ME-2026
GTEX-145MI-0326
GTEX-145MN-2426
GTEX-146FH-0526
GTEX-146FQ-0326
GTEX-147F3-0226
GTEX-1497J-2626
GTEX-14A6H-2826
GTEX-14AS3-2126
GTEX-14BMV-0326
GTEX-14C39-2426
GTEX-14ICL-1926
GTEX-O5YT-1626-SM-32PK6
GTEX-OHPK-1626-SM-2YUN3
GTEX-OHPL-1626
GTEX-OHPM-1626
GTEX-XUJ4-2626-SM-4BOQ3
GTEX-XUW1-0826-SM-4BOP6
GTEX-XUYS-0326-SM-47JX2
GTEX-XUZC-2126-SM-4BRW8
GTEX-XV7Q-2926-SM-4BRUL
GTEX-XYKS-2426-SM-4AT43
GTEX-Y114-2526
GTEX-Y3IK-2626
GTEX-Y5LM-2126
GTEX-Y5V5-2526
GTEX-Y5V6-2626
GTEX-Y8E4-1026
GTEX-Y8E5-0326
GTEX-Y8LW-2026
GTEX-Y9LG-1926
GTEX-YB5E-2226
GTEX-YB5K-2326
GTEX-YBZK-0326
GTEX-YEC3-2126
GTEX-YEC4-2226
GTEX-YF7O-2526
GTEX-YFC4-1026
GTEX-Z9EW-1726
GTEX-ZA64-2026
GTEX-ZAKK-0326
GTEX-ZC5H-0326
GTEX-ZDYS-1726
GTEX-ZPCL-2026
GTEX-ZPIC-2526
GTEX-ZQG8-1226
GTEX-ZQUD-1726
GTEX-ZT9X-1826
GTEX-ZTPG-0126
GTEX-ZTX8-1626
GTEX-ZV6S-2126
GTEX-ZV7C-2426
GTEX-ZVZO-0326
GTEX-ZVZP-2526
GTEX-ZY6K-2026
GTEX-ZYFG-2426
GTEX-ZYWO-2626
GTEX-ZZ64-1526

Whole blood

GTEX-113JC-0006-SM-5O997
GTEX-1192W-0005-SM-5NQBQ
GTEX-11DXX-0005-SM-5NQ8B
GTEX-11EMC-0006-SM-5O9DN
GTEX-11GSP-0006-SM-5N9EL
GTEX-11I78-0005-SM-5N9GB
GTEX-11LCK-0005-SM-5O98U
GTEX-11OF3-0006-SM-5O9CM
GTEX-11ONC-0005-SM-5O9CY
GTEX-11P7K-0006-SM-5N9FM
GTEX-11P82-0006-SM-5N9FY
GTEX-11TT1-0005-SM-5NQ8Y
GTEX-11VI4-0006-SM-5N9D8
GTEX-11WQK-0005-SM-5O9AV
GTEX-11ZTT-0006-SM-5N9FX
GTEX-1212Z-0006-SM-5NQ8M
GTEX-1269C-0005-SM-5N9CJ
GTEX-12C56-0006-SM-5N9E9
GTEX-12KS4-0005-SM-5SI94
GTEX-12WSI-0005-SM-5O99K
GTEX-12WSK-0006-SM-5NQA1
GTEX-12WSM-0005-SM-5NQB3
GTEX-12WSN-0006-SM-5NQAP
GTEX-12ZZX-0005-SM-5O9A9
GTEX-13113-0006-SM-5NQ7X
GTEX-1314G-0005-SM-5NQ9O
GTEX-131XE-0006-SM-5P9F9
GTEX-131XG-0006-SM-5O9CE
GTEX-132NY-0005-SM-5O9AC
GTEX-1399R-0006-SM-5N9FR
GTEX-139UW-0005-SM-5NQ8U
GTEX-13CF3-0006-SM-5N9ED
GTEX-13FTX-0005-SM-5N9F6
GTEX-13FXS-0006-SM-5O99X
GTEX-13OVG-0005-SM-5P9HA
GTEX-13OVH-0005-SM-5P9HB
GTEX-13OVI-0001-SM-5O9BL
GTEX-13OVK-0006-SM-5O9B7
GTEX-13OVL-0006-SM-5O996
GTEX-13OW6-0005-SM-5NQ9Z
GTEX-13OW8-0005-SM-5NQAC
GTEX-13PL7-0005-SM-5N9ET
GTEX-13S7M-0005-SM-5NQ76
GTEX-13VXT-0005-SM-5N9F3
GTEX-147F3-0005-SM-5N9FI
GTEX-147JS-0006-SM-5NQ7K
GTEX-148VI-0006-SM-5O9A6
GTEX-14A5H-0006-SM-5O9AI
GTEX-14AS3-0006-SM-5NQC2
GTEX-14B4R-0006-SM-5O9A7
GTEX-14BMV-0005-SM-5NQ6Y
GTEX-14C38-0006-SM-5NQBF
GTEX-14C39-0005-SM-5NQBR
GTEX-QEG5-0006-SM-2I5FZ
GTEX-QESD-0006-SM-2I5G6
GTEX-R55C-0005-SM-3GAE9
GTEX-RWS6-0005-SM-2XCAN
GTEX-S341-0006-SM-3NM8D
GTEX-SSA3-0005-SM-32QOT
GTEX-T5JW-0005-SM-3GADE
GTEX-T6MN-0005-SM-32PLJ
GTEX-T6MO-0006-SM-32QOU
GTEX-T8EM-0006-SM-3DB71
GTEX-TKQ1-0006-SM-33HBI
GTEX-TKQ2-0006-SM-33HBH
GTEX-TML8-0005-SM-32QPA
GTEX-TMZS-0006-SM-3DB8G
GTEX-U3ZG-0006-SM-47JWX
GTEX-U3ZH-0005-SM-3DB72
GTEX-U4B1-0006-SM-3DB8E
GTEX-UJMC-0005-SM-3GACU
GTEX-UPJH-0006-SM-3GACW
GTEX-V1D1-0006-SM-3NMCE
GTEX-V955-0005-SM-3P5ZC
GTEX-VJYA-0005-SM-3P5ZD
GTEX-VUSG-0006-SM-3GIK9
GTEX-WCDI-0005-SM-3NB2M
GTEX-WFG7-0005-SM-3GIKM
GTEX-WFON-0005-SM-3NMC9
GTEX-WH7G-0005-SM-3NMBX
GTEX-WHPG-0006-SM-3NMBV
GTEX-WHSB-0005-SM-3LK7C
GTEX-WHWD-0005-SM-3LK7D
GTEX-WOFL-0006-SM-3TW8K
GTEX-WOFM-0005-SM-3MJF3
GTEX-WQUQ-0006-SM-3MJF4
GTEX-WRHK-0005-SM-3MJF5
GTEX-WRHU-0006-SM-3MJF6
GTEX-WVLH-0006-SM-3MJF7
GTEX-WXYG-0005-SM-3NB3M
GTEX-WY7C-0006-SM-3NB3L
GTEX-WYVS-0006-SM-3NMA7
GTEX-WZTO-0006-SM-3NM9T
GTEX-X15G-0005-SM-3NMDA
GTEX-X3Y1-0006-SM-3P5ZG
GTEX-X5EB-0006-SM-46MV5
GTEX-X638-0005-SM-47JX6
GTEX-X88G-0006-SM-47JX5
GTEX-XBED-0006-SM-47JXO
GTEX-XBEW-0006-SM-4AT4E
GTEX-XMK1-0005-SM-4B665
GTEX-XPT6-0006-SM-4B66Q
GTEX-XXEK-0005-SM-4BRWJ
GTEX-XYKS-0005-SM-4BRUD
GTEX-Y114-0006-SM-4TT76
GTEX-Y5LM-0005-SM-4V6EJ

GTEX-14DAR-0006-SM-5N9GC
GTEX-14E1K-0006-SM-5N9DY
GTEX-14H4A-0006-SM-5N9E3
GTEX-14ICK-0006-SM-5NQB5
GTEX-14ICL-0006-SM-5SIAB
GTEX-N7MT-0007-SM-3GACQ
GTEX-O5YT-0007-SM-32PK7
GTEX-O5YW-0006-SM-3LK6E
GTEX-OHPL-0006-SM-3MJHB
GTEX-OIZF-0006-SM-2I5GQ
GTEX-OIZI-0005-SM-2XCED
GTEX-OXRP-0006-SM-2I3FN
GTEX-P4QS-0005-SM-2I3EY
GTEX-P78B-0005-SM-2I5GM
GTEX-PLZ5-0006-SM-5S2W5
GTEX-PLZ6-0006-SM-33HBZ
GTEX-POMQ-0006-SM-5SI7D
GTEX-PSDG-0005-SM-3GADC
GTEX-PVOW-0006-SM-3NMB8
GTEX-PW2O-0006-SM-2I3DV
GTEX-PWCY-0005-SM-33HBP
GTEX-Q2AG-0005-SM-5SI7F
GTEX-Q2AH-0005-SM-33HBR
GTEX-Q2AI-0006-SM-2I3FG
GTEX-QCQG-0006-SM-5SI8M
GTEX-Y5V5-0006-SM-4V6FE
GTEX-Y5V6-0005-SM-4V6FD
GTEX-Y8E4-0006-SM-4V6EW
GTEX-Y8E5-0006-SM-47JWQ
GTEX-Y8LW-0005-SM-4V6EV
GTEX-Y9LG-0006-SM-4VBRK
GTEX-YB5K-0005-SM-4VDSP
GTEX-YBZK-0005-SM-59HKG
GTEX-YFC4-0006-SM-4RGLV
GTEX-ZC5H-0005-SM-4WAXM
GTEX-ZDYS-0002-SM-4WKGR
GTEX-ZE9C-0006-SM-4WKG2
GTEX-ZF29-0006-SM-4WKGQ
GTEX-ZGAY-0006-SM-4WWAQ
GTEX-ZP4G-0006-SM-4WWE6
GTEX-ZPIC-0005-SM-4WWEB
GTEX-ZPU1-0006-SM-4WWAT
GTEX-ZQG8-0005-SM-4YCEH
GTEX-ZQUD-0005-SM-4YCE5
GTEX-ZVE2-0006-SM-51MRW
GTEX-ZVP2-0005-SM-51MRK
GTEX-ZVT2-0005-SM-57WBW
GTEX-ZVZP-0006-SM-51MSW
GTEX-ZXES-0005-SM-57WCB

EBV-transformed lymphocytes (LCLs)

GTEX-1122O-0003-SM-5Q5DL
GTEX-11EM3-0001-SM-5Q5BD
GTEX-11EMC-0002-SM-5Q5DO
GTEX-11OC5-0004-SM-5S2O6
GTEX-11P7K-0003-SM-5S2OU
GTEX-11TT1-0004-SM-5S2NT
GTEX-11VI4-0001-SM-5S2OI
GTEX-1212Z-0002-SM-5SI6W
GTEX-1269C-0003-SM-5S2PB
GTEX-12BJ1-0003-SM-5SI6V
GTEX-12C56-0002-SM-5S2PC
GTEX-RWS6-0001-SM-3NMAL
GTEX-S4Q7-0003-SM-3NM8M
GTEX-S95S-0002-SM-3NM8K
GTEX-SN8G-0001-SM-3NM8L
GTEX-T5JC-0001-SM-3NMAK
GTEX-T5JW-0003-SM-3NMAD
GTEX-T6MN-0002-SM-3NMAH
GTEX-T6MO-0003-SM-3NMAG
GTEX-TKQ1-0003-SM-3NMAE
GTEX-TML8-0001-SM-3NMAF
GTEX-U3ZH-0002-SM-3NMDD
GTEX-U3ZM-0002-SM-3NMDM
GTEX-U3ZN-0002-SM-3NMDF
GTEX-UPJH-0001-SM-3NMDE
GTEX-UPK5-0003-SM-3NMDI
GTEX-V1D1-0003-SM-3NMDP
GTEX-VJYA-0001-SM-3NMDJ
GTEX-VUSG-0003-SM-3NMDK
GTEX-W5WG-0002-SM-3NMDN
GTEX-W5X1-0001-SM-3P61V
GTEX-WFG7-0001-SM-3P61S
GTEX-WFG8-0001-SM-4LVN8
GTEX-WFJO-0002-SM-3P61X
GTEX-WFON-0001-SM-3P61W
GTEX-WHPG-0004-SM-3NMDO
GTEX-WHSB-0002-SM-4M1ZR
GTEX-WOFM-0001-SM-4OOT2
GTEX-WRHK-0001-SM-4WWDD
GTEX-WWTW-0002-SM-4MVNH
GTEX-WXYG-0004-SM-4MVOS
GTEX-WY7C-0004-SM-4ONDS
GTEX-WYVS-0004-SM-4ONDT
GTEX-WZTO-0001-SM-4PQZY
GTEX-X4LF-0002-SM-4QASG
GTEX-X5EB-0004-SM-46MWA
GTEX-XBED-0003-SM-47JWP
GTEX-XBEW-0002-SM-4AT5O
GTEX-XGQ4-0004-SM-4AT5S
GTEX-XMK1-0001-SM-4B64F
GTEX-XPT6-0001-SM-4B64G
GTEX-XQ3S-0001-SM-4B64K
GTEX-XXEK-0004-SM-4BRWO
GTEX-XYKS-0002-SM-4BRWN
GTEX-Y114-0002-SM-4TT78
GTEX-Y3IK-0001-SM-4WWE1
GTEX-Y5LM-0003-SM-4V6G1
GTEX-Y5V5-0001-SM-4V6FZ
GTEX-Y5V6-0003-SM-4V6FX
GTEX-Y8DK-0004-SM-4RGM7
GTEX-Y8E4-0003-SM-4V6FY
GTEX-Y9LG-0001-SM-4VBRQ
GTEX-YB5E-0001-SM-4VDSV
GTEX-YB5K-0003-SM-4VDSN
GTEX-YEC3-0002-SM-4W1YI
GTEX-YEC4-0002-SM-4W1Z6
GTEX-YF7O-0004-SM-4W1ZT
GTEX-YFCO-0003-SM-4W21I
GTEX-ZC5H-0004-SM-4WAXK
GTEX-ZDTS-0001-SM-4WAXW
GTEX-ZDTT-0004-SM-4WKG3
GTEX-ZEX8-0004-SM-4WKFQ
GTEX-ZF29-0002-SM-4WKF2
GTEX-ZF2S-0004-SM-4WKFE
GTEX-ZF3C-0001-SM-4WWAW
GTEX-ZG7Y-0003-SM-4WWEJ
GTEX-ZLWG-0004-SM-4WWD5
GTEX-ZP4G-0003-SM-4WWED
GTEX-ZPIC-0002-SM-4WWEC
GTEX-ZPU1-0004-SM-4WWAV
GTEX-ZQG8-0001-SM-4YCDH
GTEX-ZQUD-0003-SM-4YCD3
GTEX-ZT9W-0003-SM-4YCE6
GTEX-ZT9X-0004-SM-4YCDT
GTEX-ZTPG-0002-SM-4YCEI
GTEX-ZUA1-0002-SM-4YCF7
GTEX-ZV6S-0003-SM-4YCCT
GTEX-ZV7C-0003-SM-4YCF6
GTEX-ZVT2-0001-SM-57WCK
GTEX-ZVTK-0003-SM-51MRV
GTEX-ZVZP-0004-SM-51MS8

Cultured fibroblasts

GTEX-111YS-0008-SM-5Q5BH
GTEX-113JC-0008-SM-5QGR6
GTEX-117XS-0008-SM-5Q5DQ
GTEX-1192W-0008-SM-5QGRE
GTEX-11DXX-0008-SM-5Q5B8
GTEX-11DXY-0008-SM-5QGR4
GTEX-11EMC-0008-SM-5Q5DR
GTEX-11GSP-0008-SM-5Q5DM
GTEX-11I78-0008-SM-5Q5DI
GTEX-11LCK-0008-SM-5Q5BB
GTEX-11NSD-0008-SM-5Q5BC
GTEX-11NUK-0008-SM-5Q5B9
GTEX-11NV4-0008-SM-5Q5BA
GTEX-11O72-0008-SM-5Q5DN
GTEX-11OC5-0008-SM-5S2OH
GTEX-11OF3-0008-SM-5S2NH
GTEX-11ONC-0008-SM-5S2MG
GTEX-11P7K-0008-SM-5S2O5
GTEX-11P81-0008-SM-5S2OT
GTEX-11P82-0008-SM-5S2MS
GTEX-11PRG-0008-SM-5S2N5
GTEX-11TT1-0008-SM-5S2P8
GTEX-11TUW-0008-SM-5SI6S
GTEX-11WQC-0008-SM-5SI6R
GTEX-11WQK-0008-SM-5SI6T
GTEX-11XUK-0008-SM-5S2WD
GTEX-11ZTS-0008-SM-5S2VC
GTEX-11ZTT-0008-SM-5S2TZ
GTEX-11ZUS-0008-SM-5S2UO
GTEX-1211K-0008-SM-5S2W1
GTEX-12126-0008-SM-5S2UC
GTEX-12WSH-0008-SM-5S2V1
GTEX-12WSM-0008-SM-5S2VD
GTEX-1399U-0008-SM-5S2VE
GTEX-N7MS-0008-SM-4E3JI
GTEX-NFK9-0008-SM-4E3JE
GTEX-NL3G-0008-SM-4E3JX
GTEX-O5YT-0008-SM-4E3IQ
GTEX-O5YW-0008-SM-4E3IE
GTEX-OHPK-0008-SM-4E3JL
GTEX-OHPL-0008-SM-4E3I9
GTEX-OHPM-0008-SM-4E3IP
GTEX-OHPN-0008-SM-4E3HW
GTEX-OIZG-0008-SM-4E3J2
GTEX-OIZI-0008-SM-2XCFD
GTEX-OOBJ-0008-SM-3NB26
GTEX-OOBK-0008-SM-3NB27
GTEX-OXRK-0008-SM-3NB28
GTEX-T2IS-0008-SM-4DM75
GTEX-T5JC-0008-SM-4DM6A
GTEX-U4B1-0008-SM-4DXUW
GTEX-U8T8-0008-SM-4DXSP
GTEX-UJHI-0008-SM-4IHL1
GTEX-UJMC-0008-SM-4IHKK
GTEX-UPK5-0008-SM-4IHJD
GTEX-V1D1-0008-SM-4JBIJ
GTEX-W5X1-0008-SM-4LMKA
GTEX-WFG7-0008-SM-4LMKB
GTEX-WHPG-0008-SM-4M1ZQ
GTEX-WHSB-0008-SM-4M1ZP
GTEX-WHWD-0008-SM-4OOSU
GTEX-WI4N-0008-SM-4OOSV
GTEX-WL46-0008-SM-4OOSW
GTEX-WQUQ-0008-SM-4OOT1
GTEX-WRHU-0008-SM-4MVPB
GTEX-WVJS-0008-SM-4MVPC
GTEX-WVLH-0008-SM-4MVPD
GTEX-WY7C-0008-SM-4ONDW
GTEX-WYBS-0008-SM-4ONDX
GTEX-WYJK-0008-SM-4ONDV
GTEX-WYVS-0008-SM-4ONDY
GTEX-WZTO-0008-SM-4PQZZ
GTEX-X15G-0008-SM-4PR2D
GTEX-X3Y1-0008-SM-4PR12
GTEX-X4LF-0008-SM-4QAST
GTEX-XBEC-0008-SM-4AT3X
GTEX-XBEW-0008-SM-4AT3Y
GTEX-XMD2-0008-SM-4WWE7
GTEX-XMD3-0008-SM-4AT4V
GTEX-XMK1-0008-SM-4GICF
GTEX-XOT4-0008-SM-4B664
GTEX-XPT6-0008-SM-4B64Q
GTEX-XPVG-0008-SM-4GICH
GTEX-XQ3S-0008-SM-4GIDZ
GTEX-XUW1-0008-SM-4BOQH
GTEX-XV7Q-0008-SM-4BRWL
GTEX-Y8E4-0008-SM-4V6FW
GTEX-Y9LG-0008-SM-4VBRJ
GTEX-YB5K-0008-SM-4VDT8
GTEX-YEC4-0008-SM-4W1YR
GTEX-YF7O-0008-SM-4W1ZS
GTEX-YJ89-0008-SM-4RGM4
GTEX-Z93S-0008-SM-4RGM5
GTEX-ZC5H-0008-SM-4WAX8
GTEX-ZDTS-0008-SM-4E3I8
GTEX-ZDTT-0008-SM-4E3K5

GTEX-OXRL-0008-SM-3NB29
GTEX-P4PP-0008-SM-48TDV
GTEX-P4QT-0008-SM-48TDZ
GTEX-PSDG-0008-SM-48TE5
GTEX-PW2O-0008-SM-48TEB
GTEX-PWCY-0008-SM-48TE9
GTEX-PX3G-0008-SM-48U2L
GTEX-Q2AH-0008-SM-48U2J
GTEX-QCQG-0008-SM-48U2G
GTEX-QLQ7-0008-SM-447AW
GTEX-QXCU-0008-SM-48FCH
GTEX-R45C-0008-SM-48FF2
GTEX-R55C-0008-SM-48FCF
GTEX-R55D-0008-SM-48FEV
GTEX-R55E-0008-SM-48FCG
GTEX-R55G-0008-SM-48FEX
GTEX-RM2N-0008-SM-48FF3
GTEX-RN64-0008-SM-48FEZ
GTEX-RNOR-0008-SM-48FEY
GTEX-RU1J-0008-SM-46MV9
GTEX-RU72-0008-SM-46MV8
GTEX-RWS6-0008-SM-47JYV
GTEX-RWSA-0008-SM-47JYX
GTEX-S33H-0008-SM-4AD6C
GTEX-S4Z8-0008-SM-33HAZ
GTEX-SE5C-0008-SM-4B64J
GTEX-SJXC-0008-SM-4DM7G
GTEX-ZDXO-0008-SM-4E3HR
GTEX-ZDYS-0008-SM-4E3IX
GTEX-ZE7O-0008-SM-4E3JQ
GTEX-ZEX8-0008-SM-4E3JU
GTEX-ZF2S-0008-SM-4E3IK
GTEX-ZF3C-0008-SM-4E3IL
GTEX-ZLWG-0008-SM-4E3J4
GTEX-ZP4G-0008-SM-4E3I4
GTEX-ZPIC-0008-SM-4E3JF
GTEX-ZPU1-0008-SM-4E3IR
GTEX-ZQG8-0008-SM-4E3J9
GTEX-ZQUD-0008-SM-4YCCU
GTEX-ZT9W-0008-SM-4YCDJ
GTEX-ZT9X-0008-SM-4YCD7
GTEX-ZTPG-0008-SM-4YCEK
GTEX-ZTX8-0008-SM-4YCDV
GTEX-ZUA1-0008-SM-4YCEW
GTEX-ZV68-0008-SM-4YCCV
GTEX-ZV6S-0008-SM-4YCF9
GTEX-ZV7C-0008-SM-57WCL
GTEX-ZVE2-0008-SM-51MRU
GTEX-ZVP2-0008-SM-51MSL
GTEX-ZVT2-0008-SM-57WC9
GTEX-ZVT3-0008-SM-51MRI
GTEX-ZVTK-0008-SM-57WDA
GTEX-ZVZP-0008-SM-51MSX
GTEX-ZXES-0008-SM-57WCX

Supplementary Results

Minimum required sequencing depth (MRSD) scores differ across biosamples

For all but one parameter combination, moving from $MRSD_{0.95}$ to $MRSD_{0.99}$ resulted in an increase in median MRSD of between 26.19-155.40%. However, when stipulating 95% splice junction coverage for skeletal muscle samples, we observed a decrease of 4.66% in MRSD scores for $MRSD_{0.95}$ (*n* =1323, median = 42.52) compared to $MRSD_{0.99}$ (*n* = 973, median = 40.54); this was accounted for by an increase in the number of genes that were considered “unfeasible” for surveillance, i.e. those for which zero reads cover the given proportion of junctions (*n* unfeasible $MRSD_{0.95}$ = 1873, *n* unfeasible $MRSD_{0.99}$ = 2193). This definition of feasibility is limited by the sequencing depth of the reference sets on which the predictions are based. Ultra-deep sequencing of the same reference sets, may have enabled feasible MRSD predictions for an increased number of splicing junctions.

Impact of read length on MRSD accuracy

To assess whether the MRSD scores themselves were altered through derivation from 75 bp or 150 bp RNA-seq reference sets, we generated paired MRSD scores from datasets that were trimmed from 150 bp to 75 bp reads (Figure S7). We were able to calculate MRSD scores for 54.2% of multi-exon disease-associated genes (1802/3322) from these datasets. 86.5% (243/1802) of observable genes had lower MRSD scores from 150 bp read reference sets than from 75 bp read reference sets, or were only feasible in 150 bp reference sets. 13.5% (243/1802) counter-intuitively exhibited a higher MRSD in the 150 bp dataset, suggesting that fewer 75 bp reads were required to adequately cover these transcripts. In many examples, this could be attributed to a decrease in mapping quality of longer reads such that the reads did

not pass the quality filters of the employed pipeline[13]. Further work is needed to ascertain whether this discarding of longer reads is a harmful artefact of the filtering process, or a genuine removal of uninformative reads.

Comparison of MRSD and TPM as a guide for appropriate surveillance

We noted significant overlap between genes grouped into low-MRSD (< 100 M reads) and high-MRSD (≥ 100 M reads) brackets. For example, among genes considered low-MRSD, TPM values ranged from 0.99-246,600, while genes with high-MRSD values had TPM values between 0.20-8644 (Figure 3D). We quantified the overlap between these distributions, demonstrating that, depending on the tissue, between 98.0% and 99.3% of high-MRSD genes had higher TPM values than at least one low-MRSD gene. We also observed that, in their respective tissues, the TPMs of 44.1-60.0%, 8.5-16.7% and 3.4-6.6% of high-MRSD genes exceeded those of the 5%, 30% and 50% least-expressed low-MRSD genes, respectively (Figure 3D). The substantial overlap in the TPM values for low and high MRSD genes suggests that relative expression does not provide a wholly accurate representation of transcript coverage in RNA-seq data. Such inconsistencies may arise from bias in the regions of genes that are sequenced, for example, genes with high degrees of 3' bias in RNA-seq datasets or significant alternative transcript usage (Figure S8).

Factors influencing the likelihood of pathogenic splicing variation identification & MRSD predictions

To further define the most informative parameters for use in the MRSD model, we investigated the impact of a variety of metrics on the capability to identify pathogenic splicing events, including number of samples within the healthy reference set, the degree of read support for splicing junctions, and the relative expression of genes of interest. We aimed to quantify the effect of changes in these metrics on both the total number of events of interest and the position within the list of events (see Materials and Methods for filtering and ranking strategy).

We first identified how the number of control samples used as a reference set for “healthy splicing” impacted our ability to identify aberrant splicing events. For all samples within our healthy splicing set, we iteratively selected groups of control samples at sizes of 30, 60 or 90. We observed that moving from 30 to 60 controls is associated with a mean reduction in event count of 19.3% (28.1% of non-singleton events, 17.1% of singleton events) across the three tissues, while increasing the control size to 90 results in a further reduction of 10.2% of events (16.5% of non-singleton events, 9.5% of singleton events; Figure 4); this effect was consistent across tissue types.

We next investigated how read count filters impacted the number of events observed for a given individual (Figure 4). Filtering out all splicing events supported by just a single read against a background of 90 control samples removes, on average, 91.2% of events (60.4% of non-singleton events, 97.3% of singleton events). Increasing read support thresholds to 10 unique sequencing reads results in a total of 99.4% of

events being excluded on average (96.2% of non-singleton events, 99.99% of singleton events), while retaining only those events supported by 100 reads or more removes an average of 99.97% of events (99.8% of non-singleton events, 100.0% of singleton events). To understand how the level of read support impacted the ability to identify specific events, we collated 31 aberrant splicing events across 22 muscle-derived RNA-seq samples, and downsampled reads in the genes containing these events. We observed that we could identify the same aberrant splicing events at reduced relative expression levels, and, while read support decreased (Figure 5A), the ranked position of the event within the rank-ordered output remained approximately the same in most cases (Figure 5B). However, the weakened read support increased the risk of eliminating the variant from consideration when read count filters were applied (Figure 5C). This analysis further emphasized that TPM values alone may not be a reliable measure of ability to survey all splicing junctions within a gene; we observed that splice junctions in different samples covered by the same number of sequencing reads belonged to genes with widely ranging TPM values (Figure S10). For example, splice junctions covered by eight reads were identified in genes with TPMs ranging between 0.17 and 52.

Based on these investigations, we selected an eight-read coverage value for downstream analyses; as we observed that the majority of pathogenic mis-splicing events have an NRC ≥ 0.25, stipulating an eight-read coverage requirement means that aberrant events should be covered by at least two reads, and so be retained when filtering single-read events from the list of splicing events. We appreciate that the use of more stringent parameters may be preferable in some use cases, such as to generate sufficient corroboration to support the reporting of a diagnostic finding to

a patient or when using significance-based tools such as FRASER, LeafCutterMD and SPOT. However, our investigations have shown this approach to be robust for the initial highlighting of aberrant splicing events for downstream analysis.

References

1. Cummings, B.B., Marshall, J.L., Tukiainen, T., Lek, M., Donkervoort, S., Foley, A.R., et al. Improving genetic diagnosis in Mendelian disease with transcriptome sequencing. (2017). Sci Transl Med. *9(386)*.
2. The GTEx Consortium. (2013). The Genotype-Tissue Expression (GTEx) project. Nat Genet. *45*(6), 580-585.
3. Dobin, A., Davis, C.A., Schlesinger, F., Drenkow, J., Zaleski, C., Jha, S., Batut, P., Chaisson, M., and Gingeras, T.R. (2013). STAR: ultrafast universal RNA-seq aligner. Bioinformatics. *29(1)*, 15-21.
4. Jaganathan, K., Kyriazopoulou Panagiotopoulou, S., McRae, J.F., Darbandi, S.F., Knowles, D., Li, Y.I., et al. (2019) Predicting Splicing from Primary Sequence with Deep Learning. Cell. *176(3)*:535-48.e24.
