# Supplementary material for: MRSD: A quantitative approach for assessing suitability of RNA-seq in the investigation of mis-splicing in Mendelian disease
Source: Am J Hum Genet. 2022 Jan 21;109(2):210–22. doi: 10.1016/j.ajhg.2021.12.014 (PMC8874219; doi:10.1016/j.ajhg.2021.12.014)
Supplement: Document S2. Article plus supplemental information [file mmc2.pdf]

MRSD: A quantitative approach for assessing suitability of RNA-seq in the investigation of mis-splicing in Mendelian disease

Authors

Charlie F. Rowlands, Algy Taylor, Gillian Rice, ...,
Diana Baralle, Tracy A. Briggs, Jamie M. Ellingford

Correspondence

jamie.ellingford@manchester.ac.uk

Rowlands et al., 2022, The American Journal of Human Genetics *109*, 210–222
February 3, 2022 © 2022 The Authors.
https://doi.org/10.1016/j.ajhg.2021.12.014

MRSD: A quantitative approach for assessing suitability of RNA-seq in the investigation of mis-splicing in Mendelian disease

Charlie F. Rowlands,[1,2] Algy Taylor,[2] Gillian Rice,[1] Nicola Whiffin,[3] Hildegard Nikki Hall,[4] William G. Newman,[1,2] Graeme C.M. Black,[1,2] kConFab Investigators,[5,6] Raymond T. O'Keefe,[1] Simon Hubbard,[1] Andrew G.L. Douglas,[7,8] Diana Baralle,[7,8] Tracy A. Briggs,[1,2] and Jamie M. Ellingford[1,2,*]

Abstract

Variable levels of gene expression between tissues complicates the use of RNA sequencing of patient biosamples to delineate the impact of genomic variants. Here, we describe a gene- and tissue-specific metric to inform the feasibility of RNA sequencing. This overcomes limitations of using expression values alone as a metric to predict RNA-sequencing utility. We have derived a metric, minimum required sequencing depth (MRSD), that estimates the depth of sequencing required from RNA sequencing to achieve user-specified sequencing coverage of a gene, transcript, or group of genes. We applied MRSD across four human biosamples: whole blood, lymphoblastoid cell lines (LCLs), skeletal muscle, and cultured fibroblasts. MRSD has high precision (90.1%–98.2%) and overcomes transcript region-specific sequencing biases. Applying MRSD scoring to established disease gene panels shows that fibroblasts, of these four biosamples, are the optimum source of RNA for 63.1% of gene panels. Using this approach, up to 67.8% of the variants of uncertain significance in ClinVar that are predicted to impact splicing could be assayed by RNA sequencing in at least one of the biosamples. We demonstrate the utility and benefits of MRSD as a metric to inform functional assessment of splicing aberrations, in particular in the context of Mendelian genetic disorders to improve diagnostic yield.

Introduction

Pinpointing disease-causing genomic variation informs diagnosis, treatment, and management for a wide range of rare disorders. Pathogenic variants, both protein-coding and intronic, that lie outside canonical splice sites may nonetheless act to disrupt pre-mRNA splicing through a diverse series of mechanisms (Figure S1).[1–3] Effective identification of pathogenic splice-impacting variants remains challenging and is limited by the omission of intronic regions in targeted sequencing approaches,[4,5] discordance between *in silico* variant prioritization tools,[6] and the lack of availability of the appropriate tissue from which to survey RNA for splicing disruption.[7,8]

Targeted analyses such as RT-PCR enable detection of splicing aberrations[3] but are designed to test for the presence of specific disruptions. As such they may not identify the complete spectrum of splicing disruption caused by a single genomic variant. By contrast, RNA sequencing (RNA-seq) offers a potential route to identify aberrant splicing events without prior knowledge of the underlying genomic variants driving their impact.[3,9–13] Further, there is growing evidence that RNA-seq can substantially improve diagnostic yield across a variety of disease subtypes[3,10,13–15] through identification of variants impacting splicing or leading to impairment of transcript expression or stability.[16]

However, there remain several hurdles to the effective and routine integration of RNA-seq into diagnostic pipelines. For example, surveying a whole transcriptome identifies a large number of splicing events—in the order of hundreds of thousands. Despite a recent increase in the number of tools designed to scrutinize RNA-seq data for splicing outliers,[9,13,17,18] there is little consensus regarding the best approach to filter true positive and pathogenic events from neutral or artifactual findings. Furthermore, diagnostic analysis using RNA-seq is only effective when sufficient levels of sequence coverage of a relevant gene transcript are present in the sampled tissue.

In this study, we develop an informatics approach to assess the suitability of RNA-seq derived from different tissues to identify pathogenic splicing aberrations in specific genes of interest (Figure S2). We name our framework the minimum required sequencing depth (MRSD), which can be utilized in a flexible and customized manner

[1]Division of Evolution, Infection and Genomic Sciences, School of Biological Sciences, Faculty of Biology, Medicine and Health, University of Manchester, Manchester M13 9PT, UK; [2]Manchester Centre for Genomic Medicine, St Mary's Hospital, Manchester University NHS Foundation Trust, Manchester Academic Health Science Centre, Manchester M13 9WL, UK; [3]Wellcome Centre for Human Genetics, University of Oxford, Oxford OX3 7BN, UK; [4]MRC Human Genetics Unit, Institute of Genetics and Cancer, University of Edinburgh, Edinburgh EH4 2XU, UK; [5]Sir Peter MacCallum Department of Oncology, The University of Melbourne, Parkville, VIC 3010, Australia; [6]kConFab, Research Department, Peter MacCallum Cancer Centre, Melbourne, VIC 3000, Australia; [7]Wessex Clinical Genetics Service, Princess Anne Hospital, University Hospital Southampton NHS Foundation Trust, Coxford Rd, Southampton SO16 5YA, UK; [8]Faculty of Medicine, University of Southampton, Duthie Building, Southampton General Hospital, Tremona Road, Southampton SO16 6YD, UK
*Correspondence: jamie.ellingford@manchester.ac.uk
https://doi.org/10.1016/j.ajhg.2021.12.014.
© 2022 The Authors. This is an open access article under the CC BY license (http://creativecommons.org/licenses/by/4.0/).

(Figure S2). MRSD scores (see web resources for access) can be utilized to select the most appropriate biosample to detect specific splicing aberrations and to guide required depth of sequencing.

Material and methods

Minimum required sequencing depth (MRSD) score

The MRSD model considers the level of sequencing coverage for splice junctions in tissue-specific reference sets (see Reference set generation from control RNA-seq data) and calculates the minimum required sequencing depth, in millions of uniquely mapping 75 bp reads, that would be required for the desired proportion of splice junctions in a given transcript to be covered by a desired number of sequencing reads. The model is dynamic and can be adjusted by the user to account for customized levels of desired sequencing coverage per splicing junction, the proportion of splicing junctions covered, and the "MRSD parameter" ($_m$) which represents the proportion of control samples for which the returned MRSD holds true (suggested usage of 0.95 or 0.99).

MRSD is defined for an individual transcript in a given sample as:

$$MRSD_m = r \bigg/ \left(\frac{R_p}{d}\right)$$

where r is the desired level of read coverage across desired proportion p of splice junctions, R is the set of read counts supporting each of the splice junctions in the transcript of interest, ordered from lowest to highest, and R_p is the read count at the position in R at which proportion p of read counts values in R are greater than or equal to it. d represents the total number of sequencing reads, in millions of reads, in the RNA-seq sample (by default, the number of uniquely mapping sequencing reads), and (m) represents the MRSD parameter. Where there is zero-read coverage of the critical number of splice junctions (i.e., where $R_p = 0$), no MRSD can be generated and surveying of the transcript is deemed "unfeasible" in the given tissue. Further elaboration and an illustrative example are given in supplemental material and methods S1.

Hierarchical approach to transcript selection and investigation of impact of transcript selection on MRSD predictions

MRSD can be calculated for any transcript sets of interest. For the analyses described in this study, we generated a single transcript model for each gene in the GENCODE v19 human genome annotation (supplemental material and methods S2). We utilized a hierarchical approach for transcript selection, whereby we prioritized transcripts in the MANE v.0.7 curated transcript list, providing that all splicing junctions for a given transcript were supported in the GENCODE v.19 annotation. Genes without MANE transcripts were assigned composite transcripts, consisting of the union of all junctions found in transcripts for the given gene in NCBI RefSeq. For genes lacking both a corresponding MANE and RefSeq transcript, the union of all junctions present in all GENCODE v.19-listed transcripts for that gene were used as the transcript model.

To investigate the suitability of our hierarchical transcript selection approach and the stability of MRSD scores across transcripts, we also generated MRSD scores for all transcripts listed in the GENCODE v.19 annotation, using default MRSD parameters. MRSD scores for transcripts selected through the hierarchical approach were stratified according to whether they were classified as unfeasible or feasible and compared against the transcript-level MRSD predictions for all transcripts available in GENCODE for the given gene.

Ethics approval and consent to participate

External datasets utilized in this study were accessed under dbGaP project accessions phs000655.v3.p1.c1 and phs000424.v8.p2. Informed written consent was obtained for all in-house analyses, with ethical and study approval from South Central-Hampshire A (ref: 17/SC/0026), South Central-Oxford B (ref:11/SC/0269), South Manchester (ref:11/H10003/3), and Scotland A (refs: 06/MRE00/76 and 16/SS/0201) Research Ethics Committees.

Reference set generation from control RNA-seq data

FASTQs were downloaded from the Database of Genotypes and Phenotypes (dbGaP) under the project accessions phs000424.v8.p2 and phs000655.v3.p1.c1 for GTEx control individuals and neuromuscular disease-affected individuals, respectively. GTEx controls were selected for LCLs (n = 91), skeletal muscle (n = 184), whole blood (n = 150), and cultured fibroblasts (n = 150) according to tissue-specific criteria (supplemental material and methods S3) to ensure use of only high-quality samples in generating control splicing datasets. A collated map of splice junction coverage was generated for our defined transcripts (see Hierarchical approach to transcript selection) from these control datasets using established methods.[13] These samples and their associated splice junction usage were designated as reference sets.

In-house RNA-seq generation

We evaluated the accuracy of MRSD using independently derived RNA-seq samples from the reference sets which generated the model. The positive predictive value (PPV) was defined as the proportion of transcripts where the obtained sequencing depth for splicing junctions exceeded or equaled the MRSD prediction. Conversely, the negative predictive value (NPV) was defined as the proportion of transcripts where appropriate sequencing coverage was not obtained according to the MRSD parameters applied.

The RNA-seq datasets utilized in these analyses were accessed from previously published datasets[13] (dbGaP project accession phs000655.v3.p1.c1), through international consortia,[19] or from individuals in whom written informed consent was obtained and ethical approval for the study granted by Scotland A (refs: 06/MRE00/76 and 16/SS/0201), South Central-Hampshire A (ref: 17/SC/0026), South Central-Oxford B (ref:11/SC/0269), or South Manchester (ref: 11/H10003/3) Research Ethics Committee.

For in-house peripheral blood samples, RNA was extracted from PAXgene Blood RNA Kits and underwent poly-A enrichment library preparation using the TruSeq Stranded mRNA assay (Illumina) followed by 76 bp paired end sequencing using an Illumina HiSeq 4000 sequencing platform. For in-house LCL samples, RNA was extracted from pelleted LCLs thawed directly into TRIzol reagent (Invitrogen, 15596-026) using chloroform and treated with TURBO DNase (Invitrogen, AM1907), following the manufacturers' instructions. RNA was prepared using the NEBNEXT Ultra II Directional RNA Library Prep kit (NEB #7760) with the Poly-A mRNA magnetic isolation module (NEB #E7490), according to manufacturer's instructions, and 75 bp paired end sequencing was performed using the Illumina NextSeq 550 sequencing platform. Ribosomal RNA-depleted datasets were generated using RNA extracted via the PAXgene Blood RNA system, and 150 bp paired end sequencing

Box 1. Metrics collated during splice event analysis

Read count: Number of split reads supporting the existence of a given splice junction
Normalized read count (NRC): Ratio of the number of reads supporting a given junction to the numbers of reads supporting adjoining canonical junction with the highest supporting read count
NRC fold change: fold difference in NRC for a given event between an individual and the control individual with the next-highest NRC for that event
Number of samples: the number of individuals, across both case and controls, in which an event is present
Rank: position of a given event in a list of significant events, when ordered by decreasing read count (for singleton events) or fold change (for non-singleton events)

performed via Novogene (Hong Kong) using the NEBNext Globin and rRNA Depletion and NEBNext Ultra Directional RNA Library Prep Kits on a HiSeq 2000 instrument (Illumina). RNA samples from 20 LCLs were obtained from the kConFab consortium. Poly(A)-selected RNA was generated using the TruSeq Stranded mRNA Library Prep Kit (Illumina), and 150 bp paired end reads created using the NextSeq 500 instrument (Illumina).

Splice event identification

All FASTQs were aligned and processed as previously described.[13] Briefly, this analysis consisted of two-pass alignment using STAR[20] (v.2.4.2), marking of suspected PCR duplicates, and processing of the resulting alignments to generate tissue-by-tissue lists of read support counts for splice junctions present within the samples in the cohort. Metrics for each splicing event were collected (Box 1), and splicing junctions were filtered to retain only those events that were unique to single samples (singletons) or that were present in multiple samples (non-singletons) but with an increased usage in the sample of interest, i.e., a higher normalized read count (NRC) than any control in the reference set. The resulting list of splice events was ranked according to NRC fold change, with singletons with high read counts considered the most significant events.

Factors influencing the likelihood of aberrant splicing identification

To calculate how the level of background splicing aberrations was altered by sample size, each individual in three of the four reference sets was processed using the above pipeline[13] and compared against 2,000 bootstraps of 30, 60, and 90 control subjects each from their respective control tissue dataset with replacement. Events were then filtered to retain only those events for which the NRC was higher in the given individual than in any controls. Median counts for singleton and non-singleton events were collated for each control group size.

To understand the impact of splicing junction coverage on the ability to retain events of interest, we selected 31 splicing events identified in neuromuscular patient RNA-seq data that were either unique to or had increased NRC in comparison to the tissue-specific reference set. For these individuals, we removed random subsets of reads in 10% intervals from each of the genes containing these events. The resulting datasets mimicked variable expression of a single gene in these samples and were subsequently analyzed using the splice analysis pipeline.[13]

Genomics England PanelApp data collection

Tabulated versions of 295 gene panels were downloaded from the Genomics England PanelApp repository on June 28, 2021. Each panel was filtered to retain only multiexon genes assigned a "green" classification, representing the highest level of confidence of a real genotype-phenotype association. This yielded 3,322 unique genes for downstream analysis.

Curation of ClinVar variants of uncertain significance

A tabulated version of the comprehensive ClinVar variant listing[21] for January 2021 was downloaded and filtered to retain only those variants that were annotated as either "uncertain significance" or "conflicting interpretations of pathogenicity." SpliceAI scores[22] (v.1.2.1) were generated for these variants and those with a score of 0.5 or greater retained for downstream analysis.

Results

Minimum required sequencing depth (MRSD) scores differ across biosamples

We curated a list of 3,322 multi-exon disease-related genes and defined a single transcript for each gene using our hierarchical approach (see material and methods). MRSD scores were generated for these transcripts using GTEx samples for four clinically relevant tissues to create tissue-specific reference sets (Figure S2): whole blood (n = 150), LCLs (n = 91), skeletal muscle (n = 184), and cultured fibroblasts (n = 150). MRSD scores for these reference sets are available (see web resources).

Three parameters can be altered for the MRSD model (desired read coverage, percentage of splice junctions, and the MRSD parameter). We observed that the MRSD score differed dependent on the values chosen for these parameters (Figure 1). For example, when specifying a desired read coverage level of eight reads per splicing junction, we observed that increases in the desired proportion of covered splice junctions from 75% to 95% was associated with an increase in median MRSD of between 0.27% (in skeletal muscle, $MRSD_{0.99}$) and 55.95% (in LCLs, $MRSD_{0.95}$; Figure 1B, top). For all but one parameter combination, moving from $MRSD_{0.95}$ to $MRSD_{0.99}$ resulted in an increase in median MRSD of between 26.19% and 155.40% (Figure 1; supplemental results).

Overall, our analyses suggested that, of the four investigated biosamples, fibroblasts enable investigation of the most comprehensive set of genes for aberrant splicing. Although LCLs displayed the lowest median MRSDs across

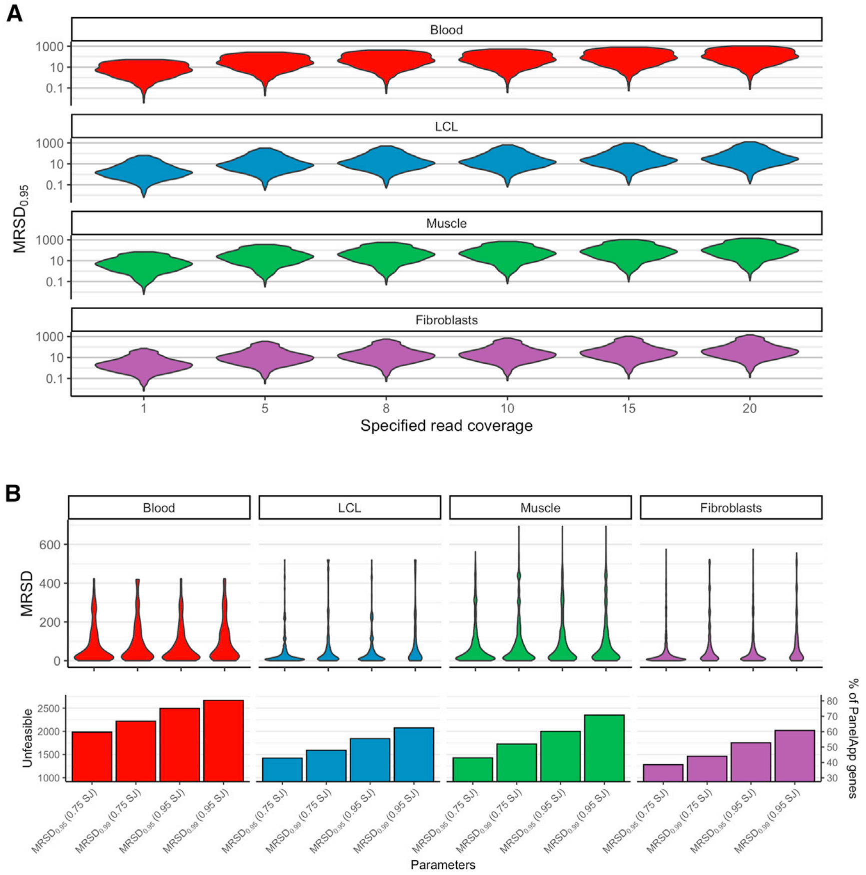

Figure 1. Minimum required sequencing depth (MRSD) predictions vary with changes in model parameters and across tissues

(A) When all other parameters are constant (default parameters used here), increasing the desired level of read coverage of a gene results in a proportional increase in MRSD.

(B) Top: In most cases, for a given level of splice junction (SJ) coverage, increasing the desired MRSD parameter (the proportion of RNA-seq runs for which the MRSD prediction is expected to be sufficient) results in an increase in median MRSD score. Bottom: The number of genes predicted to be unfeasible for analysis increases gradually as parameter stringency increases. At the highest level of stringency, the specified coverage was predicted unfeasible for between 62.5% (2,076/3,322, in LCLs) and 80.3% (2,668/3,322, in blood) of PanelApp genes.

all parameter combinations (range = 12.86–33.77, Figure 1B, top), the difference in median MRSDs compared to fibroblasts was small (range = 14.44–35.06) and a greater number of genes were predicted "unfeasible" for analysis (see material and methods) in LCLs than in fibroblasts (42.8%–62.5% versus 38.6%–60.7% of PanelApp genes, respectively). Whole blood exhibited the highest number of unfeasible genes across the different parameter combinations (59.7%–80.3%).

Accuracy of minimum required sequencing depth (MRSD) calculations

In order to assess the performance of the MRSD model across a variety of parameter combinations, we obtained independent RNA-seq datasets for 68 samples for three of the four investigated tissues (blood, n = 12; LCLs, n = 4; muscle, n = 52), with a wide range of sequencing depths (Figure S3). All data utilized in this analysis were generated through 75 bp paired end sequencing. We observed 96% PPV and 79% NPV, on average, for the 68 samples (Figure 2A). We observed a general trend that the PPV and NPV of MRSD decreased and increased, respectively, at higher levels of required coverage (Figures 2B and 2C). Across all parameter combinations, PPVs ranged from 90.1% to 98.2%, while NPVs ranged from 56.4% to 94.7%, suggesting MRSD is a conservative model that primarily returns positive results with high certainty.

Investigation of inter-transcript MRSD variability

We generated MRSD scores for all possible transcripts available in the GENCODE v.19 annotation (n = 20,188 genes with >1 transcript) and observed an overall median relative variability (coefficient of variation, CV_{MRSD}) of 0.37–0.49 across the surveyed genes, depending on the tissue (Figure S4A). Where differences in MRSD predictions were observed, there was a median difference in MRSD of 1.06–3.65 M reads between our selected transcripts and the transcript with the lowest predicted MRSD for each gene (Figure S4B).

Further, in 95.10%–95.37%, of genes where automatically selected transcripts were classed as unfeasible, and in 89.05%–90.37% of multi-transcript genes classed as unfeasible, we observed that all transcripts in the GENCODE v19 dataset were also classified as unfeasible (Figure S5A). We observed an average minimum MRSD score of 108.59–157.78 M reads, dependent on tissue, for the small number of genes that displayed discordance in feasibility predictions between GENCODE v.19 and the automatically selected transcript (Figure S5B). These data illustrate a general trend of low variability in MRSD scores for genes with multiple possible transcripts, but importantly demonstrate that individual transcript selection may yield different MRSD scores in some contexts and thereby influence decisions on accessibility.

Impact of read length on MRSD accuracy

To understand the impact of longer sequencing reads on MRSD accuracy, we evaluated the ability of the model to predict transcript coverage for independently derived 150 bp paired-end RNA-seq data (LCLs, n = 20). We observed higher median PPVs across samples for 150 bp

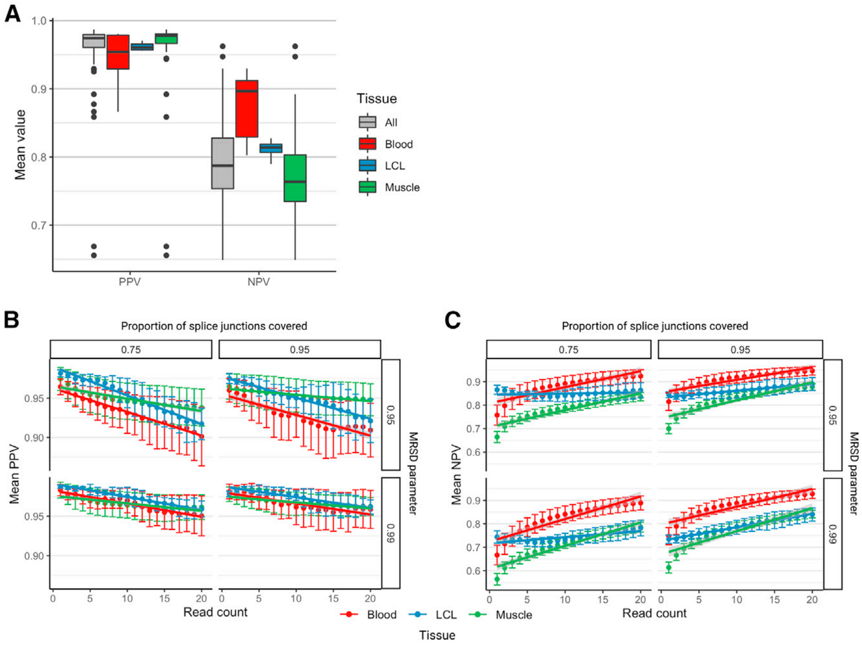

Figure 2. Performance metrics of the MRSD model
The ability of MRSD to accurately predict levels of PanelApp disease gene coverage based on sequencing depth was tested on unseen RNA-seq datasets from blood (n = 12), LCLs (n = 4), and muscle (n = 52).
(A) The mean positive predictive values (PPVs) and negative predictive values (NPVs) averaged across all parameter combinations for each RNA-seq dataset show that the median PPV is slightly lower, and the median NPV slightly higher, for whole blood than for LCLs and skeletal muscle.
(B and C) Breakdown of (B) PPVs and (C) NPVs for the MRSD model by parameters shows that specifying an increasing desired read coverage results in a gradual decrease in PPV and increase in NPV across all tissues and parameter combinations. Dependent on parameter stringency and limiting analysis to a maximum specification of 20-read coverage, PPV predictions range from 90.1% to 98.2%, while NPV ranges from 56.4% to 94.7%. Error bars show 95% confidence interval.

datasets than with 75 bp datasets for half of the four parameter combinations tested (Figure S6). NPVs were slightly lower for 150 bp datasets for all combinations of parameters (Figure S6). While MRSD scores should ideally be applied to datasets generated using the same experimental approach, these data suggest that they are widely applicable to datasets generated through an alternative manner.

We also observed through a paired analysis of 150 bp and 75 bp datasets that 86.5% (1,559/1,802) of multi-exon disease genes that could be surveyed from LCLs either had lower MRSD scores from 150 bp read reference sets than from 75 bp read reference sets, or were only predicted to be feasible for surveillance from 150 bp reference sets (Figure S7; supplemental results). This further emphasizes the advantages of longer RNA-seq reads.

Comparison of MRSD and TPM as a guide for appropriate surveillance

We compared MRSD to the use of relative expression level (in transcripts per million, TPM) as a possible indicator of RNA-seq suitability for the detection of aberrant splicing events. We identified a negative correlation between the level of gene expression and its predicted MRSD across all four tissues ($r^2 = 0.613$–0.714; Figures 3A–3D). This confirms that more highly expressed genes are associated with lower MRSD scores. However, we noted significant overlap between genes grouped into low-MRSD (<100 M reads) and high-MRSD (≥100 M reads) brackets (Figure 3D; supplemental results), suggesting that relative expression does not provide a wholly accurate representation of complete transcript coverage in RNA-seq data. Such inconsistencies may arise from bias in the regions of genes that are sequenced, for example, genes with high degrees of 3′ bias in RNA-seq datasets or significant alterations in isoform usage between tissues (Figure S8).

Traits of pathogenic splicing variation vary widely between genes and events

We next aimed to determine the optimal MRSD parameters for detection of aberrant splicing through the investigation of 21 RNA-seq samples from patients harboring pathogenic mis-splicing events (Table S1; Figure S9). We observed high variability in indicative metrics associated with pathogenic aberrant splicing events using a previously published bioinformatics pipeline[13] (Table 1). All pathogenic events identified through RNA-seq were supported by two or more reads and with normalized read counts (NRCs) ≥ 0.19. 90% of the known pathogenic events would be retained if filtering for events that were supported by 2 or more reads, and events that were singletons (evident only in a single sample) or non-singletons with an NRC > 0.25 (Table 1).

We also investigated the ability of three recent splice prediction tools to identify the 21 pathogenic mis-splicing events, specifically FRASER,[9] SPOT,[17] and LeafCutterMD.[18] We observed variability in the events that were identified by these tools (Table 1). FRASER identified 81% (17/21) of pathogenic mis-splicing events, with 16 of these flagged as statistically significant splicing outliers ($p < 0.05$), including events supported by 3 or more sequencing reads.

Factors influencing the likelihood of pathogenic splicing variation identification & MRSD predictions

We next investigated the impact of varying input metrics on the ability to successfully identify pathogenic splicing events. This includes number of samples within the reference set, degree of read support for splicing junctions,

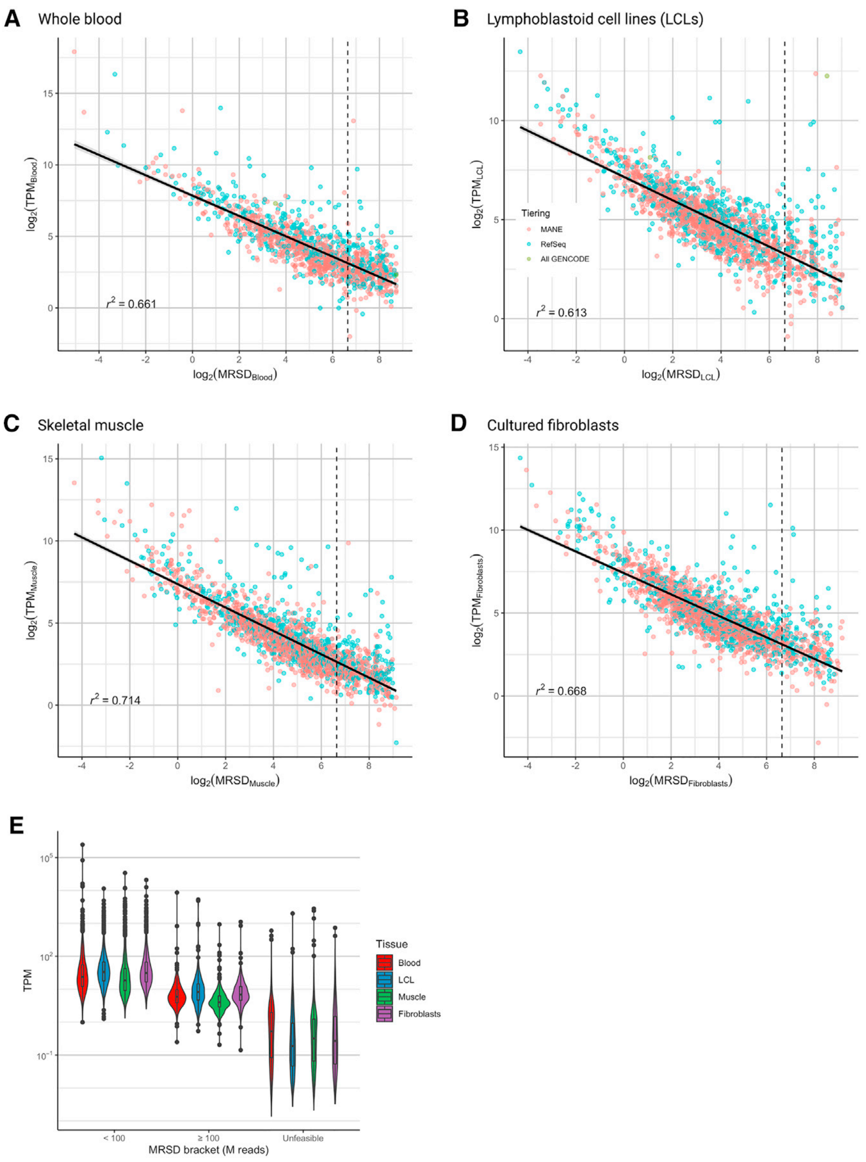

Figure 3. Comparison of MRSD and transcripts per million (TPM) predictions

(A–D) MRSD and TPM predictions for 3322 multiexon genes present in the Genomics PanelApp repository are inversely correlated in (A) whole blood (r^2 = 0.661), (B) LCLs (r^2 = 0.613), (C) skeletal muscle (r^2 = 0.714), and (D) cultured fibroblasts (r^2 = 0.668).

(E) Grouping PanelApp genes by MRSD range shows that there is substantial overlap in the TPMs of genes across different groups, suggesting relative expression level alone is not an adequate proxy for transcript coverage in some cases. Log transformation in (E) excludes 553 entries with TPMs of 0 in the unfeasible group. Default MRSD parameters (8-read coverage of 75% of splice junctions, $MRSD_{0.95}$) used throughout.

and relative expression of genes of interest (Figure S10). Overall, our analyses suggested that filtering for splicing junction supported by ≥2 reads reduces the number of identified events by up to 95% (Figure 4; supplemental results) and that mis-splicing events mostly retain their relative priority ranks at lower expression levels (Figure 5; supplemental results). Based on these investigations and our investigations for 21 known pathogenic splicing events (90% identified with ≥2 reads and NRC > 0.25, Table 1), we selected an 8 read minimum coverage value for downstream analyses.

Implications for investigation of variants in known disease-causing genes

We utilized MRSD scores for 3,322 multi-exon monogenic disease genes using standardized parameters (read coverage = 8; proportion of junctions = 75%; MRSD parameter = 95%). We acknowledge that these parameters may be too lenient for some use cases but expect trends to be similar across other applied MRSD parameter combinations (Figure 6). Using this approach, we observed that 64.2% (2,133/3,322) of PanelApp genes were predicted to be low-MRSD (<100 M reads required) in at least one of the four tissues (Figures 6A and S9). At the individual tissue level, 28.2% (936/3,322) of PanelApp genes in whole blood, 49.4% (1,641/3,322) in LCLs, 43.6% (1,447/3,322) in skeletal muscle, and 53.7% (1,784/3,322) in cultured fibroblasts were predicted to be low-MRSD (Figure 6A). Fibroblasts were observed to have the highest (or joint-highest) proportion of low-MRSD panel genes in 186/295 disease gene panels (63.1%, Figure 6C) compared to 126/295 panels for LCLs (42.7%), 70/295 panels (23.7%) for skeletal muscle, and 21/295 panels (7.1%) for whole blood (Figure S11).

MRSD predictions revealed many use cases for specific tissues: in the familial rhabdomyosarcoma panel, for example, none of the 11 genes were predicted to be low-MRSD in blood, while 10/11 were predicted low-MRSD in LCLs (Figure 6C), of which 9 were actually assigned an MRSD < 50 M reads. Results across all 295 panels are shown in Figures S12 and S13.

Quantifying the resolving power of RNA-seq for variants of uncertain significance

To analyze the possible impact of RNA-seq integration on variant interpretation, we curated variants of uncertain significance (VUSs) from the ClinVar variant database[21] that were predicted by SpliceAI[22] to impact splicing (score ≥ 0.5; see material and methods). Of a total of 352,011

Table 1. Range of metrics observed for pathogenic splicing events

	Tissue		
**Metric**	**Whole blood (n = 3)**	**LCLs (n = 7)**	**Skeletal muscle (n = 11)**
Read count	2–40	4–38	2–462
NRC	0.48–1.25	0.19–1.52	0.34–3.19
NRC fold change	singletons	3.7–8.2 + singletons	19.6–442 + singletons
Number of samples	1	1–48	1–110
Rank	2-5	10–232	1–342
FRASER events identified	3/3	4/7	10/11
FRASER p values	7.97×10^{-11}–0.0022	2.36×10^{-5}–0.13182	4.27×10^{-13}–0.0160
LeafCutterMD events identified	3/3	2/7	7/11
LeafCutterMD p values	6.19×10^{-11}–0.00936	7.66×10^{-6}–0.586	2.2×10^{-15}–1.35×10^{-3}
SPOT events identified	3/3	6/7	7/11
SPOT p values	0.000181–0.0426	1×10^{-6}–0.13582	0.00469–0.0159

ClinVar variants, 185,119 (52.6%) were identified as VUS, and 7,507 (2.1%) were retained after filtering based on SpliceAI score. Cross-referencing the MRSDs of the transcripts harboring SpliceAI-prioritized variants across tissues revealed that, at a specified read coverage of 8 reads, between 25.8% and 67.8% of these variants may lie in genes that are low-MRSD in at least one of the four tissues (Figure 7A), dependent on the stringency of the model (Figure S14). Further, among the 30 genes in which the greatest number of predicted splice-impacting VUSs were identified, 76% (23/30) were predicted to be low-MRSD in at least one tissue (Figure 7B) at a desired read coverage of 8 reads. This is reduced to 73% (22/30) and 60% (18/30) of genes at desired read coverages of 10 and 20 reads, respectively.

Discussion

Implementation of machine learning approaches has improved the ability to prioritize variants that impact splicing and cause rare disease.[23] Despite these advances, corroboration of the effect of such variants remains a major obstacle. This is amplified by unexpected impacts that many variants may have on mRNA splicing.[6]

The MRSD-based approach that we describe here allows informed selection of biosample(s) for bulk RNA-seq, based on the required number of sequencing reads for appropriate surveillance of genes of interest. This enables effective patient-specific identification of genomic variants that are amenable for functional assessment of mis-splicing through RNA-seq. This can improve efficiency and accuracy of genomic diagnostic approaches. Although our model is conservative (Figure 2), we demonstrate through MRSD-guided re-inspection of VUSs in ClinVar that it may be possible to use RNA-seq to clarify the effect of >5,000 variants of uncertain significance (Figure 7A).

Other approaches to select genes amenable to functional analysis through RNA-seq include leveraging relative gene expression metrics[14,24] or tools which assess the similarity

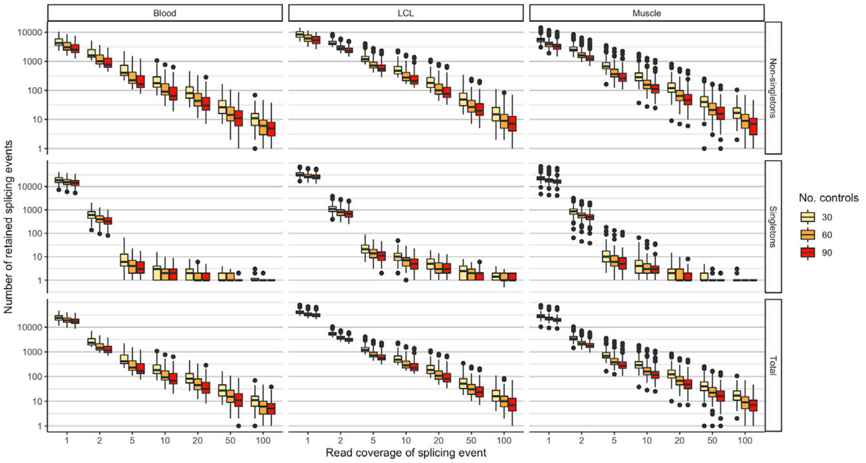

Figure 4. Expanding control datasets and enforcing read count thresholds improves filtering power when analyzing mis-splicing events
There is a small decrease in the number of splicing events identified with increasing control size. Enforcing a read coverage threshold has a more significant effect on event counts, particularly for singleton events, where filtering out events supported by a single read removes up to 95% of singleton events. LCLs appear to exhibit the greatest number of splicing events regardless of read count filter, although this may be due to differences in sequencing depth between tissues. These data are generated from 2,000 bootstraps for control sizes of 30, 60, and 90 individuals. Outliers represent data points lying further than 1.5 times the interquartile range from the 25th and 75th percentile values.

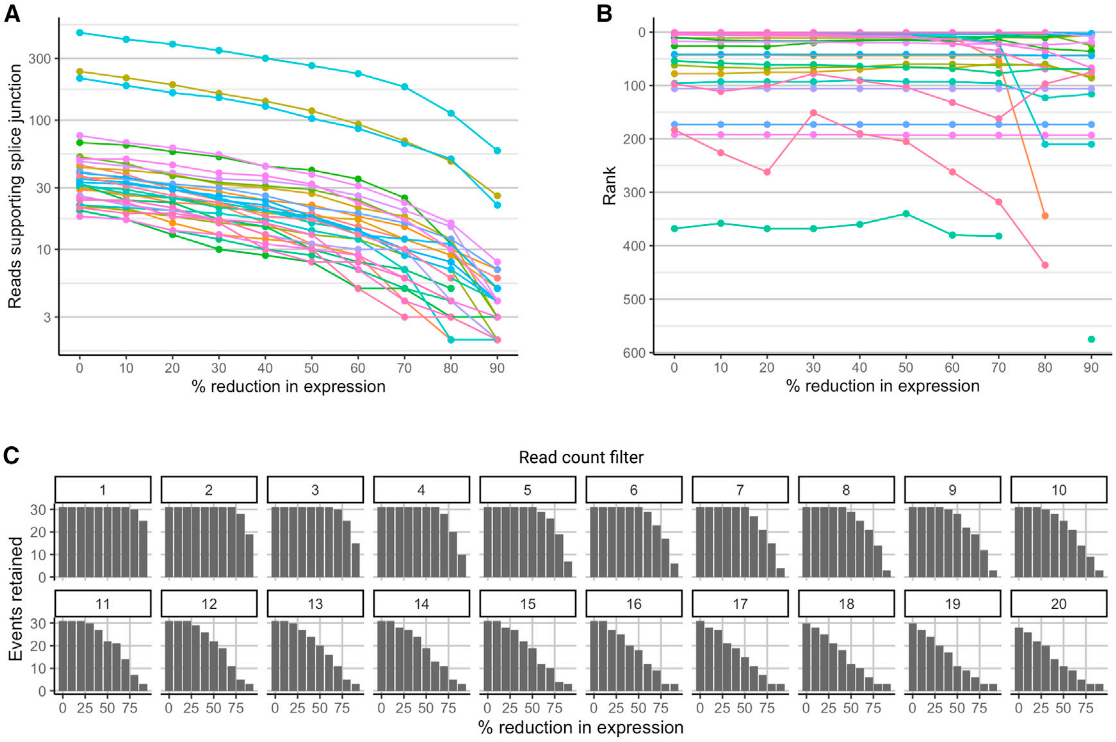

Figure 5. Variability in expression level influences the capacity to identify mis-splicing events

Genes harboring a selection of 31 splicing events that were identified during analysis of 52 muscle-based RNA-seq datasets (and which would be identified as events of interest using a filter of normalized read count [NRC] > 0.19) were artificially downsampled to simulate variation in expression.

(A) Reduction in expression leads to an intuitive and proportional reduction in the number of reads supporting each mis-splicing event.

(B) The rank position of an event—where the event appears in a list of all splicing events in its respective sample, ordered by decreasing NRC fold change relative to controls, and placing singleton events above non-singletons—is generally consistent as expression of the gene decreases. Missing data points at the most reduced expression values are indicative of the splicing event not being identified by the applied bioinformatics pipeline.

(C) Variation in expression impacts our ability to identify events of interest when filters of read count supporting the events are enforced. When the 31 events experience a 50% reduction in expression, for instance, the application of a minimum 15-read filter leads to the exclusion of 41.9% (13/31) of events.

of transcript isoforms between tissues, e.g., MAGIQ-CAT.[7] We show that, while TPM values are well correlated with MRSD scores (Figures 3A–3C), uneven sequencing coverage across the length of the transcript may, in some cases, falsely identify specific genes or splice junctions as being amenable to RNA-seq-based analysis (Figure S8). 3′ sequencing bias, which is a known artifact of poly-A enriched mRNA sequencing,[25–27] and alternative transcript usage across tissues may elevate the risk of inaccurately selecting genes that could be surveyed through RNA-seq when considering TPM alone. Additionally, the normalization against sequencing depth that occurs during the calculation of TPM obscures information about raw read count at the level of individual splicing junctions, which is important when analyzing the utility of RNA-seq for clinical diagnostics. MRSD scoring, conversely, leverages variation in sample read depth to provide quantitative predictions about optimal sequencing depths.

Other bioinformatics tools may complement the utility of MRSD; MAGIQ-CAT[7] assesses the degree to which transcript isoforms in a sampled tissue accurately resemble those in the primary disease-affected tissue. However, MAGIQ-CAT primarily captures the degree of similarity between isoform structure and does not aim to provide a quantitative readout to guide biosample suitability. We envision that the use of both MAGIQ-CAT and MRSD could comprehensively capture information about the utility of RNA-seq, both in terms of similarity of isoform structure relative to the disease-affected tissue and in terms of the capability of observing disruptions to this structure at specific sequencing read depths. Future investigations of the stability of MRSD scores for tissue-specific and tissue-shared transcripts will be of interest.

There are limitations of the current MRSD model, which could be incorporated into future work. First, the MRSD model cannot directly be extended to predict the suitability of datasets to detect allele-specific expression biases and differential gene expression, which are known hallmarks of pathogenic mechanisms in known disease-causing genes.[10,11,14,28] Although further investigations are required to quantify and prove this suitability, it is likely that genes with low MRSD scores (Figure 3D) are

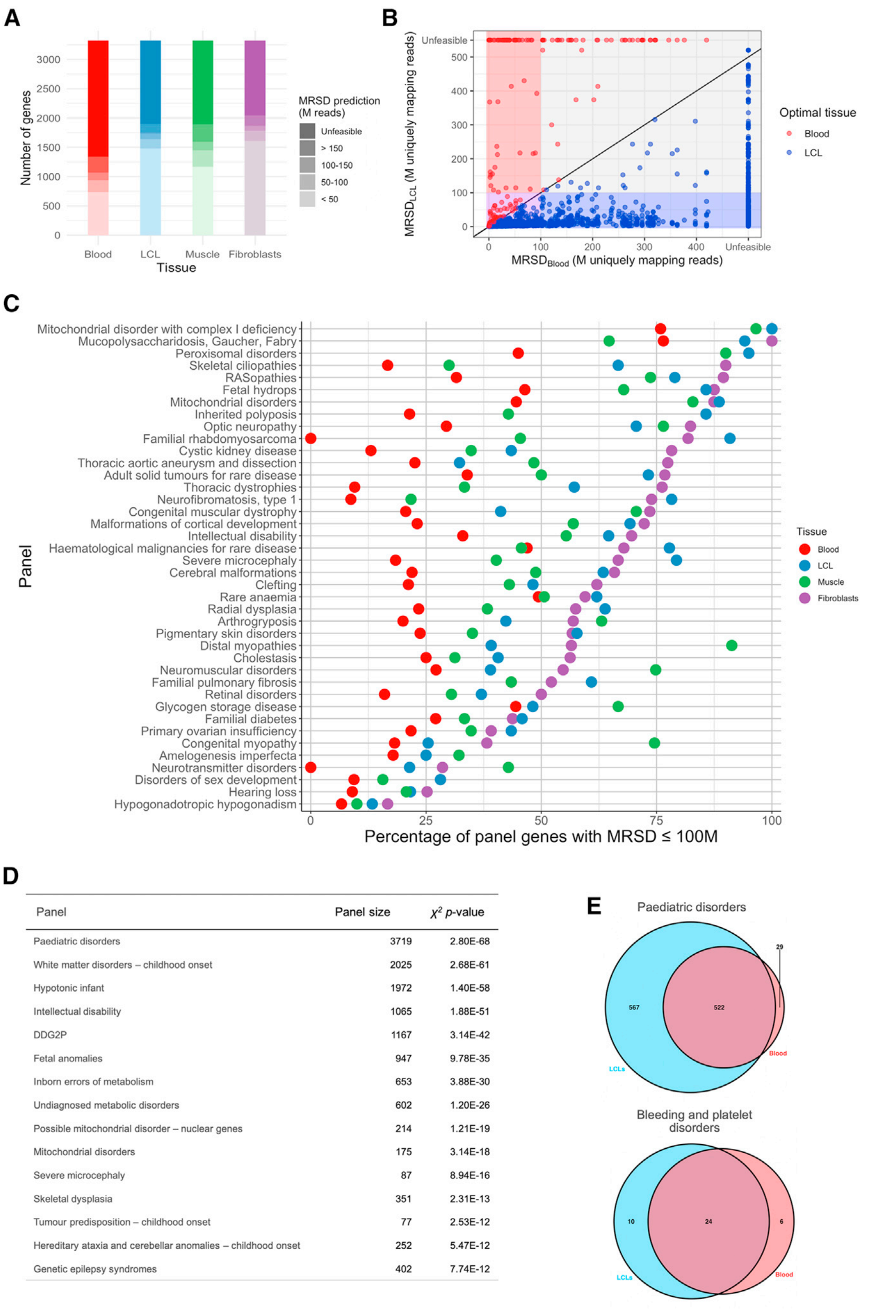

Panel	Panel size	χ^2 p-value
Paediatric disorders	3719	2.80E-68
White matter disorders – childhood onset	2025	2.68E-61
Hypotonic infant	1972	1.40E-58
Intellectual disability	1065	1.88E-51
DDG2P	1167	3.14E-42
Fetal anomalies	947	9.78E-35
Inborn errors of metabolism	653	3.88E-30
Undiagnosed metabolic disorders	602	1.20E-26
Possible mitochondrial disorder – nuclear genes	214	1.21E-19
Mitochondrial disorders	175	3.14E-18
Severe microcephaly	87	8.94E-16
Skeletal dysplasia	351	2.31E-13
Tumour predisposition – childhood onset	77	2.53E-12
Hereditary ataxia and cerebellar anomalies – childhood onset	252	5.47E-12
Genetic epilepsy syndromes	402	7.74E-12

Figure 6. Application of MRSD scores to disease genes listed in the Genomics England PanelApp repository
(A) Comparison of PanelApp panel gene MRSD predictions between tissues shows blood to exhibit markedly poorer coverage of disease genes than other tissues.
(B) When comparing MRSD predictions for genes in blood and LCLs, 1,522 genes are considered “high-MRSD” (i.e., have an MRSD ≥ 100 M reads) in both tissues (gray). Genes which are exclusively low-MRSD (i.e., MRSD < 100 M) in blood are far fewer in number (with 66 genes, red box), while the remainder are low-MRSD in both (775 genes, purple box) or low-MRSD in LCLs only (749 genes, blue box).
(C) Comparison of PanelApp panel gene MRSDs between tissues shows many panel genes have greater coverage in fibroblasts than blood and, to a lesser extent, LCLs and skeletal muscle over a variety of disease subtypes. 40 exemplar gene panels are shown here, see Figures S12 and S13 for all 295 PanelApp gene panels.
(D) Top 10 panels with most significant difference between low- and high-MRSD gene counts between blood and LCLs (chi-square test).
(E) Venn diagrams showing number of low-MRSD genes predicted in blood and LCLs for two exemplar disease gene panels.

expression levels may disrupt our ability to reliably highlight pathogenic splicing events (Figure 5C). As a greater number of paired transcriptome and genomic datasets become available, we expect that MRSD scores can be generated in a dynamic manner to account for the presence of eQTLs, sQTLs, other modifiers of gene expression profiles, and multiple testing issues that may arise from surveying multiple splice junctions and/or VUSs of interest for splicing aberrations through RNA-seq.

also amenable to investigations of differential gene expression and isoform imbalance.

Second, further extensions to the model could incorporate genomic background which influences gene expression profiles. For example, MRSD predictions may not accurately reflect the degree of sequencing coverage for certain transcripts in patients with disorders associated with widespread changes to the transcriptome, e.g., interferonopathies,[29–31] chromatin structure disorders,[32,33] and disruption of the spliceosome.[34–36] Moreover, the current MRSD model does not explicitly account for the presence of expression quantitative trait loci (eQTLs) or splicing quantitative trait loci (sQTLs) which are known to influence gene expression profiles.[37–39] We have demonstrated that modulation in

Third, our approach is built for a specific cohort of RNA-seq-based analyses; specifically, the analysis of a selection of tissues by bulk short-read poly-A enrichment RNA-seq processed using a specific bioinformatics analysis pipeline.[13] This specific RNA-seq approach currently remains widespread;[13–15] the behavior of MRSD scores for other experimental and/or bioinformatics approaches will be an interesting avenue for further research. However, our data suggest that the MRSD model may be readily applicable to RNA-seq generated using alternative methodologies, such as increased read length, with only minor variations in model performance (Figure S6). As other technologies, such as long-read,[40–42] single-cell,[43,44] and spatially resolved RNA-seq,[45–48] become more prevalent in a clinical setting,

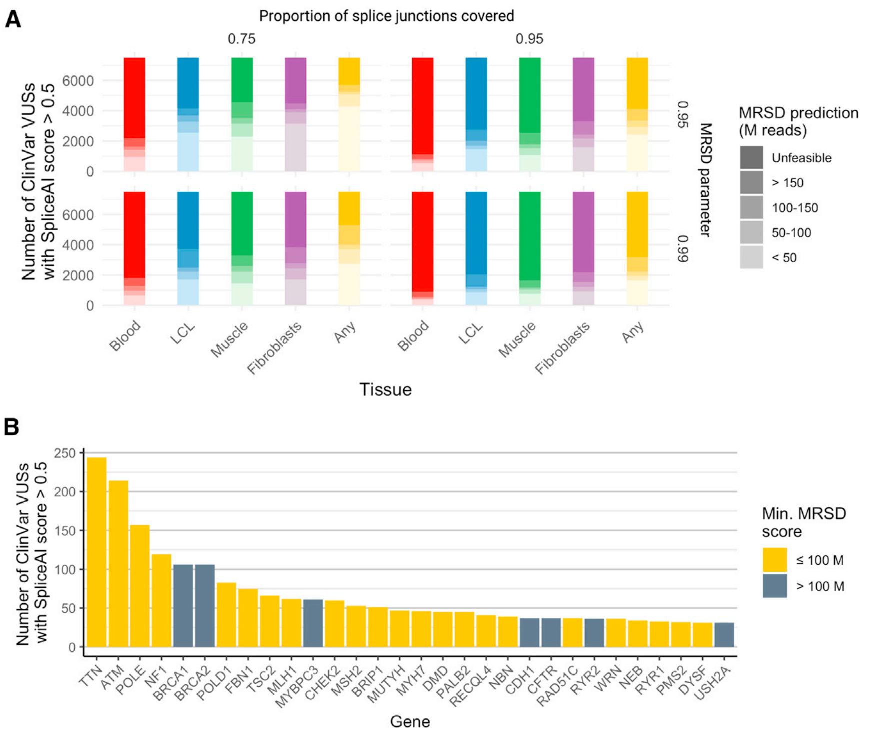

Figure 7. Quantifying the power for RNA-seq to resolve variants of uncertain significance (VUSs)

MRSD scores were derived for genes harboring VUSs present in ClinVar if the variants were predicted by SpliceAI to impact splicing (score $\geq$ 0.5; Jaganathan et al.[22]).

(A) Between 25.8% (1,940/7,507) and 67.8% (5,086/7,507) of variants predicted to impact splicing are expected to be adequately covered by 100 M uniquely mapping reads or fewer in at least one of the four tissues (whole blood, LCLs, skeletal muscle, and fibroblasts), dependent on model stringency. Variants were most likely to be found to be in low-MRSD genes (MRSD $\leq$ 100 M) in fibroblasts, irrespective of model parameters.

(B) Among the 30 genes with the greatest number of predicted splice-impacting VUSs, 23 were predicted to be adequately covered (using default parameters) with 100 M uniquely mapping reads or fewer in at least one of the four tissues. An 8-read junction support parameter was used throughout.

appropriate control datasets must be generated to develop corresponding MRSD models. Similarly, recent research has shown noticeable improvements to diagnostic yield for neuromuscular disorders by conducting RNA-seq on *in vitro* myofibrils generated by a fibroblast-to-myofibril transdifferentiation protocol.[49] Such patient-derived cell line approaches represent a promising avenue to scrutinize transcripts not otherwise observable in proxy tissues.[35,50] As these protocols gain wider use, generation of control RNA-seq data from healthy individuals using these approaches will be vital both to allow the generation of MRSD scores and to accurately assess pathogenicity of any identified mis-splicing events.

In summary, the MRSD model presented here offers a gene-specific readout to predict the most suitable biosample for interrogation of splicing disruption at the transcript level. This may uncover previously unintuitive choices of biosample, as discussed above in the case of familial rhabdomyosarcoma (Figure 6C). We expect that the use of MRSD will allow effective and appropriate integration of RNA-seq into diagnostic genomic services and ultimately improve variant interpretation and diagnostic yield.

Data and code availability

The control datasets used to generate the MRSD model are available through the dbGaP repository (see web resources) under controlled access through the GTEx v8 data release (data used in this study was accessed through accession ID: phs000424.v8.p2). Muscle-derived RNA-seq datasets to test the MRSD model are available through dbGaP (accessed in this study through accession ID: phs000655.v3.p1.c1). Source code for MRSD calculation and pre-computed MRSD scores for all GENCODE v.19 genes across the four investigated tissues are available (see web resources). MRSD resources are made freely available without access control.

Supplemental information

Supplemental information can be found online at https://doi.org/10.1016/j.ajhg.2021.12.014.

Consortia

The members of the kConFab Investigators are David Amor, Lesley Andrews, Yoland Antill, Rosemary Balleine, Jonathan Beesley, Ian Bennett, Michael Bogwitz, Leon Botes, Meagan Brennan, Melissa Brown, Michael Buckley, Jo Burke, Phyllis Butow, Liz Caldon, Ian Campbell, Deepa Chauhan, Manisha Chauhan, Georgia Chenevix-Trench, Alice Christian, Paul Cohen, Alison Colley, Ashley Crook, James Cui, Margaret Cummings, Sarah-Jane Dawson, Anna DeFazio, Martin Delatycki, Rebecca Dickson, Joanne Dixon, Ted Edkins, Stacey Edwards, Gelareh Farshid, Andrew Fellows, Georgina Fenton, Michael Field, James Flanagan, Peter Fong, Laura Forrest, Stephen Fox, Juliet French, Michael Friedlander, Clara Gaff, Mike Gattas, Peter George, Sian Greening, Marion Harris, Stewart Hart, Nick Hayward, John Hopper, Cass Hoskins, Clare Hunt, Paul James, Mark Jenkins, Alexa Kidd, Judy Kirk, Jessica Koehler, James Kollias, Sunil Lakhani, Mitchell Lawrence, Geoff Lindeman, Lara Lipton, Liz Lobb, Graham Mann, Deborah Marsh, Sue Anne McLachlan, Bettina Meiser, Roger Milne, Sophie Nightingale, Shona O'Connell, Sarah O'Sullivan, David Gallego Ortega, Nick Pachter, Briony Patterson, Amy Pearn, Kelly Phillips, Ellen Pieper, Edwina Rickard, Bridget Robinson, Mona Saleh, Elizabeth Salisbury, Christobel Saunders, Jodi Saunus, Rodney Scott, Clare Scott, Adrienne Sexton, Andrew Shelling, Peter Simpson, Melissa Southey, Amanda Spurdle, Jessica Taylor, Renea Taylor, Heather Thorne, Alison Trainer, Kathy Tucker, Jane Visvader, Logan Walker, Rachael Williams, Ingrid Winship, and Mary Ann Young.

Acknowledgments

C.F.R. is funded by the Medical Research Council (MRC; 1926882) as part of a CASE studentship with QIAGEN. The Baralle lab is supported by an NIHR Research Professorship to D.B. (RP-2016-07-011). W.G.N. is supported by the NIHR Manchester Biomedical Research Centre (IS-BRC-1215-20007). We acknowledge funding from the Wellcome Trust Transforming Genomic Medicine Initiative (200990/Z/16/Z) and the Medical Research Foundation. J.M.E. is funded by a postdoctoral research fellowship from the Health Education England Genomics Education Programme (HEE GEP). The views expressed in this publication are those of the authors and not necessarily those of the HEE GEP.

We wish to thank Heather Thorne, Eveline Niedermayr, all the kConFab research nurses and staff, the heads and staff of the Family Cancer Clinics, and the Clinical Follow Up Study (which has received funding from the NHMRC, the National Breast Cancer Foundation, Cancer Australia, and the National Institutes of Health, USA) for their contributions to this resource, and the many families who contribute to kConFab. kConFab is supported by a grant from the National Breast Cancer Foundation, and previously by the National Health and Medical Research Council (NHMRC), the Queensland Cancer Fund, the Cancer Councils of New South Wales, Victoria, Tasmania, and South Australia, and the Cancer Foundation of Western Australia. We also wish to thank members of the Wessex Investigational Sciences Hub (WISH) Laboratory, Southampton, UK, for their help in facilitating RNA-seq of kConFab LCL samples (particularly Christopher Mattocks, Daniel Ward, and Jade Forster) as well as the work of the University of Manchester Genomics Core Technology and Bioinformatics Facilities for their assistance in sample processing.

Declaration of interests

The authors declare no competing interests.

Received: May 10, 2021
Accepted: December 12, 2021
Published: January 21, 2022

Web resources

dbGaP, https://www.ncbi.nlm.nih.gov/gap/
MRSD web portal, https://mcgm-mrsd.github.io/

References

1. Anna, A., and Monika, G. (2018). Splicing mutations in human genetic disorders: examples, detection, and confirmation. J. Appl. Genet. *59*, 253–268. https://doi.org/10.1007/s13353-018-0444-7.
2. Scotti, M.M., and Swanson, M.S. (2016). RNA mis-splicing in disease. Nat. Rev. Genet. *17*, 19–32. https://doi.org/10.1038/nrg.2015.3.
3. Wai, H.A., Lord, J., Lyon, M., Gunning, A., Kelly, H., Cibin, P., Seaby, E.G., Spiers-Fitzgerald, K., Lye, J., Ellard, S., et al.; Splicing and disease working group (2020). Blood RNA analysis can increase clinical diagnostic rate and resolve variants of uncertain significance. Genet. Med. *22*, 1005–1014. https://doi.org/10.1038/s41436-020-0766-9.
4. Sangermano, R., Garanto, A., Khan, M., Runhart, E.H., Bauwens, M., Bax, N.M., van den Born, L.I., Khan, M.I., Cornelis, S.S., Verheij, J.B.G.M., et al. (2019). Deep-intronic ABCA4 variants explain missing heritability in Stargardt disease and allow correction of splice defects by antisense oligonucleotides. Genet. Med. *21*, 1751–1760. https://doi.org/10.1038/s41436-018-0414-9.
5. Khan, M., Cornelis, S.S., Pozo-Valero, M.D., Whelan, L., Runhart, E.H., Mishra, K., Bults, F., AlSwaiti, Y., AlTalbishi, A., De Baere, E., et al. (2020). Resolving the dark matter of ABCA4 for 1054 Stargardt disease probands through integrated genomics and transcriptomics. Genet. Med. *22*, 1235–1246. https://doi.org/10.1038/s41436-020-0787-4.
6. Rowlands, C., Thomas, H.B., Lord, J., Wai, H.A., Arno, G., Beaman, G., Sergouniotis, P., Gomes-Silva, B., Campbell, C., Gossan, N., et al.; Genomics England Research Consortium (2021). Comparison of in silico strategies to prioritize rare genomic variants impacting RNA splicing for the diagnosis of genomic disorders. Sci. Rep. *11*, 20607, 10.103/s41598-021-99747-2.
7. Aicher, J.K., Jewell, P., Vaquero-Garcia, J., Barash, Y., and Bhoj, E.J. (2020). Mapping RNA splicing variations in clinically accessible and nonaccessible tissues to facilitate Mendelian disease diagnosis using RNA-seq. Genet. Med. *22*, 1181–1190. https://doi.org/10.1038/s41436-020-0780-y.
8. Marston, S., Copeland, O., Jacques, A., Livesey, K., Tsang, V., McKenna, W.J., Jalilzadeh, S., Carballo, S., Redwood, C., and Watkins, H. (2009). Evidence from human myectomy samples that MYBPC3 mutations cause hypertrophic cardiomyopathy through haploinsufficiency. Circ. Res. *105*, 219–222. https://doi.org/10.1161/CIRCRESAHA.109.202440.
9. Mertes, C., Scheller, I.F., Yépez, V.A., Çelik, M.H., Liang, Y., Kremer, L.S., Gusic, M., Prokisch, H., and Gagneur, J. (2021). Detection of aberrant splicing events in RNA-seq data using FRASER. Nat. Commun. *12*, 529. https://doi.org/10.1038/s41467-020-20573-7.
10. Kremer, L.S., Bader, D.M., Mertes, C., Kopajtich, R., Pichler, G., Iuso, A., Haack, T.B., Graf, E., Schwarzmayr, T., Terrile, C., et al. (2017). Genetic diagnosis of Mendelian disorders via RNA sequencing. Nat. Commun. *8*, 15824. https://doi.org/10.1038/ncomms15824.
11. Byron, S.A., Van Keuren-Jensen, K.R., Engelthaler, D.M., Carpten, J.D., and Craig, D.W. (2016). Translating RNA sequencing into clinical diagnostics: opportunities and challenges. Nat. Rev. Genet. *17*, 257–271. https://doi.org/10.1038/nrg.2016.10.
12. Marco-Puche, G., Lois, S., Benítez, J., and Trivino, J.C. (2019). RNA-Seq Perspectives to Improve Clinical Diagnosis. Front. Genet. *10*, 1152. https://doi.org/10.3389/fgene.2019.01152.
13. Cummings, B.B., Marshall, J.L., Tukiainen, T., Lek, M., Donkervoort, S., Foley, A.R., Bolduc, V., Waddell, L.B., Sandaradura, S.A., O'Grady, G.L., et al.; Genotype-Tissue Expression Consortium (2017). Improving genetic diagnosis in Mendelian disease with transcriptome sequencing. Sci. Transl. Med. *9*, eaal5209. https://doi.org/10.1126/scitranslmed.aal5209.
14. Frésard, L., Smail, C., Ferraro, N.M., Teran, N.A., Li, X., Smith, K.S., Bonner, D., Kernohan, K.D., Marwaha, S., Zappala, Z., et al.; Undiagnosed Diseases Network; and Care4Rare Canada Consortium (2019). Identification of rare-disease genes using blood transcriptome sequencing and large control cohorts. Nat. Med. *25*, 911–919. https://doi.org/10.1038/s41591-019-0457-8.
15. Lee, H., Huang, A.Y., Wang, L.K., Yoon, A.J., Renteria, G., Eskin, A., Signer, R.H., Dorrani, N., Nieves-Rodriguez, S., Wan,

J., et al.; Undiagnosed Diseases Network (2020). Diagnostic utility of transcriptome sequencing for rare Mendelian diseases. Genet. Med. *22*, 490–499. https://doi.org/10.1038/s41436-019-0672-1.

16. Johnston, J.J., Williamson, K.A., Chou, C.M., Sapp, J.C., Ansari, M., Chapman, H.M., Cooper, D.N., Dabir, T., Dudley, J.N., Holt, R.J., et al. (2019). *NAA10* polyadenylation signal variants cause syndromic microphthalmia. J. Med. Genet. *56*, 444–452. https://doi.org/10.1136/jmedgenet-2018-105836.
17. Ferraro, N.M., Strober, B.J., Einson, J., Abell, N.S., Aguet, F., Barbeira, A.N., Brandt, M., Bucan, M., Castel, S.E., Davis, J.R., et al.; TOPMed Lipids Working Group; and GTEx Consortium (2020). Transcriptomic signatures across human tissues identify functional rare genetic variation. Science *369*, eaaz5900. https://doi.org/10.1126/science.aaz5900.
18. Jenkinson, G., Li, Y.I., Basu, S., Cousin, M.A., Oliver, G.R., and Klee, E.W. (2020). LeafCutterMD: an algorithm for outlier splicing detection in rare diseases. Bioinformatics *36*, 4609–4615. https://doi.org/10.1093/bioinformatics/btaa259.
19. Osborne, R.H., Hopper, J.L., Kirk, J.A., Chenevix-Trench, G., Thorne, H.J., Sambrook, J.F.; and Kathleen Cuningham Foundation Consortium for Research into Familial Breast Cancer (2000). kConFab: a research resource of Australasian breast cancer families. Med. J. Aust. *172*, 463–464.
20. Dobin, A., Davis, C.A., Schlesinger, F., Drenkow, J., Zaleski, C., Jha, S., Batut, P., Chaisson, M., and Gingeras, T.R. (2013). STAR: ultrafast universal RNA-seq aligner. Bioinformatics *29*, 15–21. https://doi.org/10.1093/bioinformatics/bts635.
21. Landrum, M.J., Lee, J.M., Benson, M., Brown, G.R., Chao, C., Chitipiralla, S., Gu, B., Hart, J., Hoffman, D., Jang, W., et al. (2018). ClinVar: improving access to variant interpretations and supporting evidence. Nucleic Acids Res. *46* (D1), D1062–D1067. https://doi.org/10.1093/nar/gkx1153.
22. Jaganathan, K., Kyriazopoulou Panagiotopoulou, S., McRae, J.F., Darbandi, S.F., Knowles, D., Li, Y.I., Kosmicki, J.A., Arbelaez, J., Cui, W., Schwartz, G.B., et al. (2019). Predicting Splicing from Primary Sequence with Deep Learning. Cell *176*, 535–548.e24. https://doi.org/10.1016/j.cell.2018.12.015.
23. Rowlands, C.F., Baralle, D., and Ellingford, J.M. (2019). Machine Learning Approaches for the Prioritization of Genomic Variants Impacting Pre-mRNA Splicing. Cells *8*, E1513. https://doi.org/10.3390/cells8121513.
24. Murdock, D.R., Dai, H., Burrage, L.C., Rosenfeld, J.A., Ketkar, S., Müller, M.F., Yépez, V.A., Gagneur, J., Liu, P., Chen, S., et al.; Undiagnosed Diseases Network (2021). Transcriptome-directed analysis for Mendelian disease diagnosis overcomes limitations of conventional genomic testing. J. Clin. Invest. *131*, 141500. https://doi.org/10.1172/JCI141500.
25. Finotello, F., Lavezzo, E., Bianco, L., Barzon, L., Mazzon, P., Fontana, P., Toppo, S., and Di Camillo, B. (2014). Reducing bias in RNA sequencing data: a novel approach to compute counts. BMC Bioinformatics *15* (*Suppl 1*), S7. https://doi.org/10.1186/1471-2105-15-S1-S7.
26. Nagalakshmi, U., Wang, Z., Waern, K., Shou, C., Raha, D., Gerstein, M., and Snyder, M. (2008). The transcriptional landscape of the yeast genome defined by RNA sequencing. Science *320*, 1344–1349. https://doi.org/10.1126/science.1158441.
27. Wang, Z., Gerstein, M., and Snyder, M. (2009). RNA-Seq: a revolutionary tool for transcriptomics. Nat. Rev. Genet. *10*, 57–63. https://doi.org/10.1038/nrg2484.
28. Kukurba, K.R., Zhang, R., Li, X., Smith, K.S., Knowles, D.A., How Tan, M., Piskol, R., Lek, M., Snyder, M., Macarthur, D.G., et al. (2014). Allelic expression of deleterious protein-coding variants across human tissues. PLoS Genet. *10*, e1004304. https://doi.org/10.1371/journal.pgen.1004304.
29. Rodero, M.P., and Crow, Y.J. (2016). Type I interferon-mediated monogenic autoinflammation: The type I interferonopathies, a conceptual overview. J. Exp. Med. *213*, 2527–2538. https://doi.org/10.1084/jem.20161596.
30. Volpi, S., Picco, P., Caorsi, R., Candotti, F., and Gattorno, M. (2016). Type I interferonopathies in pediatric rheumatology. Pediatr. Rheumatol. Online J. *14*, 35. https://doi.org/10.1186/s12969-016-0094-4.
31. Schneider, W.M., Chevillotte, M.D., and Rice, C.M. (2014). Interferon-stimulated genes: a complex web of host defenses. Annu. Rev. Immunol. *32*, 513–545. https://doi.org/10.1146/annurev-immunol-032713-120231.
32. Bélanger, C., Bérubé-Simard, F.A., Leduc, E., Bernas, G., Campeau, P.M., Lalani, S.R., Martin, D.M., Bielas, S., Moccia, A., Srivastava, A., et al. (2018). Dysregulation of cotranscriptional alternative splicing underlies CHARGE syndrome. Proc. Natl. Acad. Sci. USA *115*, E620–E629. https://doi.org/10.1073/pnas.1715378115.
33. Liu, J., Zhang, Z., Bando, M., Itoh, T., Deardorff, M.A., Clark, D., Kaur, M., Tandy, S., Kondoh, T., Rappaport, E., et al. (2009). Transcriptional dysregulation in NIPBL and cohesin mutant human cells. PLoS Biol. *7*, e1000119. https://doi.org/10.1371/journal.pbio.1000119.
34. Wood, K.A., Rowlands, C.F., Qureshi, W.M.S., Thomas, H.B., Buczek, W.A., Briggs, T.A., Hubbard, S.J., Hentges, K.E., Newman, W.G., and O'Keefe, R.T. (2019). Disease modeling of core pre-mRNA splicing factor haploinsufficiency. Hum. Mol. Genet. *28*, 3704–3723. https://doi.org/10.1093/hmg/ddz169.
35. Wood, K.A., Rowlands, C.F., Thomas, H.B., Woods, S., O'Flaherty, J., Douzgou, S., Kimber, S.J., Newman, W.G., and O'Keefe, R.T. (2020). Modelling the developmental spliceosomal craniofacial disorder Burn-McKeown syndrome using induced pluripotent stem cells. PLoS ONE *15*, e0233582. https://doi.org/10.1371/journal.pone.0233582.
36. Buskin, A., Zhu, L., Chichagova, V., Basu, B., Mozaffari-Jovin, S., Dolan, D., Droop, A., Collin, J., Bronstein, R., Mehrotra, S., et al. (2018). Disrupted alternative splicing for genes implicated in splicing and ciliogenesis causes PRPF31 retinitis pigmentosa. Nat. Commun. *9*, 4234. https://doi.org/10.1038/s41467-018-06448-y.
37. Richards, A.L., Jones, L., Moskvina, V., Kirov, G., Gejman, P.V., Levinson, D.F., Sanders, A.R., Purcell, S., Visscher, P.M., Craddock, N., et al.; Molecular Genetics of Schizophrenia Collaboration (MGS); and International Schizophrenia Consortium (ISC) (2012). Schizophrenia susceptibility alleles are enriched for alleles that affect gene expression in adult human brain. Mol. Psychiatry *17*, 193–201. https://doi.org/10.1038/mp.2011.11.
38. Takata, A., Matsumoto, N., and Kato, T. (2017). Genome-wide identification of splicing QTLs in the human brain and their enrichment among schizophrenia-associated loci. Nat. Commun. *8*, 14519. https://doi.org/10.1038/ncomms14519.
39. Westra, H.J., and Franke, L. (2014). From genome to function by studying eQTLs. Biochim. Biophys. Acta *1842*, 1896–1902. https://doi.org/10.1016/j.bbadis.2014.04.024.

40. Mantere, T., Kersten, S., and Hoischen, A. (2019). Long-Read Sequencing Emerging in Medical Genetics. Front. Genet. *10*, 426. https://doi.org/10.3389/fgene.2019.00426.
41. Merker, J.D., Wenger, A.M., Sneddon, T., Grove, M., Zappala, Z., Fresard, L., Waggott, D., Utiramerur, S., Hou, Y., Smith, K.S., et al. (2018). Long-read genome sequencing identifies causal structural variation in a Mendelian disease. Genet. Med. *20*, 159–163. https://doi.org/10.1038/gim.2017.86.
42. Pauper, M., Kucuk, E., Wenger, A.M., Chakraborty, S., Baybayan, P., Kwint, M., van der Sanden, B., Nelen, M.R., Derks, R., Brunner, H.G., et al. (2021). Long-read trio sequencing of individuals with unsolved intellectual disability. Eur. J. Hum. Genet. *29*, 637–648.. https://doi.org/10.1038/s41431-020-00770-0.
43. Del-Aguila, J.L., Li, Z., Dube, U., Mihindukulasuriya, K.A., Budde, J.P., Fernandez, M.V., Ibanez, L., Bradley, J., Wang, F., Bergmann, K., et al. (2019). A single-nuclei RNA sequencing study of Mendelian and sporadic AD in the human brain. Alzheimers Res. Ther. *11*, 71. https://doi.org/10.1186/s13195-019-0524-x.
44. Nomura, S. (2021). Single-cell genomics to understand disease pathogenesis. J. Hum. Genet. *66*, 75–84. https://doi.org/10.1038/s10038-020-00844-3.
45. Crosetto, N., Bienko, M., and van Oudenaarden, A. (2015). Spatially resolved transcriptomics and beyond. Nat. Rev. Genet. *16*, 57–66. https://doi.org/10.1038/nrg3832.
46. Larsson, L., Frisén, J., and Lundeberg, J. (2021). Spatially resolved transcriptomics adds a new dimension to genomics. Nat. Methods *18*, 15–18. https://doi.org/10.1038/s41592-020-01038-7.
47. Marx, V. (2021). Method of the Year: spatially resolved transcriptomics. Nat. Methods *18*, 9–14. https://doi.org/10.1038/s41592-020-01033-y.
48. Navarro, J.F., Croteau, D.L., Jurek, A., Andrusivova, Z., Yang, B., Wang, Y., Ogedegbe, B., Riaz, T., Støen, M., Desler, C., et al. (2020). Spatial Transcriptomics Reveals Genes Associated with Dysregulated Mitochondrial Functions and Stress Signaling in Alzheimer Disease. iScience *23*, 101556. https://doi.org/10.1016/j.isci.2020.101556.
49. Gonorazky, H.D., Naumenko, S., Ramani, A.K., Nelakuditi, V., Mashouri, P., Wang, P., Kao, D., Ohri, K., Viththiyapaskaran, S., Tarnopolsky, M.A., et al. (2019). Expanding the Boundaries of RNA Sequencing as a Diagnostic Tool for Rare Mendelian Disease. Am. J. Hum. Genet. *104*, 1007. https://doi.org/10.1016/j.ajhg.2019.04.004.
50. Lin, M., Pedrosa, E., Shah, A., Hrabovsky, A., Maqbool, S., Zheng, D., and Lachman, H.M. (2011). RNA-Seq of human neurons derived from iPS cells reveals candidate long non-coding RNAs involved in neurogenesis and neuropsychiatric disorders. PLoS ONE *6*, e23356. https://doi.org/10.1371/journal.pone.0023356.

The American Journal of Human Genetics, Volume *109*

Supplemental information

MRSD: A quantitative approach for assessing suitability of RNA-seq in the investigation of mis-splicing in Mendelian disease

Charlie F. Rowlands, Algy Taylor, Gillian Rice, Nicola Whiffin, Hildegard Nikki Hall, William G. Newman, Graeme C.M. Black, kConFab Investigators, Raymond T. O'Keefe, Simon Hubbard, Andrew G.L. Douglas, Diana Baralle, Tracy A. Briggs, and Jamie M. Ellingford

List of Contents

Figure S1
Categories of potentially pathogenic splicing events and their representation in analytical pipeline output

Figure S2
Workflow for MRSD score generation

Figure S3
Sequencing depths of RNA-seq samples used for evaluation of MRSD model accuracy

Figure S4
MRSD scores vary among the different transcripts of individual genes

Figure S5
Transcripts deemed as unfeasible through hierarchical selection are themselves likely to be unfeasible

Figure S6
Effect of varying sequencing read length on MRSD model performance

Figure S7
MRSD scores are generally lower when derived from RNA-seq runs of longer read length

Figure S8
Evidence for 3' sequencing bias confounding the use of TPM as a guiding RNA-seq metric

Figure S9
Exemplar events identified during pathogenic splice event analysis

Figure S10
Relative gene expression level does not reflect the raw read coverage of transcript splice junctions

Figure S11
Pairwise comparisons, by tissue, of MRSD scores for PanelApp disease gene

Figure S12
Proportion of low-MRSD genes per tissue for all PanelApp panels, ordered by panel size

- **a) Low-MRSD gene proportions for large panels (> 50 genes)**
- **b) Low-MRSD gene proportions for medium panels (21-50 genes)**
- **c) Low-MRSD gene proportions for small panels (11-20 genes)**
- **d) Low-MRSD gene proportions for very small panels (≤ 10 genes)**

Figure S13
Proportion of low-MRSD genes per tissue for all PanelApp panels, ordered alphabetically by panel name

- **a) Low-MRSD gene proportions for panels named A-E**
- **b) Low-MRSD gene proportions for panels named F-L**
- **c) Low-MRSD gene proportions for panels named M-R**
- **d) Low-MRSD gene proportions for panels named S-X**

Figure S14
Increasing specified read coverage reduces the number of ClinVar variants that can be analyzed

Figure S15
Increasing specified read count removes highly VUS-prone genes from the scope of analysis

Table S1
Summary of pathogenic splicing variants analyzed during this study

Table S2
Summary of datasets used in this study

Methods S1
Illustration of MRSD calculation methodology

Methods S2
Tiering methodology for selection of transcripts for MRSD generation

Methods S3
Tissue-specific criteria for filtering of high-quality GTEx control RNA-seq datasets

Methods S4
Sample IDs of GTEx samples used to generate control datasets

- **Skeletal muscle**
- **Whole blood**
- **EBV-transformed lymphocytes (LCLs)**
- **Cultured fibroblasts**

Supplementary Results

References

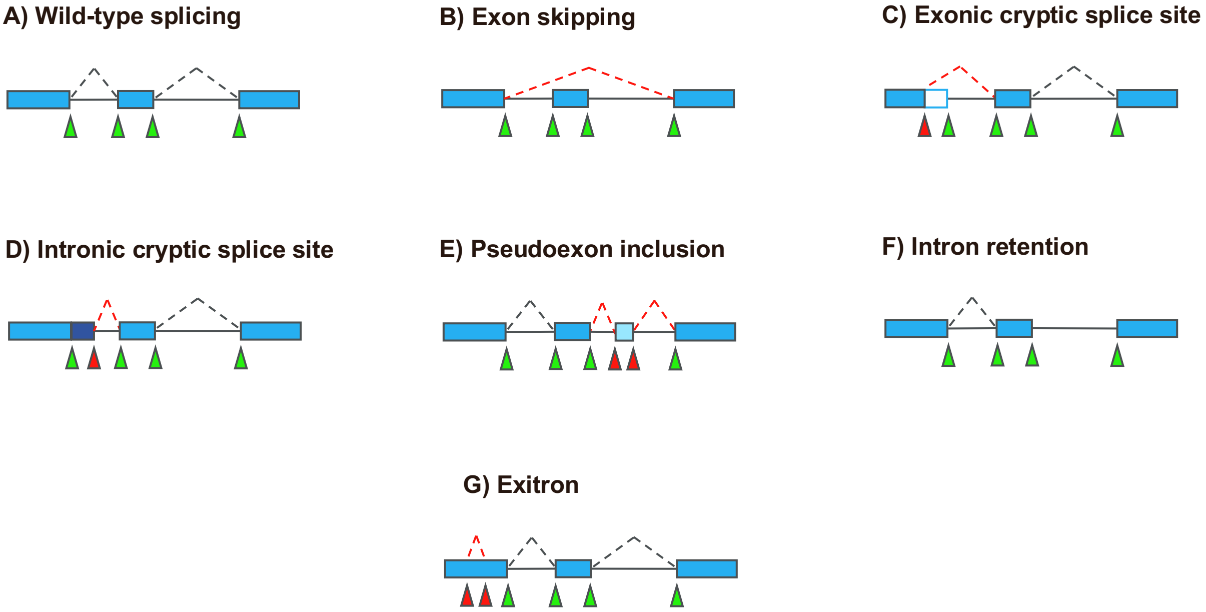

Figure S1. *Categories of potentially pathogenic splicing events and their representation in analytical pipeline output.* Disruption of (**A**) wild-type splicing may lead to (**B**) skipping of one or more exons, the creation of novel splice sites in (**C**) exonic or (**D**) intronic regions that may outcompete the canonical sites, or result in (**E**) the generation of an intronic pseudoexon. (**F**) Splicing may be abrogated completely, leading to total retention of the intron. (**G**) Within longer exons, creation of a novel splice site may lead to a so-called “exitron”, whereby a central portion of the exon is absent from the final transcript. Green triangles indicate canonical splice sites; red triangles indicate non-canonical sites.

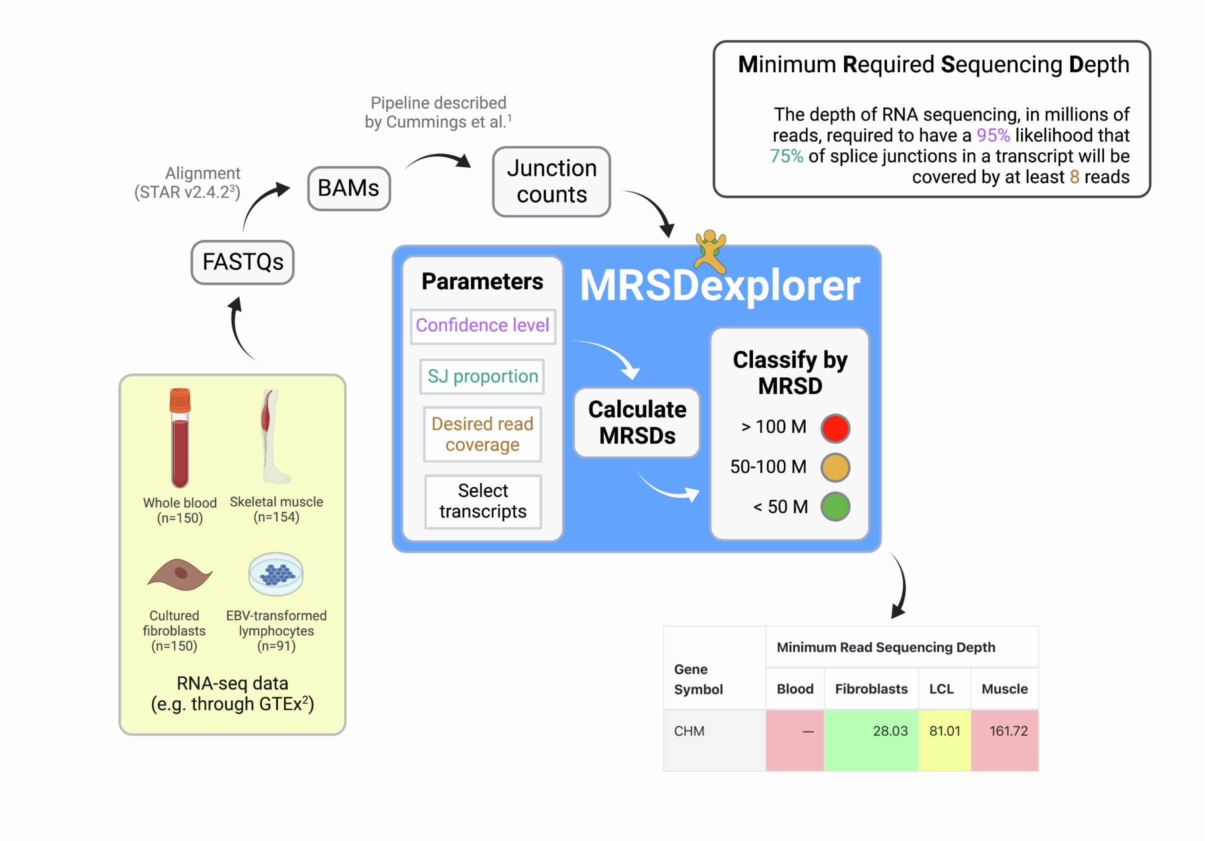

Figure S2. *Workflow for MRSD score generation.* Users can create their own MRSD scores using the code provided online at https://github.com/mcgm-mrsd/mrsd-explorer. Starting with a set of RNA-seq samples, reads are aligned and the split reads counted using an established pipeline. Then, using our bespoke Python scripts, users can generate their own predictive scores (using parameters of their choice) and classify transcripts according to the level of sequencing required to obtain the specified coverage. Alternatively, users are free to investigate pre-computed scores for all GENCODE v19 genes across four tissues (whole blood, skeletal muscle, cultured fibroblasts and lymphoblastoid cell lines, or LCLs) at our web portal: http://mcgm-mrsd.github.io/

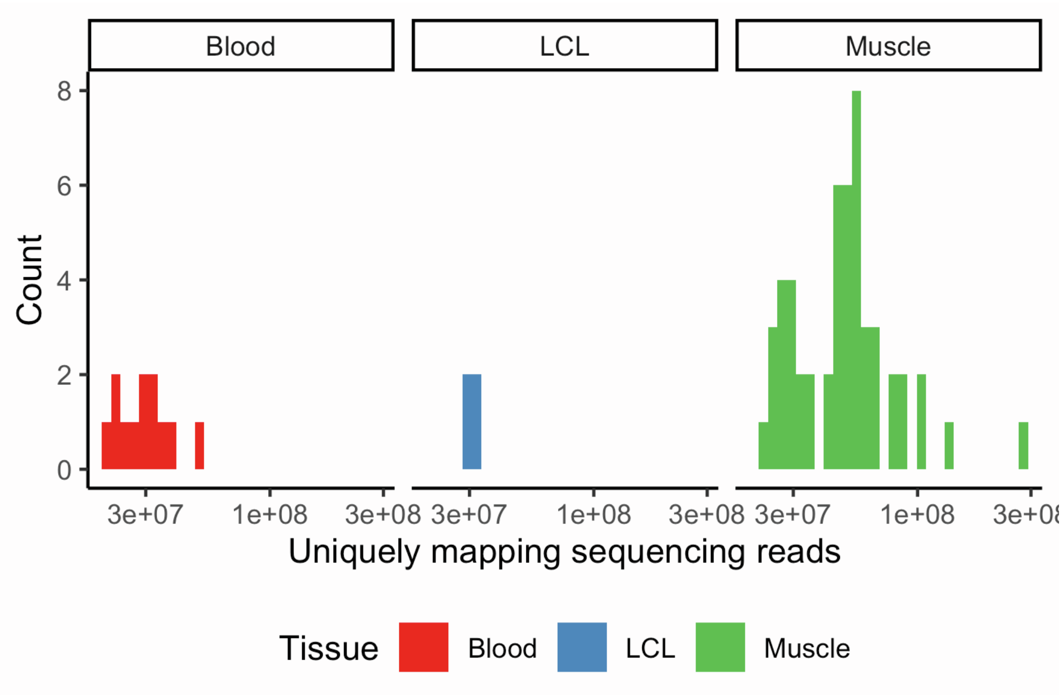

Figure S3. *Sequencing depths of RNA-seq samples used for evaluation of MRSD model accuracy.* Whole blood (*n* = 12), LCL (*n* = 4) and skeletal muscle (*n* = 52) RNA-seq samples were derived from in-house or previously published data (3) for validation of the MRSD model efficacy. Sequencing depths across the three tissues ranged from 20.6-281.5 M uniquely mapping reads.

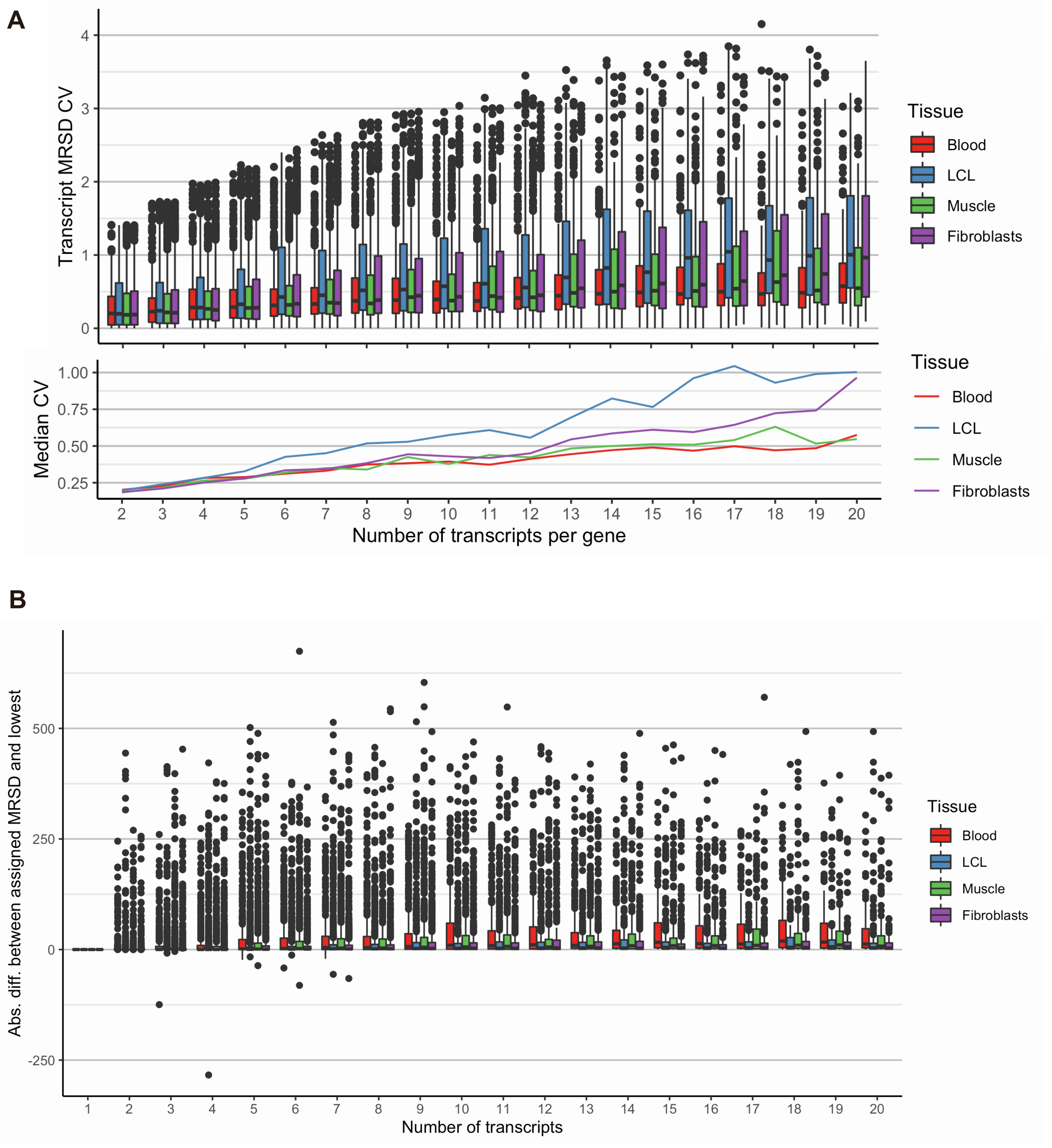

Figure S4. *Extent of variability in MRSD scores among the different transcripts of individual genes.* (**A**) Considering the MRSDs of genes with up to 20 MRSD-feasible GENCODE-annotated transcripts, we observed a median relative variability in MRSD (coefficient of variation, CV) across the four analysed tissues of 0.37-0.49. An increased number of transcripts per gene was associated with a small, gradual increase in CV. (**B**) Where our selected transcript generated an MRSD prediction, we observed only a small median difference in MRSD between this prediction and that of the lowest-MRSD transcript annotated for the same gene (median difference of 1.06-3.65 M reads).

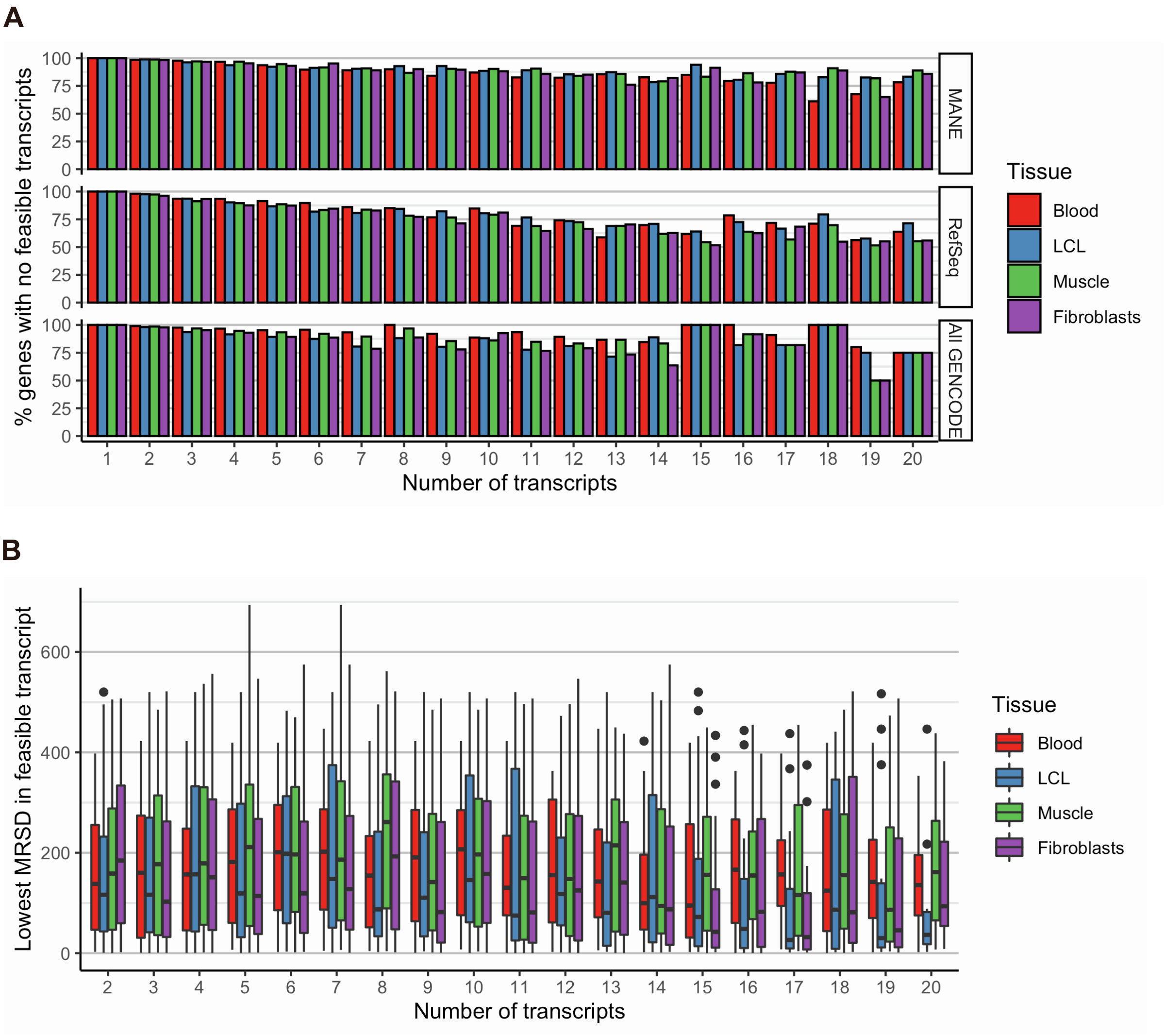

Figure S5. *Transcripts in genes deemed unfeasible through hierarchical selection are themselves likely to be unfeasible.* (**A**) Among genes for which our hierarchically selected transcript is deemed unfeasible through MRSD, 89.05-90.37% with multiple transcripts in GENCODE v19 are predicted to have no feasible transcripts. Of all the transcript tiers, unfeasible RefSeq composite transcripts are most likely to be assigned to genes with at least one feasible transcript. (**B**) In the remaining cases (in which an unfeasible gene is predicted to have at least one feasible transcript), the median MRSD for the lowest-MRSD transcript ranges from 108.59-157.78 M reads, depending on tissue choice.

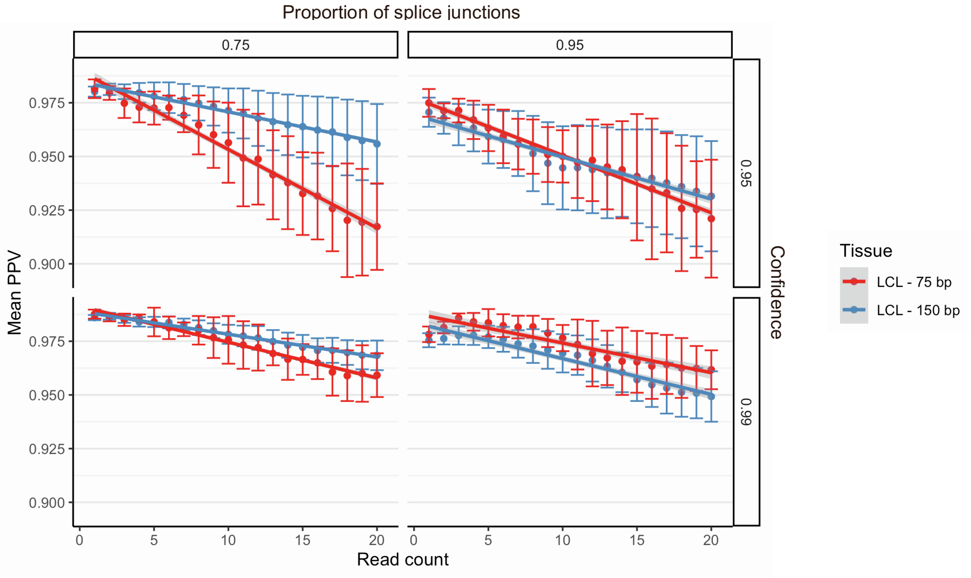

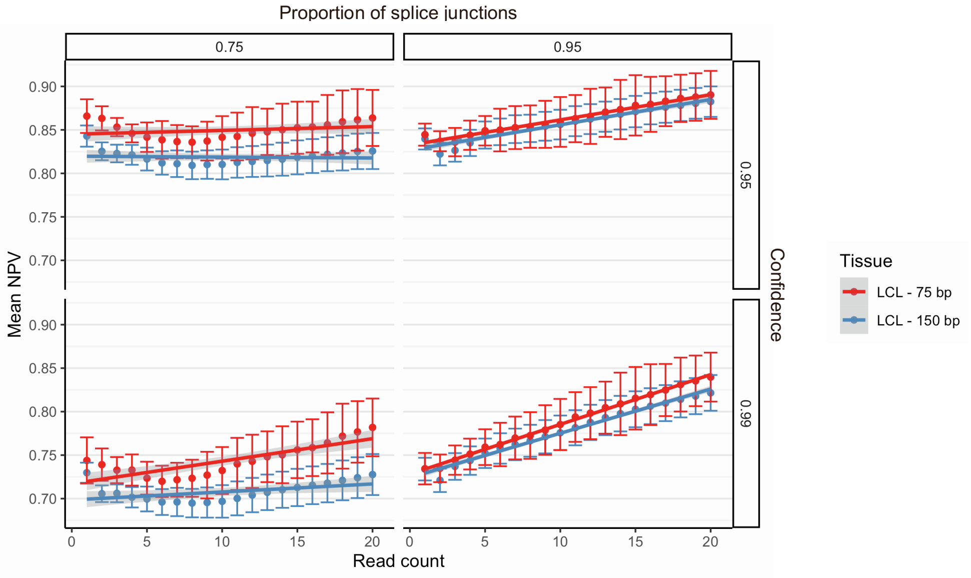

Figure S6. ***Effect of varying sequencing read length on MRSD model performance.*** Despite being derived from 75 bp paired end RNA-seq data, MRSD scores show similar performance when applied to 75 or 150 bp paired end read-based RNA-seq, both in terms of (top) PPV and (bottom) NPV. When specifying 75% splice junction coverage, MRSD PPV is generally higher when the model is applied to 150 bp read-based data. This likely reflects the fact that junctions predicted to be sufficiently covered by 75 bp reads will be more likely to be sufficiently covered by reads of greater length, and so positive predictions are more likely to hold true when applied to longer-read data. We also observe that NPV for 150 bp read datasets is lower than that for 75 bp across all 4 parameter combinations; conversely to PPV, this is possibly because transcripts not sufficiently covered by 75 bp reads are more likely to be sufficiently covered by 150 bp reads, thus making negative predictions less likely to hold true in longer-read data. In most cases, differences in model performance between 75 and 150 bp is low, suggesting MRSD may, in some cases, provide a suitable approximation of transcript coverage in RNA-seq datasets with read lengths different to those used to construct the model.

Figure S7

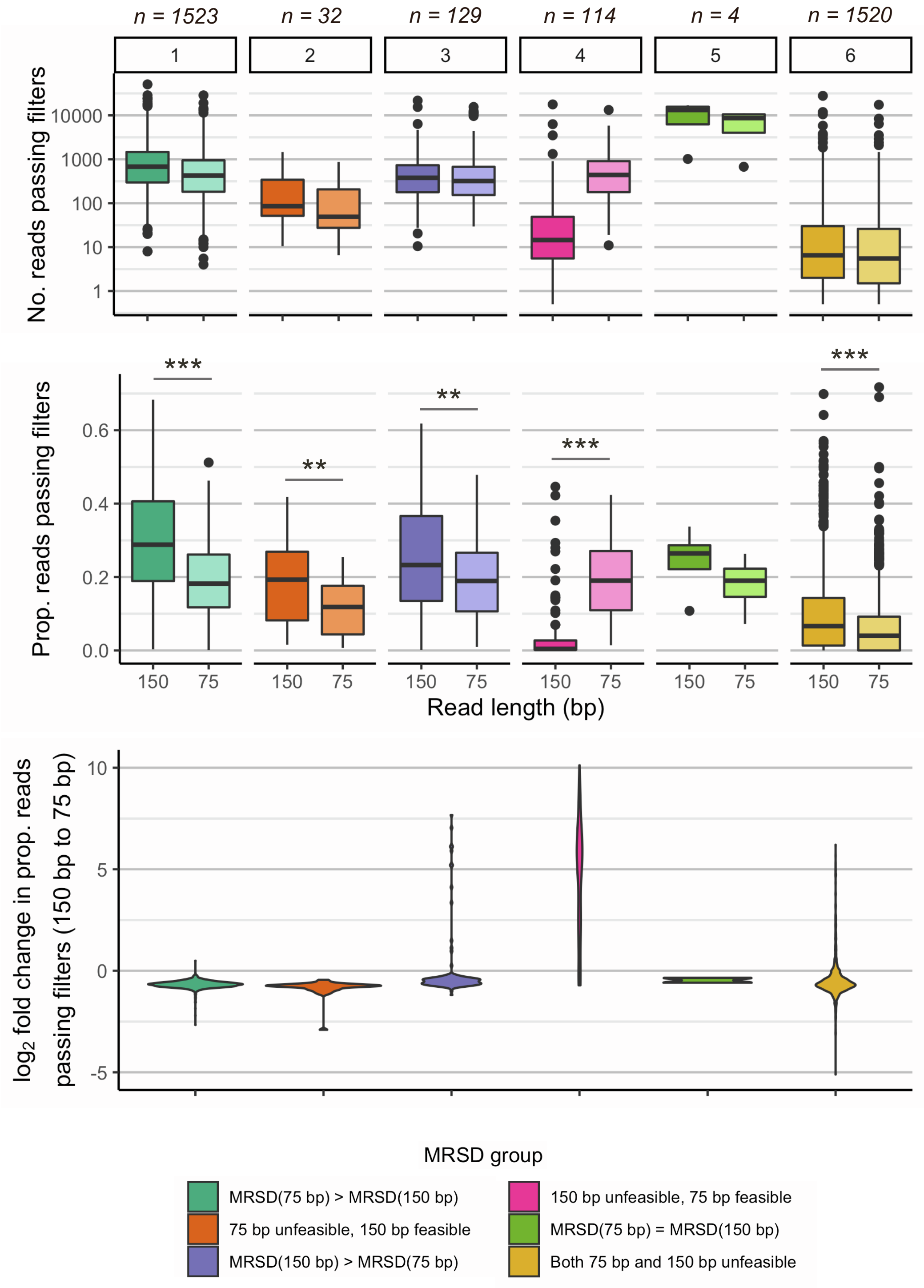
n = 1523
n = 32
n = 129
n = 114
n = 4
n = 1520
1
2
3
4
5
6
No. reads passing filters
10000
1000
100
10
1
Prop. reads passing filters
0.6
0.4
0.2
0.0
***
**
**
***
***
150
75
Read length (bp)
log2 fold change in prop. reads passing filters (150 bp to 75 bp)
10
5
0
-5
MRSD group
MRSD(75 bp) > MRSD(150 bp)
75 bp unfeasible, 150 bp feasible
MRSD(150 bp) > MRSD(75 bp)
150 bp unfeasible, 75 bp feasible
MRSD(75 bp) = MRSD(150 bp)
Both 75 bp and 150 bp unfeasible

Figure S7. *MRSD scores are generally lower when derived from RNA-seq runs of longer read length.* MRSD predictions generated from 20 LCL-based 150 bp RNA sequencing runs were compared against those generated following trimming of the same reads to a maximum of 75 bp. For 45.8% (1520/3322) of disease-associated genes, coverage was too poor to generate an MRSD score regardless of read length (group 6), while MRSDs could be generated but remained the same regardless of read length for just 4/3322 (0.12%) genes (group 5). Intuitively, of the 54.1% (1798/3322) of genes for which at least one dataset allowed MRSD generation, a higher MRSD was observed in the 75 bp dataset for 86.5% (1555/1798, groups 1 and 2). However, for the remaining 13.5% of genes (243/1798, groups 3 and 4), a lower MRSD score was generated using the 75 bp dataset than the 150 bp dataset. For many of these genes, it was determined that a shortening of the reads actually improved their quality to the extent that they were more likely to pass the enforced quality filters – namely, that a mapping event must be the primary alignment, that the read must map successfully (i.e. must have a mapping quality of 60) and that the read must be a split read. We observed that in group 4, comprising genes for which MRSD generation is unfeasible using the 150 bp dataset but feasible using the 75 bp dataset, there was a median 36.8-fold increase in the number of reads passing these read filters following trimming (bottom). Further work is needed to investigate alternative causes of this counter-intuitive pattern, and to determine whether the discarding of the longer reads represents an artefactual drawback to the read filtering process, or an effective way to filter reads for quality that is missed using shorter reads.

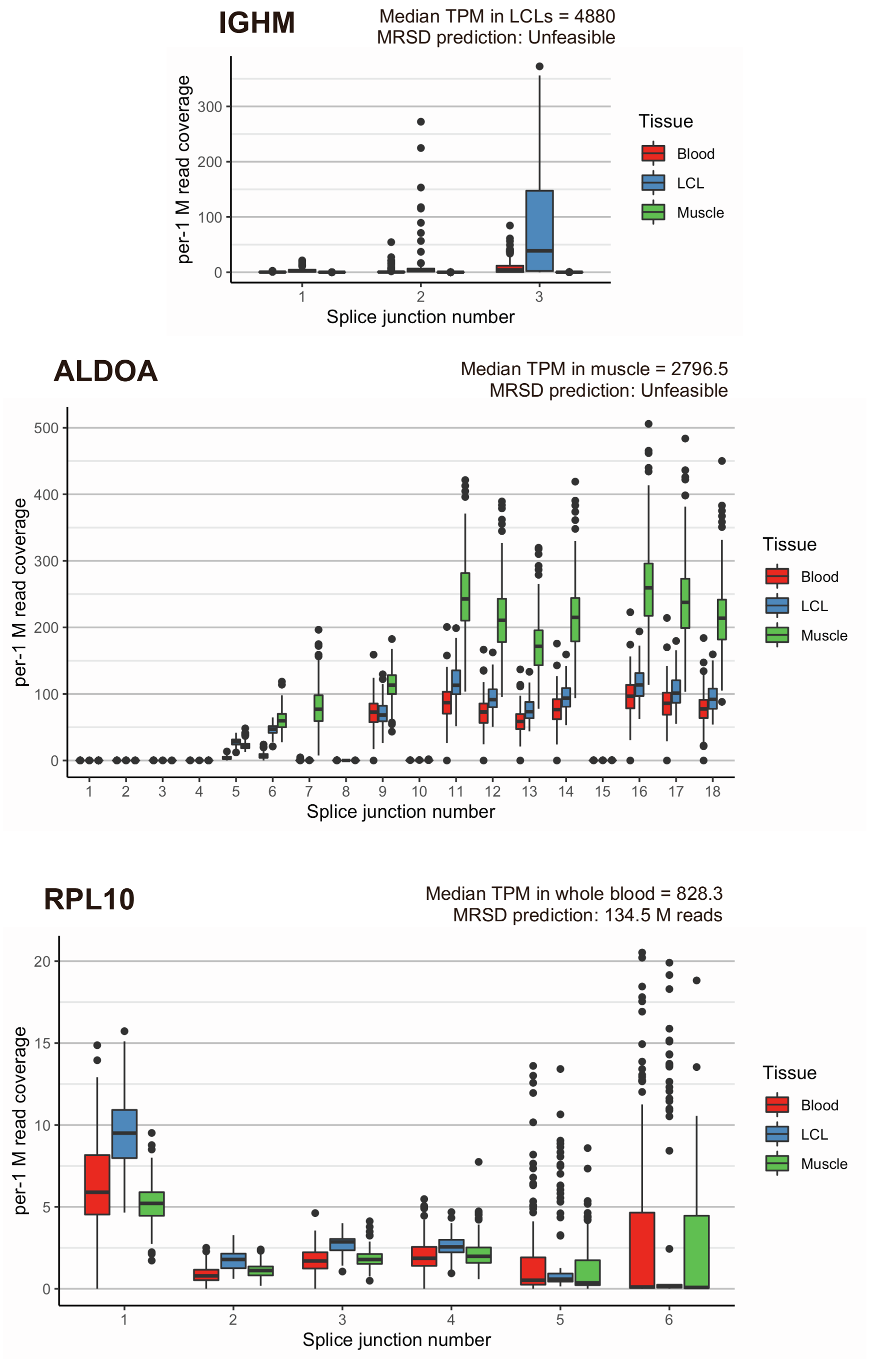

Figure S8. *Evidence for 3' sequence bias confounding the use of TPM as a guiding RNA-seq metric.* Analyzing the number of reads (per 1 M uniquely mapping input reads) mapping to individual splice junctions within three genes with substantial TPM-MRSD discrepancy demonstrates that highly expressed genes may exhibit biased coverage of splice junctions. For IGHM (top) and ALDOA (middle) in LCLs and muscle, respectively, a sufficient proportion of junctions towards the 3' end of the transcript have no read support in a sufficient number of patients, resulting in an MRSD prediction of "unfeasible", despite high coverage of other junctions within the same transcript. Coverage of the final two splice junctions in RPL10 (bottom) in LCL-based RNA-seq data is low but not non-zero in many patients, giving a feasible but high MRSD prediction. In some cases, this bias may result from artefacts of library preparation, or may possible reflect genuine isoform shifts in the given tissue. Higher splice junction numbers represent junctions closer to the 3' end of transcripts.

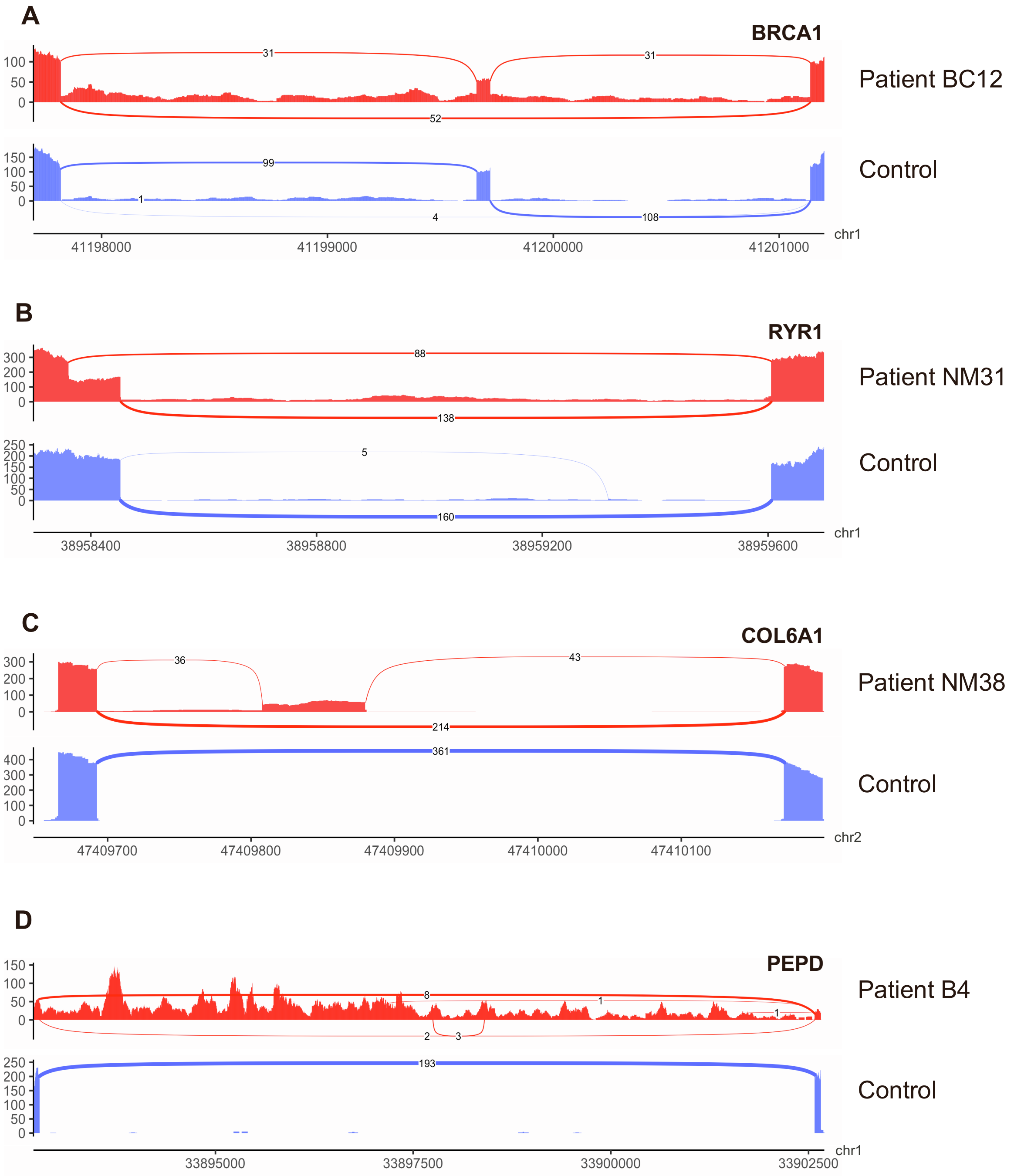

Figure S9. *Exemplar events identified during pathogenic splice event analysis.* Selected Sashimi plots for (**A**) exon skipping, (**B**) exonic splice gain, (**C**) pseudoexonization and (**D**) intron retention events identified as the cause of disease in our patient datasets. The presence of aberrant splice junctions with outlying event metrics allowed flagging of these as potentially pathogenic. For (**D**), the intron retention event was identified from the 2 reads supporting usage of an extremely weak alternative splice acceptor four bases downstream of the abrogated canonical acceptor; however, in the absence of any aberrant splicing events, intron retention events are more difficult to identify from RNA-seq data using current bioinformatics pipelines.

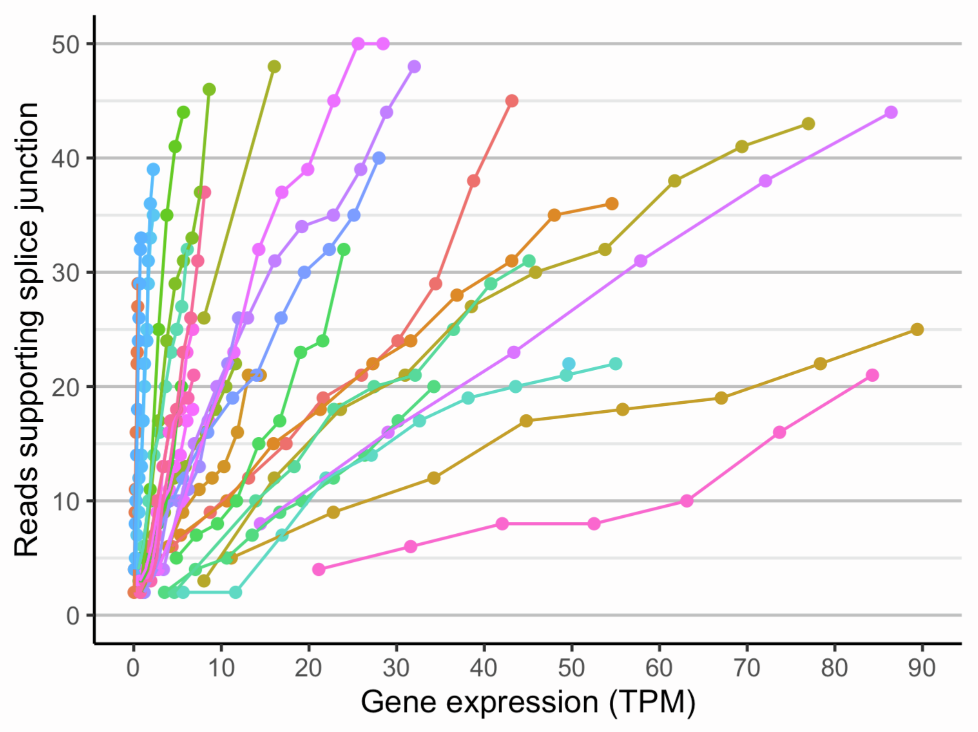

Figure S10. *Relative gene expression level does not reflect the raw read coverage of transcript splice junctions.* When simulating decreased gene expression by downsampling reads in genes containing novel splicing events identified in upstream analysis, it emerged that expression of a gene (in transcripts per million, TPM) does not directly correlate with the number of reads supporting splice junctions in that gene. Among the events supported by 8 reads, for example, gene expression ranged from 0.17-52 TPM. This may be accounted for by variation in the proportion of transcripts containing the event, variation in the coverage across the length of a transcript (as shown in Figure S4), or variation in the depth to which a sample has been sequenced. Thus, when specifying a metric threshold above which we expect splice aberration to be observable, relative expression level may not appropriately represent expected read support. Axes are limited for ease of visualization.

Figure S11. *Pairwise comparisons, by tissue, of predicted MRSD scores for PanelApp disease genes.*

A) MRSD predictions in muscle vs. blood

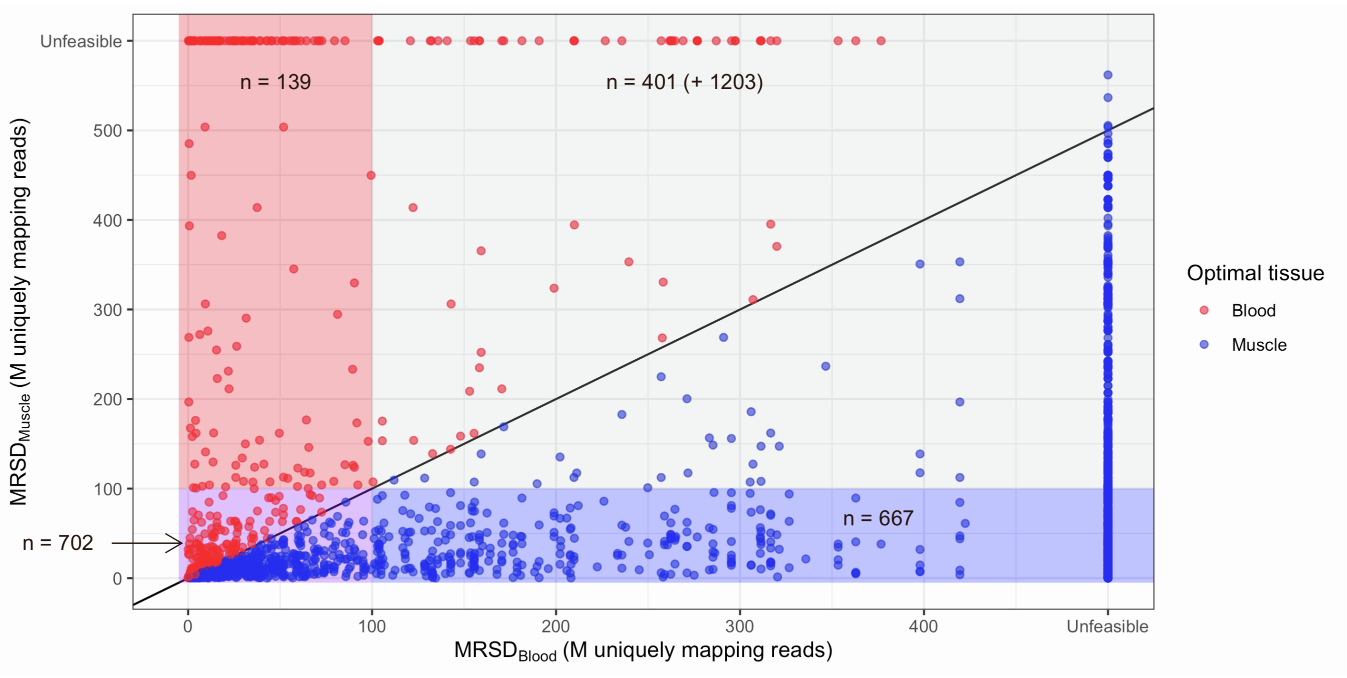

B) MRSD predictions in LCLs vs. muscle

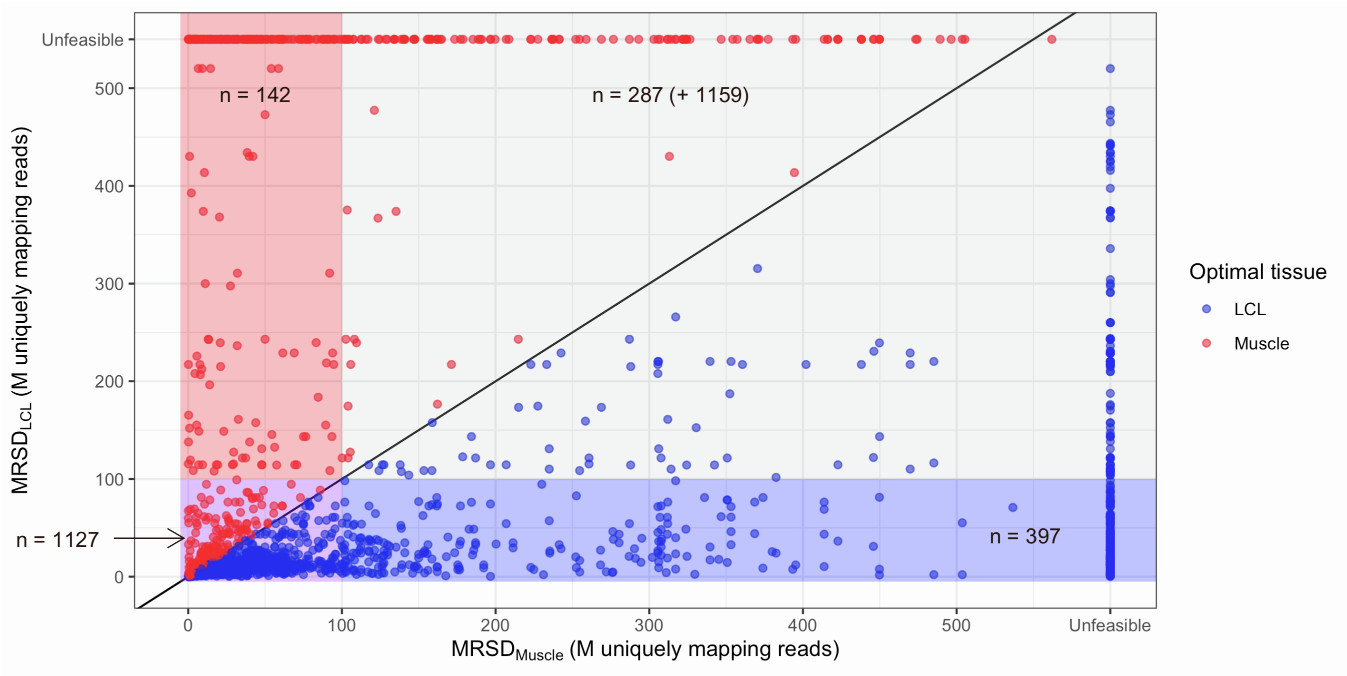

Figure S12. *Proportion of low-MRSD genes per tissue for all PanelApp panels, ordered by panel size.*

A) Low-MRSD gene proportions for large panels (> 50 genes)

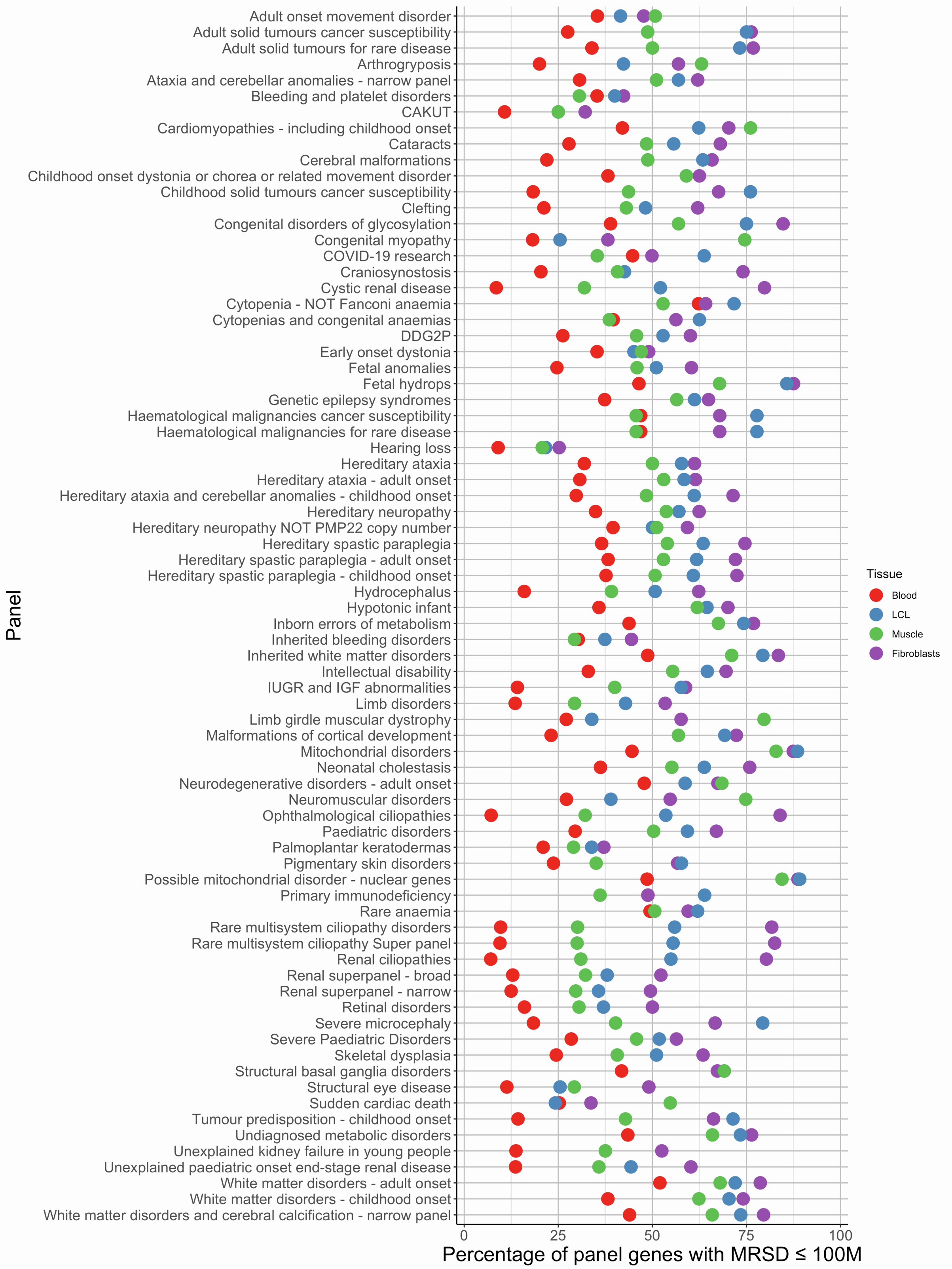

B) Low-MRSD gene proportions for medium panels (21-50 genes)

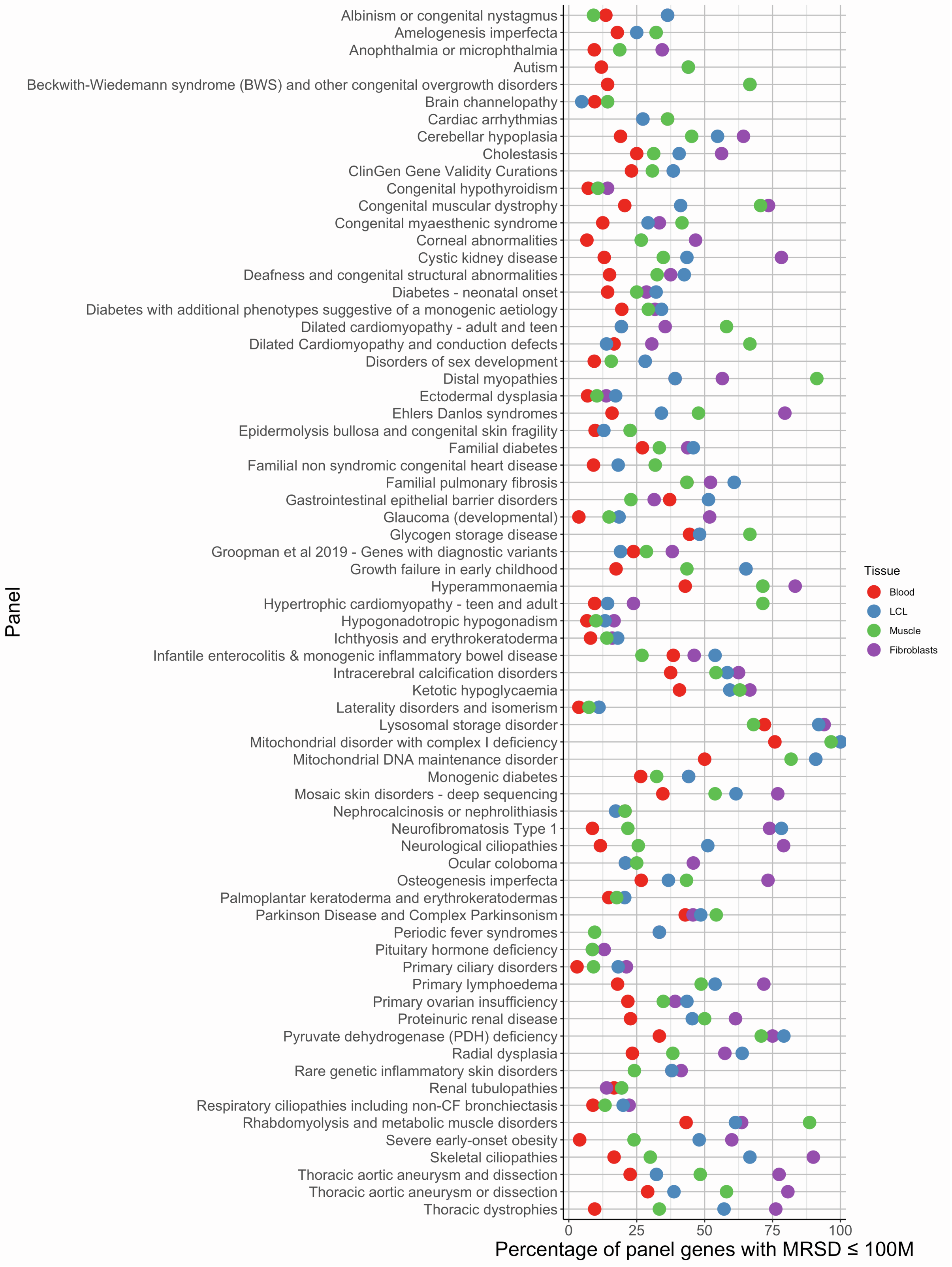

C) Low-MRSD proportions for small panels (11-20 genes)

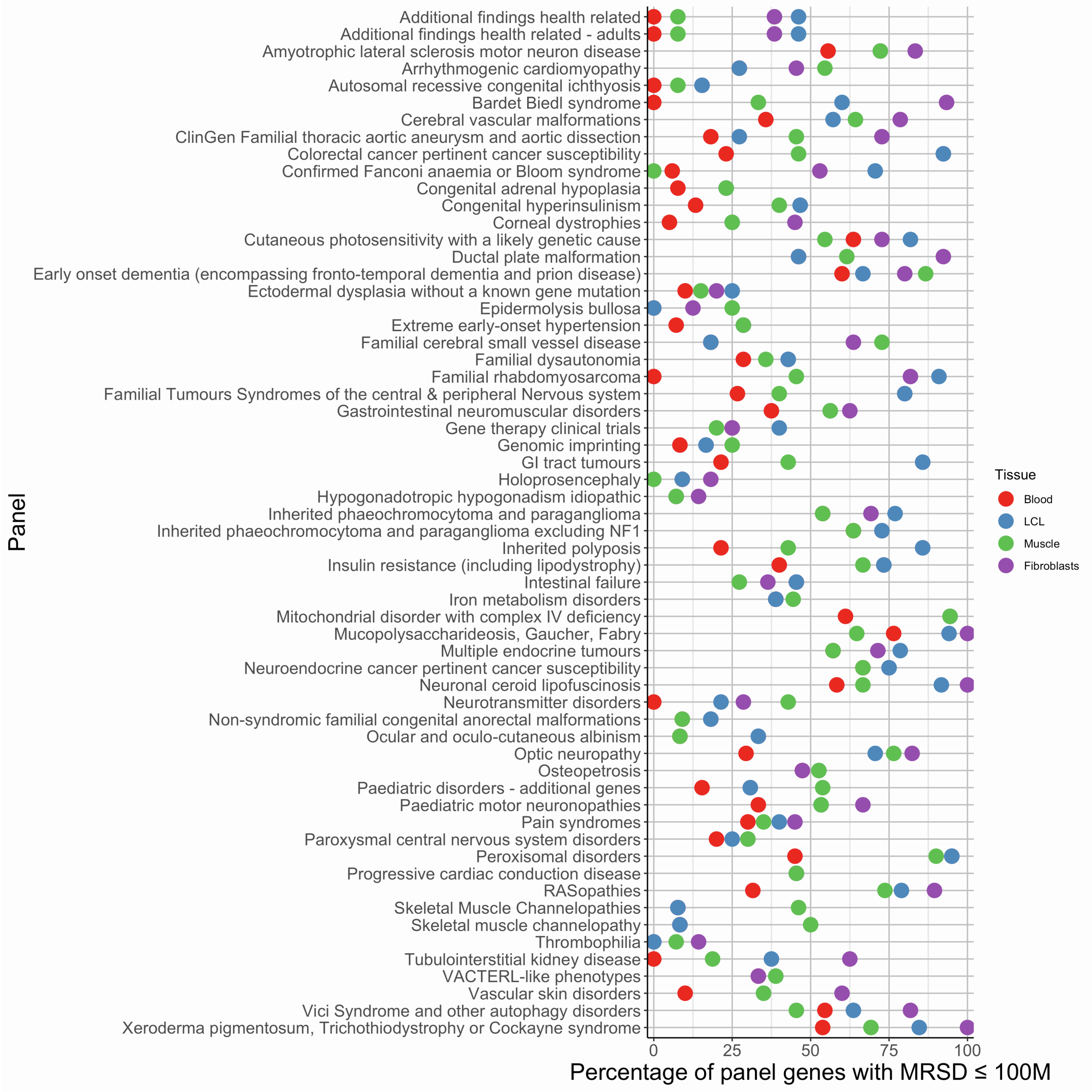

D) Low-MRSD gene proportions for very small panels (≤ 10 genes)

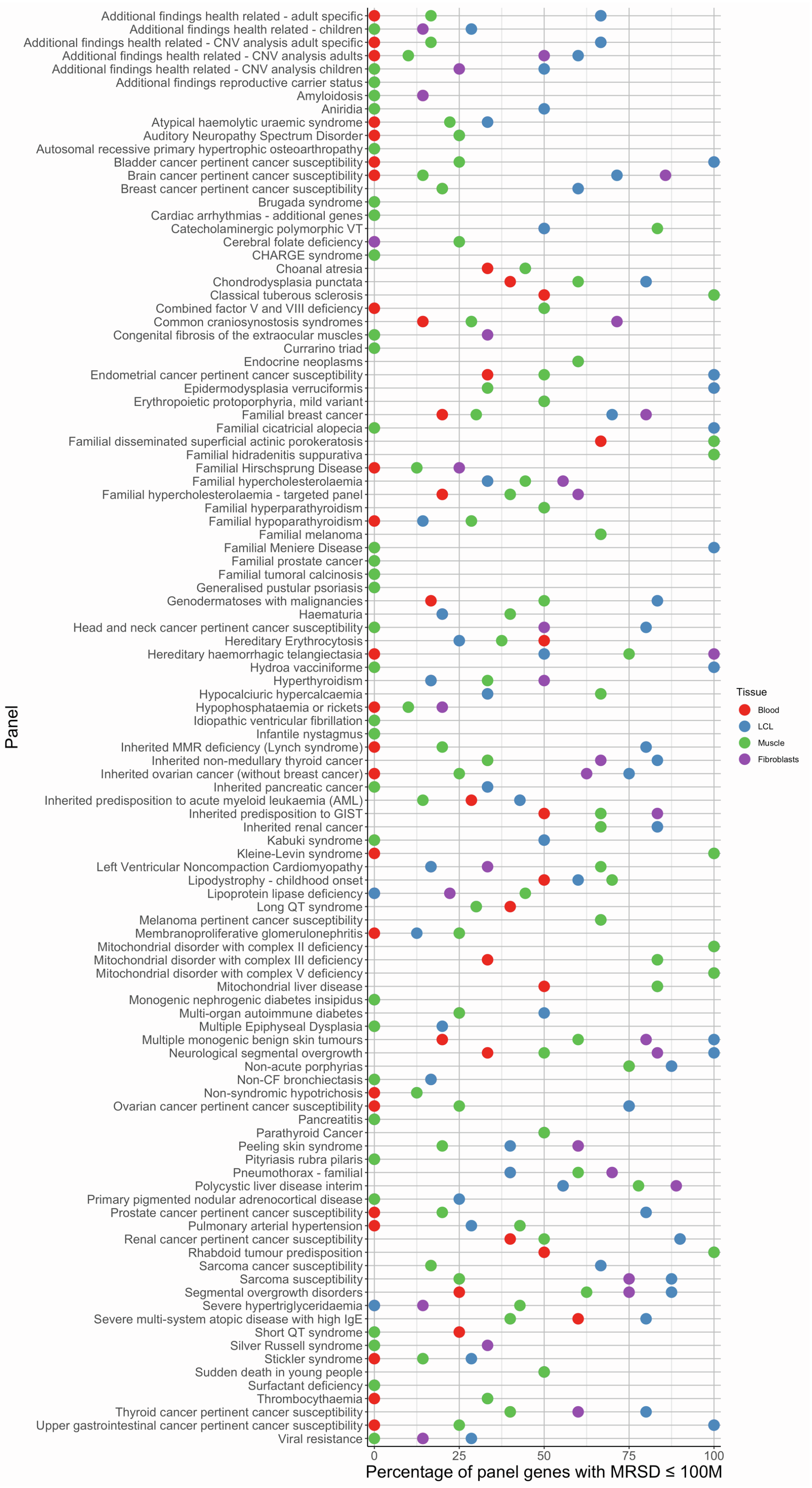

Figure S13. *Proportion of low-MRSD genes per tissue for all PanelApp panels, ordered alphabetically by panel name.*

A) Low-MRSD gene proportions for panels named A-E

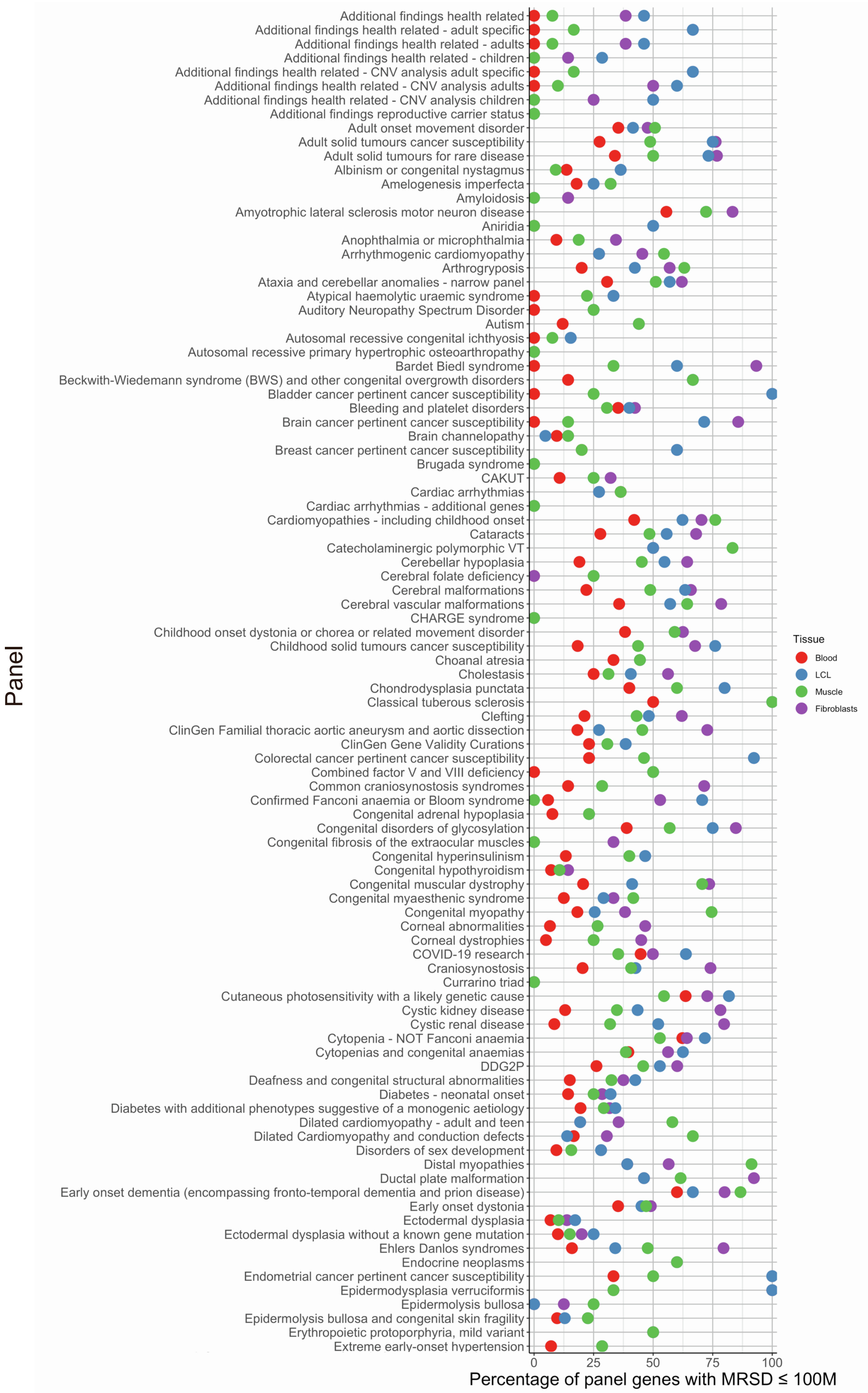

B) Low-MRSD proportions for panels named F-L

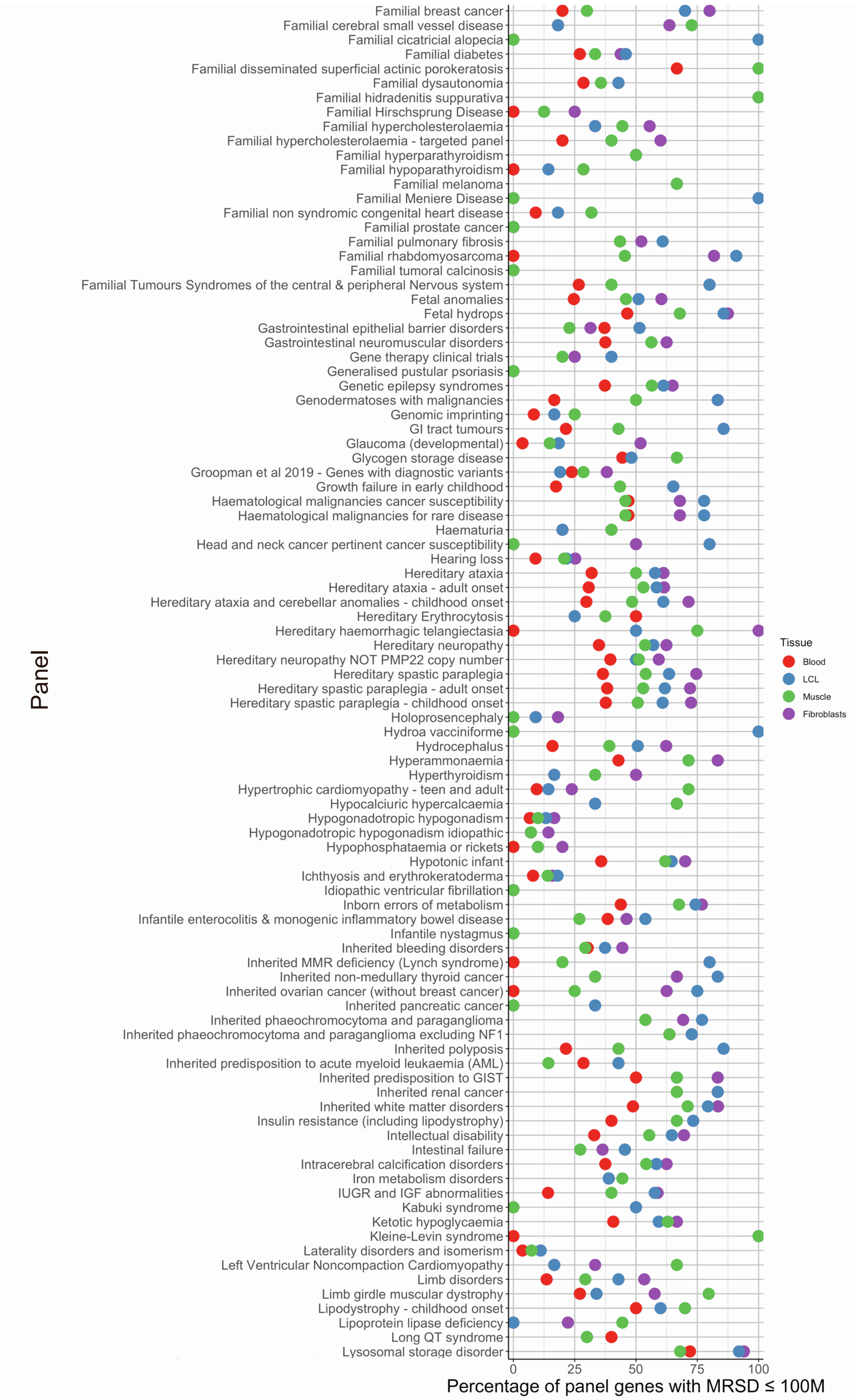

C) Low-MRSD gene proportions for panels named M-R

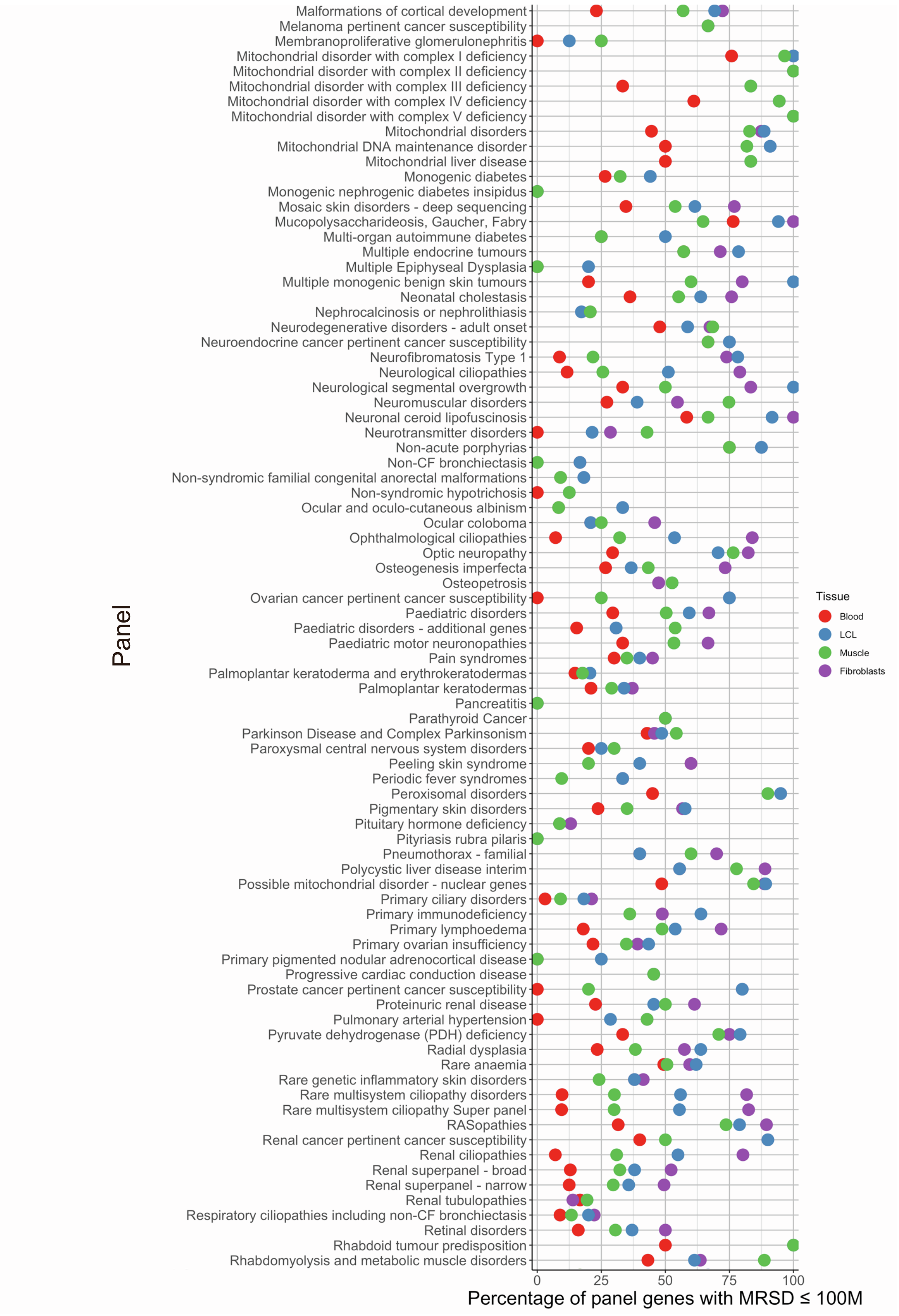

D) Low-MRSD gene proportions for panels named S-X

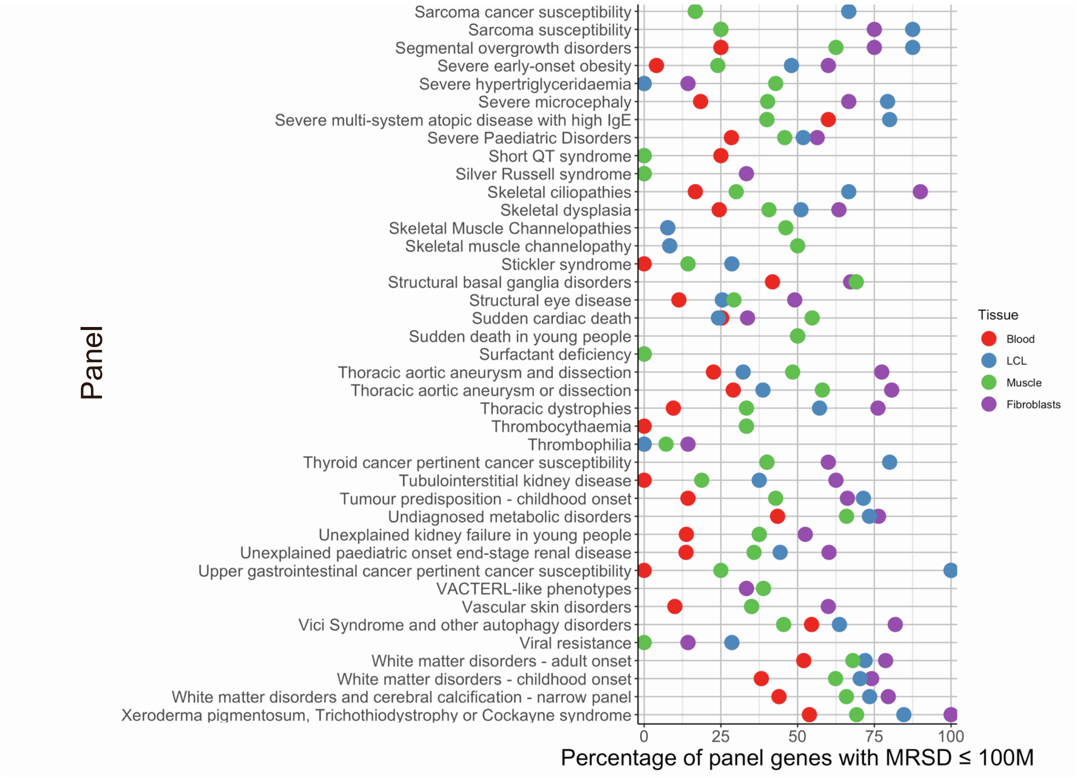

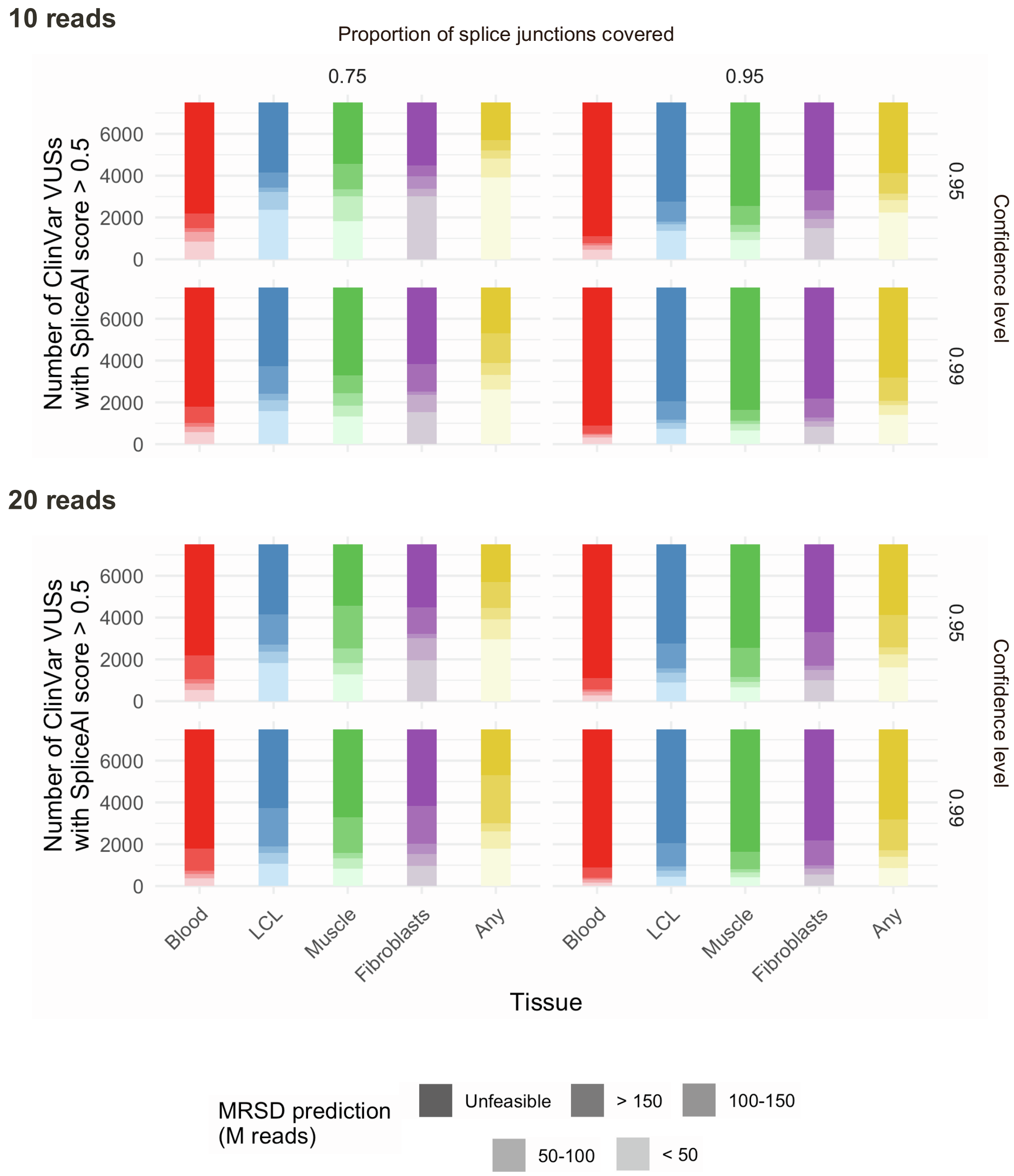

Figure S14. *Increasing specified read coverage reduces the number of ClinVar variants that can be analyzed.* Similarly to Figure 7a (main text), we generated MRSD scores for genes harboring predicted splice-impacting ClinVar variants (SpliceAI score ≥ 0.5 (4)) using more stringent read coverage parameters (10 and 20 reads). We observed only a small reduction in the number of ClinVar variants in low-MRSD genes when specifying 10 reads (24.9-64.0% dependent on parameters). Specifying 20 read coverage, however, dramatically reduces the percentage of ClinVar variants in low-MRSD genes to 18.7-52.0%.

10 reads

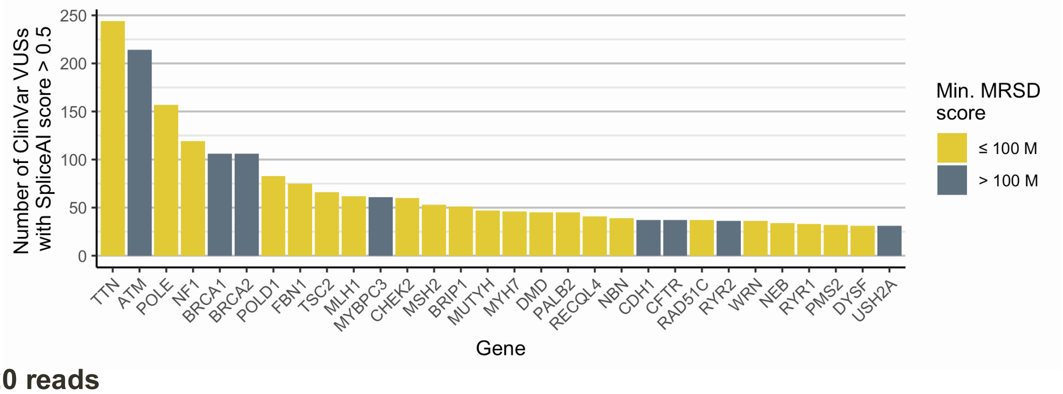

20 reads

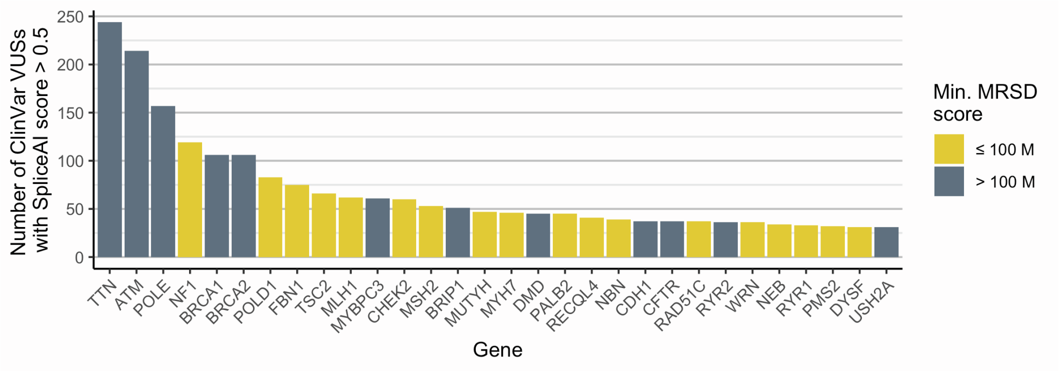

Figure S15. *Increasing specified read count removes highly VUS-prone genes from the scope of analysis.* Similarly to Figure 7b, we looked among the 30 genes harboring the most predicted splice-impacting ClinVar variants and considered how many were low-MRSD in at least one of the four investigated tissues when specifying increasing levels of read coverage. Only one extra gene, ATM, becomes ostensibly high-MRSD when specifying a 10-read coverage parameter when compared with the 8-read coverage data (Figure 7b). However, by specifying a 20-read level of coverage, a further four genes are removed from the scope of analysis, leaving 18/30 (60%) still considered low-MRSD.

Variant (HGVSg)	Gene	Source of RNA	Phenotype	TPM	MRSD (M reads)
chr2:152,355,017G>T	NEB	Skeletal muscle	Nemaline myopathy	857.9	9.83
chr2:152,389,953A>C	NEB	Skeletal muscle	Nemaline myopathy	857.9	9.83
chr2:152,544,805C>T	NEB	Skeletal muscle	Nemaline myopathy	857.9	9.83
chrX:31,790,694-31,798,498invdel	DMD	Skeletal muscle	Duchenne muscular dystrophy	24.84	79.4
chrX:32,274,692G>A	DMD	Skeletal muscle	Myalgia, myoglobinuria	24.84	79.4
chr2:179,446,219ATACT>A	TTN	Skeletal muscle	Fetal akinesia	349.5	47.63
chr2:179,642,185G>A	TTN	Skeletal muscle	Multi/minicore congenital myopathy	349.5	47.63
chr21:47,409,881C>T	COL6A1	Skeletal muscle	Collagen VI-related dystrophy	56.02	16.25
chr21:47,409,881C>T	COL6A1	Skeletal muscle	Collagen VI-related dystrophy	56.02	16.25
chr19:38,958,362C>T	RYR1	Skeletal muscle	Congenital fiber-type disproportion	425.5	3.45
chr1:46,655,129C>A	POMGNT1	Skeletal muscle	α-Dystroglycanopathy	29.26	6.01
chr17:41,199,655C>G	BRCA1	LCL	Inherited breast cancer susceptibility	19.985	217.19
chr17:41,246,879T>C	BRCA1	LCL	Inherited breast cancer susceptibility	19.985	217.19
chr17:41,246,879T>C	BRCA1	LCL	Inherited breast cancer susceptibility	19.985	217.19
chr17:41,246,879T>C	BRCA1	LCL	Inherited breast cancer susceptibility	19.985	217.19
chr17:41,258,551C>A	BRCA1	LCL	Inherited breast cancer susceptibility	19.985	217.19
chr13:32,945,238G>A	BRCA2	LCL	Inherited breast cancer susceptibility	10.16	Unfeasible
chr13:32,969,074A>T	BRCA2	LCL	Inherited breast cancer susceptibility	10.16	Unfeasible
chr19:33,892,776C>T	PEPD	Whole blood	Prolidase deficiency	18.89	28.31
chr20:35,526,363C>G	SAMHD1	Whole blood	Aicardi-Goutières syndrome	48.53	24.68
chr23:153,997,595G>A	MED13L	Whole blood	MRFACD	5.89	262.34

Table S1. *Summary of pathogenic splicing events analyzed in this study.* All co-ordinates are given in relation to the GRCh37 genome build. TPM, transcripts per million; MRSD, minimum required sequencing depth.

Tissue	No. samples	Source	Sequencing type	Usage
Blood	151	GTEx	75-bp paired end poly-A enrichment, Illumina	Generation of MRSD model, bootstrapping analysis of event counts
LCL	91			
Muscle	184			
Blood	1	Inhouse	150-bp paired end globin depletion, Illumina	Collation of known pathogenic mis-splicing events
	12		75-bp paired end poly-A enrichment, Illumina	Collation of known pathogenic mis-splicing events & MRSD model validation
LCL	20		150-bp paired end poly-A enrichment, Illumina	Collation of known pathogenic mis-splicing events
	4	Inhouse	75-bp paired end poly-A enrichment, Illumina	MRSD model validation
Muscle	52	Previously published data (3)	75-bp paired end poly-A enrichment, Illumina	Collation of known pathogenic mis-splicing events, downsampling of pathogenic events & MRSD model validation

Table S2. *Summary of RNA-seq datasets utilized in this study*. RNA-seq datasets derived using different methodologies were used for various aspects of this study. All data used to generate the MRSD model was based on data from the GTEx consortium across all three analyzed tissues.

Methods S1.

Minimum required sequencing depth (MRSD) score (further elaboration).

MRSD is defined for an individual transcript in a given sample as:

$$MRSD_m = r/\left(\frac{R_p}{d}\right)$$

Where r is the desired level of read coverage across desired proportion p of splice junctions, R is the set of read counts supporting each of the splice junctions in the transcript of interest, ordered from lowest to highest, and R_p is the read count at the position in R at which proportion p of read counts values in R are greater than or equal to it. d represents the total number of sequencing reads, in millions of reads, in the RNA-seq sample (by default, the number of uniquely mapping sequencing reads), and (m) represents the MRSD parameter.

For instance, suppose a sample sequenced to a depth (d) of 40 M uniquely mapping sequencing reads generates coverage of 14, 16, 6 and 10 reads across the splice junctions of a five-exon transcript. Suppose we wish 75% of splice junctions to be covered by a minimum of 6 reads (i.e. p = 0.75 and r = 6). Here, R = {6, 10, 14, 16} and R_p = 10, as 3/4 (75%, i.e. p) of all values in R are greater than or equal to 10. Inserting these values into the formula shows that this transcript has an MRSD of $\frac{6}{10/_{40}} = 24\,M$ uniquely mapping sequencing reads in this sample.

The set of MRSD scores for the given transcript are then collated across all control samples and ordered from lowest to highest. The score at the m-th percentile position in the collated list of sample-specific MRSDs is returned as the overall MRSD for that transcript, where m is termed the "MRSD parameter" and is customizable by the user (default = 0.95). The $MRSD_{0.99}$ of a transcript represents the sequencing depth that would be required for 99% of control samples to achieve the specified coverage for that transcript. The MRSD parameter therefore approximately represents the likelihood that a sequencing run at the returned depth will yield the desired coverage level.

Illustration of MRSD calculation methodology. MRSD scores utilize the level of read coverage supporting the existence of splice junctions in control RNA-seq datasets to predict the depth of sequencing required to achieve a specified level of splice junction coverage in a transcript of interest. For a given transcript in a given individual:

1. Read coverage values are collated across all splice junctions in the transcript model (with a single transcript assigned to each gene if investigating at the gene level, see Methods S2, below)
2. Each of these values is divided by the sequencing depth – by default defined as the number of uniquely mapping sequencing reads (in millions of reads) to produce a per-1 M read coverage value for each junction
3. The desired level of read coverage is divided by the per-1 M read coverage value of the splice junction with the *X*'th percentile lowest read coverage, which gives the depth of sequencing that would be required for X% of junctions to be covered with the desired number of reads or higher. This figure is the sample-specific MRSD.

The sample-specific MRSDs are collated across all control RNA-seq samples, and a global MRSD is then derived by taking the m-th percentile highest prediction from among these; m is termed the MRSD parameter, and represents the proportion of control RNA-seq samples for which sequencing at the returned MRSD would have sufficiently covered that gene. By

extension, it is also an approximate measure of the likelihood that a subsequent RNA-seq run at the returned depth will yield the specified coverage.

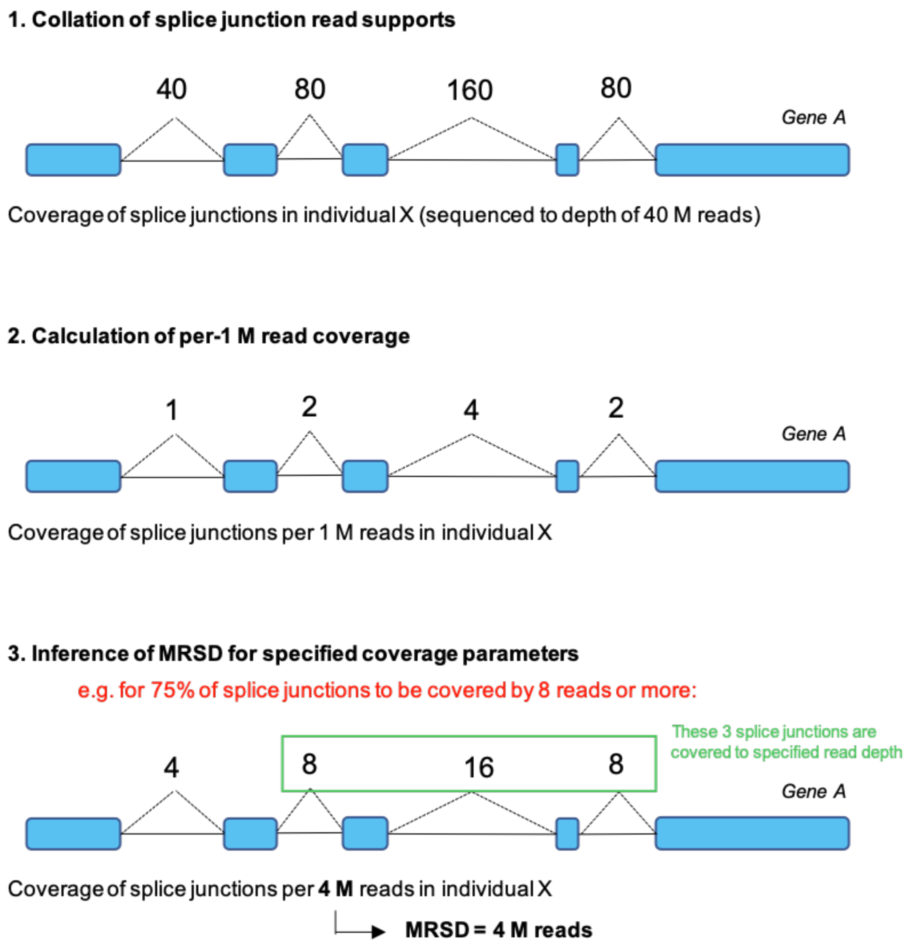

Methods S2. *Tiering methodology for selection of transcripts for MRSD generation.* To calculate MRSD values for all protein-coding genes, a single transcript model was established for each gene. Firstly, transcripts present in the MANE v0.7 curated transcript set were selected for genes where these existed, provided the co-ordinates of all splice junctions in that transcript (given in relation to the GRCh38 reference genome) mapped back to known junctions in build GRCh37. For genes where these conditions were not met, transcript models were formed from the union of all junctions present in all RefSeq transcripts listed for that gene on Ensembl BioMart. Finally, for any genes lacking a corresponding RefSeq transcript(s), a transcript model was derived consisting of the union of all junctions present in all transcripts assigned to that gene in the GENCODE v19 annotation.

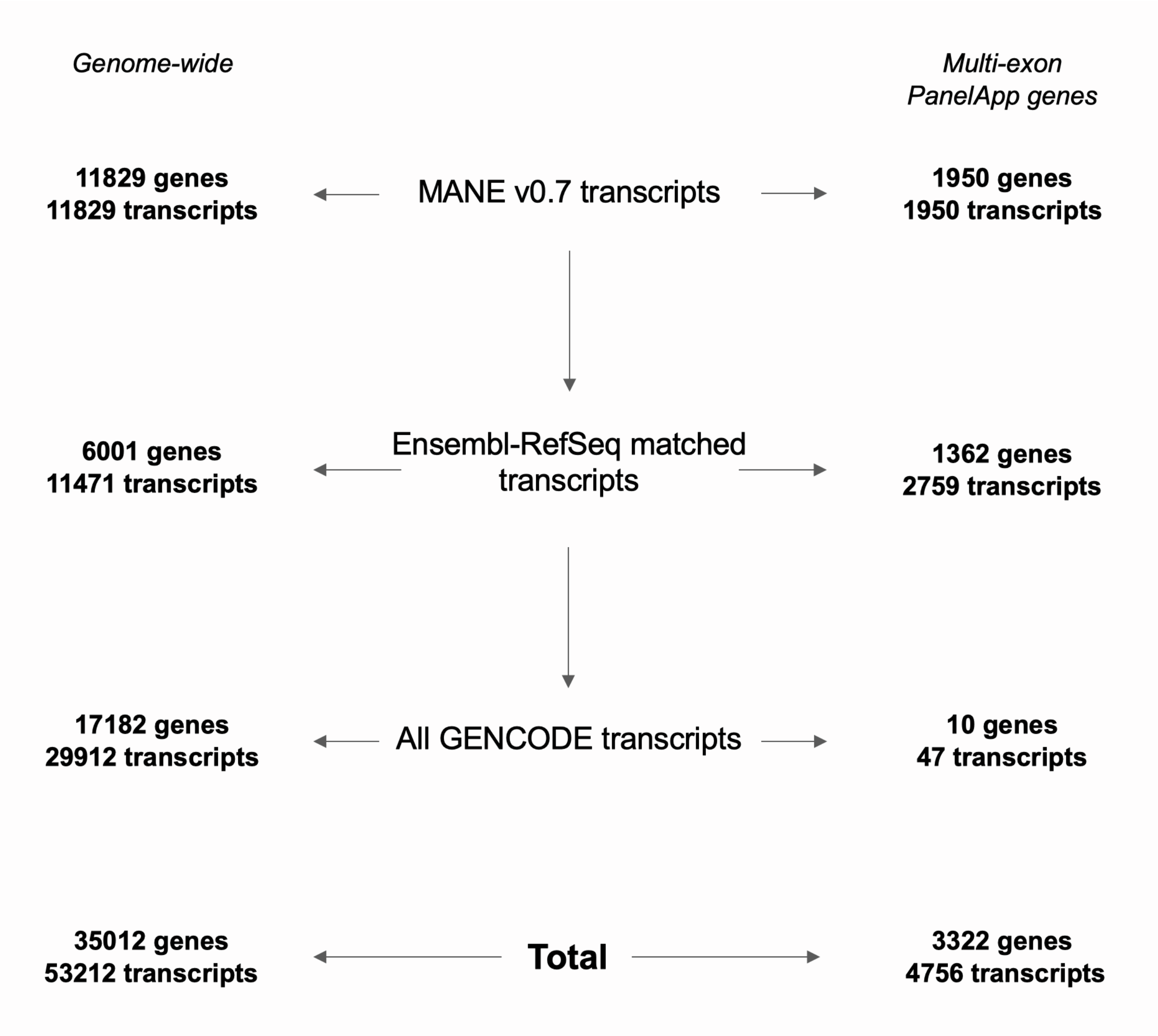

Methods S3. *Tissue-specific criteria for filtering of high-quality GTEx control RNA-seq datasets.* Filtering of GTEx controls was conducted to select the highest quality samples based on the below tissue-specific parameters. Parameters were selected and adjusted on a tissue-by-tissue basis to exclude metric outliers and samples that may confound analysis of pathogenic splicing events (e.g. excluding cancer patients from LCL control cohorts, in which inherited breast cancer was studied). The corresponding column names in the GTEx v8 sample attribute (pht002743.v8) and subject phenotype (pht002742.v8) files are italicized.

Skeletal muscle (as listed in [1])

- RNA integrity number/RIN (*SMRIN*): between 6-9
- Sample ischemic time (*SMTSISCH*): <720 (i.e. <12 hours)
- Hardy scale (*DTHHRDY*): 0, 1 or 2, corresponding to sudden deaths
- Age (*AGE*): <50
    - Unless BMI <30

Whole blood

- Samples included in GTEx analysis freeze, corresponding to higher quality samples (*SMAFRZE*): not flagged EXCLUDE due to technical issues
- RIN (*SMRIN*): between 6-9
- Sample ischemic time (*SMTSISCH*): <0
- Hardy scale (*DTHHRDY*): 0, 1 or 2

EBV-transformed lymphocytes (LCLs)

- *SMAFRZE*: not flagged EXCLUDE due to technical issues
- RIN (*SMRIN*): > 9
- *MHCANCER5*, *MHCANCERC* and *MHCANCERNM* all 0 to eliminate all non-metastatic cancers and all cancers in the past 5 years or current
- *DTHHRDY*: 0, 1 or 2
- No reported history (*MHGENCMT*) of:
    - Breast cancer
    - Ovarian cancer
    - Pancreatic cancer
    - Prostate cancer
    - Colorectal cancer
    - No patients filtered out through this criterion

Cultured fibroblasts

- As for EBV-transformed lymphocytes, except with the addition of the following:
    - RIN (*SMRIN*) > 9.7
    - Uniquely mapping reads (*MPPDUN*): > 60 M

Methods S4. *Sample IDs of GTEx samples used to generate control datasets.*

Skeletal muscle *(as listed in [1])*

GTEX-111CU-2026
GTEX-111YS-2326
GTEX-1122O-2426
GTEX-113JC-2726
GTEX-117YX-2526
GTEX-11DXX-2726
GTEX-11DXZ-2426
GTEX-11EM3-2126
GTEX-11EMC-2626
GTEX-11EQ9-2126
GTEX-11I78-2426
GTEX-11LCK-1226
GTEX-11NSD-2026
GTEX-11P81-2526
GTEX-11P82-1826
GTEX-11VI4-1926
GTEX-11WQC-2626
GTEX-11WQK-0726
GTEX-11XUK-2226
GTEX-11ZTT-2626
GTEX-11ZVC-2726
GTEX-1211K-2126
GTEX-12BJ1-2526
GTEX-12C56-1926
GTEX-12WSJ-1726
GTEX-12WSN-2526
GTEX-12ZZX-0326
GTEX-12ZZY-0626
GTEX-13111-2226
GTEX-1314G-1726
GTEX-131XF-2326
GTEX-131XG-2326
GTEX-132AR-1026
GTEX-132NY-0726
GTEX-1339X-2426
GTEX-133LE-2026
GTEX-1399Q-2426
GTEX-1399R-2526
GTEX-1399S-2726
GTEX-1399U-2526
GTEX-139D8-0726
GTEX-139UW-2626
GTEX-139YR-2526
GTEX-13CF3-1826
GTEX-13D11-2526
GTEX-13FH7-2126
GTEX-13FHO-0726
GTEX-13FTW-2326
GTEX-13FTY-0226
GTEX-13FXS-0326
GTEX-OIZH-1626
GTEX-OOBJ-1626
GTEX-P4PP-1626
GTEX-P4PQ-1626
GTEX-P78B-1626
GTEX-POMQ-1926-SM-3NB1Y
GTEX-POYW-0526-SM-2XCEY
GTEX-PSDG-0426
GTEX-PWCY-2026
GTEX-Q2AH-1826-SM-2S1Q2
GTEX-Q734-2026-SM-3GADA
GTEX-QCQG-2126-SM-2S1P8
GTEX-QDVN-2426-SM-2S1Q4
GTEX-QV44-2026-SM-2S1RD
GTEX-R53T-1826-SM-3GIJX
GTEX-R55D-0626-SM-3GAD5
GTEX-S32W-2326-SM-2XCAW
GTEX-S33H-2226
GTEX-S7SF-2026-SM-3K2AS
GTEX-SNMC-1426-SM-2XCFM
GTEX-SUCS-1626-SM-32PLS
GTEX-T5JC-0626-SM-3NMA6
GTEX-T5JW-1826-SM-3GAE1
GTEX-TKQ2-0826-SM-33HB6
GTEX-TML8-1826-SM-32QOR
GTEX-TMMY-0426-SM-33HBB
GTEX-U3ZG-0326-SM-47JXN
GTEX-U3ZH-1926-SM-4DXTR
GTEX-U3ZM-1226-SM-3DB9G
GTEX-U4B1-1626-SM-3DB8N
GTEX-UJHI-1726-SM-3DB9B
GTEX-UJMC-1826-SM-3GADT
GTEX-VUSG-2626-SM-4KKZI
GTEX-WHPG-2226-SM-3NMBO
GTEX-WHSB-1826-SM-3TW8M
GTEX-WOFM-1326-SM-3MJFR
GTEX-WRHK-1626-SM-3MJFH
GTEX-WRHU-0826-SM-3MJFN
GTEX-WXYG-2526-SM-3NB3F
GTEX-WY7C-2526-SM-3NB2N
GTEX-WZTO-0826-SM-3NM8Q
GTEX-X4XY-0626-SM-4E3IN
GTEX-X638-0326-SM-47JY1
GTEX-X88G-0326-SM-47JZ4
GTEX-XBEC-0626
GTEX-XBED-2626-SM-4E3J5
GTEX-XBEW-1026
GTEX-XOTO-0526-SM-4B662
GTEX-XPT6-2026-SM-4B64V
GTEX-XQ8I-0626-SM-4BOPT

GTEX-13JUV-2326
GTEX-13N11-2726
GTEX-13N2G-2326
GTEX-13NZ9-0626
GTEX-13NZB-2626
GTEX-13O61-2326
GTEX-13OVG-2126
GTEX-13OVH-0626
GTEX-13OVI-1726
GTEX-13OW6-0626
GTEX-13PL7-0626
GTEX-13PVR-2526
GTEX-13QBU-2426
GTEX-13QJ3-0726
GTEX-13S7M-0326
GTEX-13S86-2326
GTEX-13U4I-1826
GTEX-13VXT-0326
GTEX-13W3W-2626
GTEX-13W46-0726
GTEX-13YAN-0526
GTEX-144GL-0326
GTEX-144GM-2026
GTEX-144GN-2426
GTEX-145LT-1626
GTEX-145LV-2326
GTEX-145ME-2026
GTEX-145MI-0326
GTEX-145MN-2426
GTEX-146FH-0526
GTEX-146FQ-0326
GTEX-147F3-0226
GTEX-1497J-2626
GTEX-14A6H-2826
GTEX-14AS3-2126
GTEX-14BMV-0326
GTEX-14C39-2426
GTEX-14ICL-1926
GTEX-O5YT-1626-SM-32PK6
GTEX-OHPK-1626-SM-2YUN3
GTEX-OHPL-1626
GTEX-OHPM-1626
GTEX-XUJ4-2626-SM-4BOQ3
GTEX-XUW1-0826-SM-4BOP6
GTEX-XUYS-0326-SM-47JX2
GTEX-XUZC-2126-SM-4BRW8
GTEX-XV7Q-2926-SM-4BRUL
GTEX-XYKS-2426-SM-4AT43
GTEX-Y114-2526
GTEX-Y3IK-2626
GTEX-Y5LM-2126
GTEX-Y5V5-2526
GTEX-Y5V6-2626
GTEX-Y8E4-1026
GTEX-Y8E5-0326
GTEX-Y8LW-2026
GTEX-Y9LG-1926
GTEX-YB5E-2226
GTEX-YB5K-2326
GTEX-YBZK-0326
GTEX-YEC3-2126
GTEX-YEC4-2226
GTEX-YF7O-2526
GTEX-YFC4-1026
GTEX-Z9EW-1726
GTEX-ZA64-2026
GTEX-ZAKK-0326
GTEX-ZC5H-0326
GTEX-ZDYS-1726
GTEX-ZPCL-2026
GTEX-ZPIC-2526
GTEX-ZQG8-1226
GTEX-ZQUD-1726
GTEX-ZT9X-1826
GTEX-ZTPG-0126
GTEX-ZTX8-1626
GTEX-ZV6S-2126
GTEX-ZV7C-2426
GTEX-ZVZO-0326
GTEX-ZVZP-2526
GTEX-ZY6K-2026
GTEX-ZYFG-2426
GTEX-ZYWO-2626
GTEX-ZZ64-1526

Whole blood

GTEX-113JC-0006-SM-5O997
GTEX-1192W-0005-SM-5NQBQ
GTEX-11DXX-0005-SM-5NQ8B
GTEX-11EMC-0006-SM-5O9DN
GTEX-11GSP-0006-SM-5N9EL
GTEX-11I78-0005-SM-5N9GB
GTEX-11LCK-0005-SM-5O98U
GTEX-11OF3-0006-SM-5O9CM
GTEX-11ONC-0005-SM-5O9CY
GTEX-11P7K-0006-SM-5N9FM
GTEX-11P82-0006-SM-5N9FY
GTEX-11TT1-0005-SM-5NQ8Y
GTEX-11VI4-0006-SM-5N9D8
GTEX-11WQK-0005-SM-5O9AV
GTEX-11ZTT-0006-SM-5N9FX
GTEX-1212Z-0006-SM-5NQ8M
GTEX-1269C-0005-SM-5N9CJ
GTEX-12C56-0006-SM-5N9E9
GTEX-12KS4-0005-SM-5SI94
GTEX-12WSI-0005-SM-5O99K
GTEX-12WSK-0006-SM-5NQA1
GTEX-12WSM-0005-SM-5NQB3
GTEX-12WSN-0006-SM-5NQAP
GTEX-12ZZX-0005-SM-5O9A9
GTEX-13113-0006-SM-5NQ7X
GTEX-1314G-0005-SM-5NQ9O
GTEX-131XE-0006-SM-5P9F9
GTEX-131XG-0006-SM-5O9CE
GTEX-132NY-0005-SM-5O9AC
GTEX-1399R-0006-SM-5N9FR
GTEX-139UW-0005-SM-5NQ8U
GTEX-13CF3-0006-SM-5N9ED
GTEX-13FTX-0005-SM-5N9F6
GTEX-13FXS-0006-SM-5O99X
GTEX-13OVG-0005-SM-5P9HA
GTEX-13OVH-0005-SM-5P9HB
GTEX-13OVI-0001-SM-5O9BL
GTEX-13OVK-0006-SM-5O9B7
GTEX-13OVL-0006-SM-5O996
GTEX-13OW6-0005-SM-5NQ9Z
GTEX-13OW8-0005-SM-5NQAC
GTEX-13PL7-0005-SM-5N9ET
GTEX-13S7M-0005-SM-5NQ76
GTEX-13VXT-0005-SM-5N9F3
GTEX-147F3-0005-SM-5N9FI
GTEX-147JS-0006-SM-5NQ7K
GTEX-148VI-0006-SM-5O9A6
GTEX-14A5H-0006-SM-5O9AI
GTEX-14AS3-0006-SM-5NQC2
GTEX-14B4R-0006-SM-5O9A7
GTEX-14BMV-0005-SM-5NQ6Y
GTEX-14C38-0006-SM-5NQBF
GTEX-14C39-0005-SM-5NQBR
GTEX-QEG5-0006-SM-2I5FZ
GTEX-QESD-0006-SM-2I5G6
GTEX-R55C-0005-SM-3GAE9
GTEX-RWS6-0005-SM-2XCAN
GTEX-S341-0006-SM-3NM8D
GTEX-SSA3-0005-SM-32QOT
GTEX-T5JW-0005-SM-3GADE
GTEX-T6MN-0005-SM-32PLJ
GTEX-T6MO-0006-SM-32QOU
GTEX-T8EM-0006-SM-3DB71
GTEX-TKQ1-0006-SM-33HBI
GTEX-TKQ2-0006-SM-33HBH
GTEX-TML8-0005-SM-32QPA
GTEX-TMZS-0006-SM-3DB8G
GTEX-U3ZG-0006-SM-47JWX
GTEX-U3ZH-0005-SM-3DB72
GTEX-U4B1-0006-SM-3DB8E
GTEX-UJMC-0005-SM-3GACU
GTEX-UPJH-0006-SM-3GACW
GTEX-V1D1-0006-SM-3NMCE
GTEX-V955-0005-SM-3P5ZC
GTEX-VJYA-0005-SM-3P5ZD
GTEX-VUSG-0006-SM-3GIK9
GTEX-WCDI-0005-SM-3NB2M
GTEX-WFG7-0005-SM-3GIKM
GTEX-WFON-0005-SM-3NMC9
GTEX-WH7G-0005-SM-3NMBX
GTEX-WHPG-0006-SM-3NMBV
GTEX-WHSB-0005-SM-3LK7C
GTEX-WHWD-0005-SM-3LK7D
GTEX-WOFL-0006-SM-3TW8K
GTEX-WOFM-0005-SM-3MJF3
GTEX-WQUQ-0006-SM-3MJF4
GTEX-WRHK-0005-SM-3MJF5
GTEX-WRHU-0006-SM-3MJF6
GTEX-WVLH-0006-SM-3MJF7
GTEX-WXYG-0005-SM-3NB3M
GTEX-WY7C-0006-SM-3NB3L
GTEX-WYVS-0006-SM-3NMA7
GTEX-WZTO-0006-SM-3NM9T
GTEX-X15G-0005-SM-3NMDA
GTEX-X3Y1-0006-SM-3P5ZG
GTEX-X5EB-0006-SM-46MV5
GTEX-X638-0005-SM-47JX6
GTEX-X88G-0006-SM-47JX5
GTEX-XBED-0006-SM-47JXO
GTEX-XBEW-0006-SM-4AT4E
GTEX-XMK1-0005-SM-4B665
GTEX-XPT6-0006-SM-4B66Q
GTEX-XXEK-0005-SM-4BRWJ
GTEX-XYKS-0005-SM-4BRUD
GTEX-Y114-0006-SM-4TT76
GTEX-Y5LM-0005-SM-4V6EJ

GTEX-14DAR-0006-SM-5N9GC
GTEX-14E1K-0006-SM-5N9DY
GTEX-14H4A-0006-SM-5N9E3
GTEX-14ICK-0006-SM-5NQB5
GTEX-14ICL-0006-SM-5SIAB
GTEX-N7MT-0007-SM-3GACQ
GTEX-O5YT-0007-SM-32PK7
GTEX-O5YW-0006-SM-3LK6E
GTEX-OHPL-0006-SM-3MJHB
GTEX-OIZF-0006-SM-2I5GQ
GTEX-OIZI-0005-SM-2XCED
GTEX-OXRP-0006-SM-2I3FN
GTEX-P4QS-0005-SM-2I3EY
GTEX-P78B-0005-SM-2I5GM
GTEX-PLZ5-0006-SM-5S2W5
GTEX-PLZ6-0006-SM-33HBZ
GTEX-POMQ-0006-SM-5SI7D
GTEX-PSDG-0005-SM-3GADC
GTEX-PVOW-0006-SM-3NMB8
GTEX-PW2O-0006-SM-2I3DV
GTEX-PWCY-0005-SM-33HBP
GTEX-Q2AG-0005-SM-5SI7F
GTEX-Q2AH-0005-SM-33HBR
GTEX-Q2AI-0006-SM-2I3FG
GTEX-QCQG-0006-SM-5SI8M
GTEX-Y5V5-0006-SM-4V6FE
GTEX-Y5V6-0005-SM-4V6FD
GTEX-Y8E4-0006-SM-4V6EW
GTEX-Y8E5-0006-SM-47JWQ
GTEX-Y8LW-0005-SM-4V6EV
GTEX-Y9LG-0006-SM-4VBRK
GTEX-YB5K-0005-SM-4VDSP
GTEX-YBZK-0005-SM-59HKG
GTEX-YFC4-0006-SM-4RGLV
GTEX-ZC5H-0005-SM-4WAXM
GTEX-ZDYS-0002-SM-4WKGR
GTEX-ZE9C-0006-SM-4WKG2
GTEX-ZF29-0006-SM-4WKGQ
GTEX-ZGAY-0006-SM-4WWAQ
GTEX-ZP4G-0006-SM-4WWE6
GTEX-ZPIC-0005-SM-4WWEB
GTEX-ZPU1-0006-SM-4WWAT
GTEX-ZQG8-0005-SM-4YCEH
GTEX-ZQUD-0005-SM-4YCE5
GTEX-ZVE2-0006-SM-51MRW
GTEX-ZVP2-0005-SM-51MRK
GTEX-ZVT2-0005-SM-57WBW
GTEX-ZVZP-0006-SM-51MSW
GTEX-ZXES-0005-SM-57WCB

EBV-transformed lymphocytes (LCLs)

GTEX-1122O-0003-SM-5Q5DL
GTEX-11EM3-0001-SM-5Q5BD
GTEX-11EMC-0002-SM-5Q5DO
GTEX-11OC5-0004-SM-5S2O6
GTEX-11P7K-0003-SM-5S2OU
GTEX-11TT1-0004-SM-5S2NT
GTEX-11VI4-0001-SM-5S2OI
GTEX-1212Z-0002-SM-5SI6W
GTEX-1269C-0003-SM-5S2PB
GTEX-12BJ1-0003-SM-5SI6V
GTEX-12C56-0002-SM-5S2PC
GTEX-RWS6-0001-SM-3NMAL
GTEX-S4Q7-0003-SM-3NM8M
GTEX-S95S-0002-SM-3NM8K
GTEX-SN8G-0001-SM-3NM8L
GTEX-T5JC-0001-SM-3NMAK
GTEX-T5JW-0003-SM-3NMAD
GTEX-T6MN-0002-SM-3NMAH
GTEX-T6MO-0003-SM-3NMAG
GTEX-TKQ1-0003-SM-3NMAE
GTEX-TML8-0001-SM-3NMAF
GTEX-U3ZH-0002-SM-3NMDD
GTEX-U3ZM-0002-SM-3NMDM
GTEX-U3ZN-0002-SM-3NMDF
GTEX-UPJH-0001-SM-3NMDE
GTEX-UPK5-0003-SM-3NMDI
GTEX-V1D1-0003-SM-3NMDP
GTEX-VJYA-0001-SM-3NMDJ
GTEX-VUSG-0003-SM-3NMDK
GTEX-W5WG-0002-SM-3NMDN
GTEX-W5X1-0001-SM-3P61V
GTEX-WFG7-0001-SM-3P61S
GTEX-WFG8-0001-SM-4LVN8
GTEX-WFJO-0002-SM-3P61X
GTEX-WFON-0001-SM-3P61W
GTEX-WHPG-0004-SM-3NMDO
GTEX-WHSB-0002-SM-4M1ZR
GTEX-WOFM-0001-SM-4OOT2
GTEX-WRHK-0001-SM-4WWDD
GTEX-WWTW-0002-SM-4MVNH
GTEX-WXYG-0004-SM-4MVOS
GTEX-WY7C-0004-SM-4ONDS
GTEX-WYVS-0004-SM-4ONDT
GTEX-WZTO-0001-SM-4PQZY
GTEX-X4LF-0002-SM-4QASG
GTEX-X5EB-0004-SM-46MWA
GTEX-XBED-0003-SM-47JWP
GTEX-XBEW-0002-SM-4AT5O
GTEX-XGQ4-0004-SM-4AT5S
GTEX-XMK1-0001-SM-4B64F
GTEX-XPT6-0001-SM-4B64G
GTEX-XQ3S-0001-SM-4B64K
GTEX-XXEK-0004-SM-4BRWO
GTEX-XYKS-0002-SM-4BRWN
GTEX-Y114-0002-SM-4TT78
GTEX-Y3IK-0001-SM-4WWE1
GTEX-Y5LM-0003-SM-4V6G1
GTEX-Y5V5-0001-SM-4V6FZ
GTEX-Y5V6-0003-SM-4V6FX
GTEX-Y8DK-0004-SM-4RGM7
GTEX-Y8E4-0003-SM-4V6FY
GTEX-Y9LG-0001-SM-4VBRQ
GTEX-YB5E-0001-SM-4VDSV
GTEX-YB5K-0003-SM-4VDSN
GTEX-YEC3-0002-SM-4W1YI
GTEX-YEC4-0002-SM-4W1Z6
GTEX-YF7O-0004-SM-4W1ZT
GTEX-YFCO-0003-SM-4W21I
GTEX-ZC5H-0004-SM-4WAXK
GTEX-ZDTS-0001-SM-4WAXW
GTEX-ZDTT-0004-SM-4WKG3
GTEX-ZEX8-0004-SM-4WKFQ
GTEX-ZF29-0002-SM-4WKF2
GTEX-ZF2S-0004-SM-4WKFE
GTEX-ZF3C-0001-SM-4WWAW
GTEX-ZG7Y-0003-SM-4WWEJ
GTEX-ZLWG-0004-SM-4WWD5
GTEX-ZP4G-0003-SM-4WWED
GTEX-ZPIC-0002-SM-4WWEC
GTEX-ZPU1-0004-SM-4WWAV
GTEX-ZQG8-0001-SM-4YCDH
GTEX-ZQUD-0003-SM-4YCD3
GTEX-ZT9W-0003-SM-4YCE6
GTEX-ZT9X-0004-SM-4YCDT
GTEX-ZTPG-0002-SM-4YCEI
GTEX-ZUA1-0002-SM-4YCF7
GTEX-ZV6S-0003-SM-4YCCT
GTEX-ZV7C-0003-SM-4YCF6
GTEX-ZVT2-0001-SM-57WCK
GTEX-ZVTK-0003-SM-51MRV
GTEX-ZVZP-0004-SM-51MS8

Cultured fibroblasts

GTEX-111YS-0008-SM-5Q5BH
GTEX-113JC-0008-SM-5QGR6
GTEX-117XS-0008-SM-5Q5DQ
GTEX-1192W-0008-SM-5QGRE
GTEX-11DXX-0008-SM-5Q5B8
GTEX-11DXY-0008-SM-5QGR4
GTEX-11EMC-0008-SM-5Q5DR
GTEX-11GSP-0008-SM-5Q5DM
GTEX-11I78-0008-SM-5Q5DI
GTEX-11LCK-0008-SM-5Q5BB
GTEX-11NSD-0008-SM-5Q5BC
GTEX-11NUK-0008-SM-5Q5B9
GTEX-11NV4-0008-SM-5Q5BA
GTEX-11O72-0008-SM-5Q5DN
GTEX-11OC5-0008-SM-5S2OH
GTEX-11OF3-0008-SM-5S2NH
GTEX-11ONC-0008-SM-5S2MG
GTEX-11P7K-0008-SM-5S2O5
GTEX-11P81-0008-SM-5S2OT
GTEX-11P82-0008-SM-5S2MS
GTEX-11PRG-0008-SM-5S2N5
GTEX-11TT1-0008-SM-5S2P8
GTEX-11TUW-0008-SM-5SI6S
GTEX-11WQC-0008-SM-5SI6R
GTEX-11WQK-0008-SM-5SI6T
GTEX-11XUK-0008-SM-5S2WD
GTEX-11ZTS-0008-SM-5S2VC
GTEX-11ZTT-0008-SM-5S2TZ
GTEX-11ZUS-0008-SM-5S2UO
GTEX-1211K-0008-SM-5S2W1
GTEX-12126-0008-SM-5S2UC
GTEX-12WSH-0008-SM-5S2V1
GTEX-12WSM-0008-SM-5S2VD
GTEX-1399U-0008-SM-5S2VE
GTEX-N7MS-0008-SM-4E3JI
GTEX-NFK9-0008-SM-4E3JE
GTEX-NL3G-0008-SM-4E3JX
GTEX-O5YT-0008-SM-4E3IQ
GTEX-O5YW-0008-SM-4E3IE
GTEX-OHPK-0008-SM-4E3JL
GTEX-OHPL-0008-SM-4E3I9
GTEX-OHPM-0008-SM-4E3IP
GTEX-OHPN-0008-SM-4E3HW
GTEX-OIZG-0008-SM-4E3J2
GTEX-OIZI-0008-SM-2XCFD
GTEX-OOBJ-0008-SM-3NB26
GTEX-OOBK-0008-SM-3NB27
GTEX-OXRK-0008-SM-3NB28
GTEX-T2IS-0008-SM-4DM75
GTEX-T5JC-0008-SM-4DM6A
GTEX-U4B1-0008-SM-4DXUW
GTEX-U8T8-0008-SM-4DXSP
GTEX-UJHI-0008-SM-4IHL1
GTEX-UJMC-0008-SM-4IHKK
GTEX-UPK5-0008-SM-4IHJD
GTEX-V1D1-0008-SM-4JBIJ
GTEX-W5X1-0008-SM-4LMKA
GTEX-WFG7-0008-SM-4LMKB
GTEX-WHPG-0008-SM-4M1ZQ
GTEX-WHSB-0008-SM-4M1ZP
GTEX-WHWD-0008-SM-4OOSU
GTEX-WI4N-0008-SM-4OOSV
GTEX-WL46-0008-SM-4OOSW
GTEX-WQUQ-0008-SM-4OOT1
GTEX-WRHU-0008-SM-4MVPB
GTEX-WVJS-0008-SM-4MVPC
GTEX-WVLH-0008-SM-4MVPD
GTEX-WY7C-0008-SM-4ONDW
GTEX-WYBS-0008-SM-4ONDX
GTEX-WYJK-0008-SM-4ONDV
GTEX-WYVS-0008-SM-4ONDY
GTEX-WZTO-0008-SM-4PQZZ
GTEX-X15G-0008-SM-4PR2D
GTEX-X3Y1-0008-SM-4PR12
GTEX-X4LF-0008-SM-4QAST
GTEX-XBEC-0008-SM-4AT3X
GTEX-XBEW-0008-SM-4AT3Y
GTEX-XMD2-0008-SM-4WWE7
GTEX-XMD3-0008-SM-4AT4V
GTEX-XMK1-0008-SM-4GICF
GTEX-XOT4-0008-SM-4B664
GTEX-XPT6-0008-SM-4B64Q
GTEX-XPVG-0008-SM-4GICH
GTEX-XQ3S-0008-SM-4GIDZ
GTEX-XUW1-0008-SM-4BOQH
GTEX-XV7Q-0008-SM-4BRWL
GTEX-Y8E4-0008-SM-4V6FW
GTEX-Y9LG-0008-SM-4VBRJ
GTEX-YB5K-0008-SM-4VDT8
GTEX-YEC4-0008-SM-4W1YR
GTEX-YF7O-0008-SM-4W1ZS
GTEX-YJ89-0008-SM-4RGM4
GTEX-Z93S-0008-SM-4RGM5
GTEX-ZC5H-0008-SM-4WAX8
GTEX-ZDTS-0008-SM-4E3I8
GTEX-ZDTT-0008-SM-4E3K5

GTEX-OXRL-0008-SM-3NB29
GTEX-P4PP-0008-SM-48TDV
GTEX-P4QT-0008-SM-48TDZ
GTEX-PSDG-0008-SM-48TE5
GTEX-PW2O-0008-SM-48TEB
GTEX-PWCY-0008-SM-48TE9
GTEX-PX3G-0008-SM-48U2L
GTEX-Q2AH-0008-SM-48U2J
GTEX-QCQG-0008-SM-48U2G
GTEX-QLQ7-0008-SM-447AW
GTEX-QXCU-0008-SM-48FCH
GTEX-R45C-0008-SM-48FF2
GTEX-R55C-0008-SM-48FCF
GTEX-R55D-0008-SM-48FEV
GTEX-R55E-0008-SM-48FCG
GTEX-R55G-0008-SM-48FEX
GTEX-RM2N-0008-SM-48FF3
GTEX-RN64-0008-SM-48FEZ
GTEX-RNOR-0008-SM-48FEY
GTEX-RU1J-0008-SM-46MV9
GTEX-RU72-0008-SM-46MV8
GTEX-RWS6-0008-SM-47JYV
GTEX-RWSA-0008-SM-47JYX
GTEX-S33H-0008-SM-4AD6C
GTEX-S4Z8-0008-SM-33HAZ
GTEX-SE5C-0008-SM-4B64J
GTEX-SJXC-0008-SM-4DM7G
GTEX-ZDXO-0008-SM-4E3HR
GTEX-ZDYS-0008-SM-4E3IX
GTEX-ZE7O-0008-SM-4E3JQ
GTEX-ZEX8-0008-SM-4E3JU
GTEX-ZF2S-0008-SM-4E3IK
GTEX-ZF3C-0008-SM-4E3IL
GTEX-ZLWG-0008-SM-4E3J4
GTEX-ZP4G-0008-SM-4E3I4
GTEX-ZPIC-0008-SM-4E3JF
GTEX-ZPU1-0008-SM-4E3IR
GTEX-ZQG8-0008-SM-4E3J9
GTEX-ZQUD-0008-SM-4YCCU
GTEX-ZT9W-0008-SM-4YCDJ
GTEX-ZT9X-0008-SM-4YCD7
GTEX-ZTPG-0008-SM-4YCEK
GTEX-ZTX8-0008-SM-4YCDV
GTEX-ZUA1-0008-SM-4YCEW
GTEX-ZV68-0008-SM-4YCCV
GTEX-ZV6S-0008-SM-4YCF9
GTEX-ZV7C-0008-SM-57WCL
GTEX-ZVE2-0008-SM-51MRU
GTEX-ZVP2-0008-SM-51MSL
GTEX-ZVT2-0008-SM-57WC9
GTEX-ZVT3-0008-SM-51MRI
GTEX-ZVTK-0008-SM-57WDA
GTEX-ZVZP-0008-SM-51MSX
GTEX-ZXES-0008-SM-57WCX

Supplementary Results

Minimum required sequencing depth (MRSD) scores differ across biosamples

For all but one parameter combination, moving from $MRSD_{0.95}$ to $MRSD_{0.99}$ resulted in an increase in median MRSD of between 26.19-155.40%. However, when stipulating 95% splice junction coverage for skeletal muscle samples, we observed a decrease of 4.66% in MRSD scores for $MRSD_{0.95}$ (*n* =1323, median = 42.52) compared to $MRSD_{0.99}$ (*n* = 973, median = 40.54); this was accounted for by an increase in the number of genes that were considered “unfeasible” for surveillance, i.e. those for which zero reads cover the given proportion of junctions (*n* unfeasible $MRSD_{0.95}$ = 1873, *n* unfeasible $MRSD_{0.99}$ = 2193). This definition of feasibility is limited by the sequencing depth of the reference sets on which the predictions are based. Ultra-deep sequencing of the same reference sets, may have enabled feasible MRSD predictions for an increased number of splicing junctions.

Impact of read length on MRSD accuracy

To assess whether the MRSD scores themselves were altered through derivation from 75 bp or 150 bp RNA-seq reference sets, we generated paired MRSD scores from datasets that were trimmed from 150 bp to 75 bp reads (Figure S7). We were able to calculate MRSD scores for 54.2% of multi-exon disease-associated genes (1802/3322) from these datasets. 86.5% (243/1802) of observable genes had lower MRSD scores from 150 bp read reference sets than from 75 bp read reference sets, or were only feasible in 150 bp reference sets. 13.5% (243/1802) counter-intuitively exhibited a higher MRSD in the 150 bp dataset, suggesting that fewer 75 bp reads were required to adequately cover these transcripts. In many examples, this could be attributed to a decrease in mapping quality of longer reads such that the reads did

not pass the quality filters of the employed pipeline[13]. Further work is needed to ascertain whether this discarding of longer reads is a harmful artefact of the filtering process, or a genuine removal of uninformative reads.

Comparison of MRSD and TPM as a guide for appropriate surveillance

We noted significant overlap between genes grouped into low-MRSD (< 100 M reads) and high-MRSD (≥ 100 M reads) brackets. For example, among genes considered low-MRSD, TPM values ranged from 0.99-246,600, while genes with high-MRSD values had TPM values between 0.20-8644 (Figure 3D). We quantified the overlap between these distributions, demonstrating that, depending on the tissue, between 98.0% and 99.3% of high-MRSD genes had higher TPM values than at least one low-MRSD gene. We also observed that, in their respective tissues, the TPMs of 44.1-60.0%, 8.5-16.7% and 3.4-6.6% of high-MRSD genes exceeded those of the 5%, 30% and 50% least-expressed low-MRSD genes, respectively (Figure 3D). The substantial overlap in the TPM values for low and high MRSD genes suggests that relative expression does not provide a wholly accurate representation of transcript coverage in RNA-seq data. Such inconsistencies may arise from bias in the regions of genes that are sequenced, for example, genes with high degrees of 3' bias in RNA-seq datasets or significant alternative transcript usage (Figure S8).

Factors influencing the likelihood of pathogenic splicing variation identification & MRSD predictions

To further define the most informative parameters for use in the MRSD model, we investigated the impact of a variety of metrics on the capability to identify pathogenic splicing events, including number of samples within the healthy reference set, the degree of read support for splicing junctions, and the relative expression of genes of interest. We aimed to quantify the effect of changes in these metrics on both the total number of events of interest and the position within the list of events (see Materials and Methods for filtering and ranking strategy).

We first identified how the number of control samples used as a reference set for “healthy splicing” impacted our ability to identify aberrant splicing events. For all samples within our healthy splicing set, we iteratively selected groups of control samples at sizes of 30, 60 or 90. We observed that moving from 30 to 60 controls is associated with a mean reduction in event count of 19.3% (28.1% of non-singleton events, 17.1% of singleton events) across the three tissues, while increasing the control size to 90 results in a further reduction of 10.2% of events (16.5% of non-singleton events, 9.5% of singleton events; Figure 4); this effect was consistent across tissue types.

We next investigated how read count filters impacted the number of events observed for a given individual (Figure 4). Filtering out all splicing events supported by just a single read against a background of 90 control samples removes, on average, 91.2% of events (60.4% of non-singleton events, 97.3% of singleton events). Increasing read support thresholds to 10 unique sequencing reads results in a total of 99.4% of

events being excluded on average (96.2% of non-singleton events, 99.99% of singleton events), while retaining only those events supported by 100 reads or more removes an average of 99.97% of events (99.8% of non-singleton events, 100.0% of singleton events). To understand how the level of read support impacted the ability to identify specific events, we collated 31 aberrant splicing events across 22 muscle-derived RNA-seq samples, and downsampled reads in the genes containing these events. We observed that we could identify the same aberrant splicing events at reduced relative expression levels, and, while read support decreased (Figure 5A), the ranked position of the event within the rank-ordered output remained approximately the same in most cases (Figure 5B). However, the weakened read support increased the risk of eliminating the variant from consideration when read count filters were applied (Figure 5C). This analysis further emphasized that TPM values alone may not be a reliable measure of ability to survey all splicing junctions within a gene; we observed that splice junctions in different samples covered by the same number of sequencing reads belonged to genes with widely ranging TPM values (Figure S10). For example, splice junctions covered by eight reads were identified in genes with TPMs ranging between 0.17 and 52.

Based on these investigations, we selected an eight-read coverage value for downstream analyses; as we observed that the majority of pathogenic mis-splicing events have an NRC ≥ 0.25, stipulating an eight-read coverage requirement means that aberrant events should be covered by at least two reads, and so be retained when filtering single-read events from the list of splicing events. We appreciate that the use of more stringent parameters may be preferable in some use cases, such as to generate sufficient corroboration to support the reporting of a diagnostic finding to

a patient or when using significance-based tools such as FRASER, LeafCutterMD and SPOT. However, our investigations have shown this approach to be robust for the initial highlighting of aberrant splicing events for downstream analysis.

References

1. Cummings, B.B., Marshall, J.L., Tukiainen, T., Lek, M., Donkervoort, S., Foley, A.R., et al. Improving genetic diagnosis in Mendelian disease with transcriptome sequencing. (2017). Sci Transl Med. *9(386)*.
2. The GTEx Consortium. (2013). The Genotype-Tissue Expression (GTEx) project. Nat Genet. *45*(6), 580-585.
3. Dobin, A., Davis, C.A., Schlesinger, F., Drenkow, J., Zaleski, C., Jha, S., Batut, P., Chaisson, M., and Gingeras, T.R. (2013). STAR: ultrafast universal RNA-seq aligner. Bioinformatics. *29(1)*, 15-21.
4. Jaganathan, K., Kyriazopoulou Panagiotopoulou, S., McRae, J.F., Darbandi, S.F., Knowles, D., Li, Y.I., et al. (2019) Predicting Splicing from Primary Sequence with Deep Learning. Cell. *176(3)*:535-48.e24.
